# Retinol tracing within murine neural retina reveals cell type–specific retinol transport and distribution

**DOI:** 10.1172/JCI198648

**Published:** 2025-11-18

**Authors:** Zachary J. Engfer, Grazyna Palczewska, Samuel W. Du, Jianye Zhang, Zhiqian Dong, Carolline Rodrigues Menezes, Jun Wang, Jianming Shao, Budd A. Tucker, Robert F. Mullins, Rui Chen, Philip D. Kiser, Krzysztof Palczewski

**Affiliations:** 1 Brunson Center for Translational Vision Research, Department of Ophthalmology and Visual Sciences, Gavin Herbert Eye Institute, and; 2 Department of Physiology & Biophysics, University of California, Irvine, Irvine, California, USA.; 3 Department of Molecular and Human Genetics, Baylor College of Medicine, Houston, Texas, USA.; 4 Department of Ophthalmology and Visual Sciences, University of Iowa, Iowa City, Iowa, USA.; 5 Department of Clinical Pharmacy Practice, University of California, Irvine, Irvine, California, USA.; 6 Research Service, VA Long Beach Healthcare System, Long Beach, California, USA.; 7 Department of Chemistry and; 8 Department of Molecular Biology and Biochemistry, University of California, Irvine, Irvine, California, USA.

**Keywords:** Cell biology, Ophthalmology, Retinopathy

## Abstract

11-*cis*-Retinal is essential for light perception in mammalian photoreceptors (PRs), and aberrations in retinoid transformations cause severe retinal diseases. Understanding these processes is crucial for combating blinding diseases. The visual cycle, operating within PRs and the retinal pigment epithelium (RPE), regenerates 11-*cis*-retinal to sustain light sensitivity. Retinoids are also present in Müller glia (MG), hypothesized to supply 11-*cis*-retinol to cone PRs and retinal ganglion cells (RGCs). To trace retinoid movement through retinal cell types, we used cell-specific knockin of lecithin:retinol acyltransferase (LRAT), which converts retinols into stable retinyl esters (REs). Ectopic LRAT expression in murine PRs, MG, and RGCs resulted in RE synthesis, with REs differing in abundance and isomeric composition across cell types under genetic and light-based perturbations. PR inner segments showed high 11-*cis*-RE content, suggesting a constant 11-*cis*-retinoid supply for pigment regeneration. In MG expressing LRAT, all-*trans*-REs were detected, contrasting with 11-*cis*-REs in PRs. The MG-specific LRAT phenotype mirrored the RE-rich human neural retina, suggesting human MG may utilize LRAT to maintain retinoid reservoirs. Our findings reveal tightly controlled retinoid flux throughout the mammalian retina that supports sustained vision, expanding understanding of the visual cycle to combat retinal diseases.

## Abstract 2

These studies are fundamental for understanding our vision and are also key in designing strategies to combat inherited and acquired retinal diseases.

## Graphical abstract

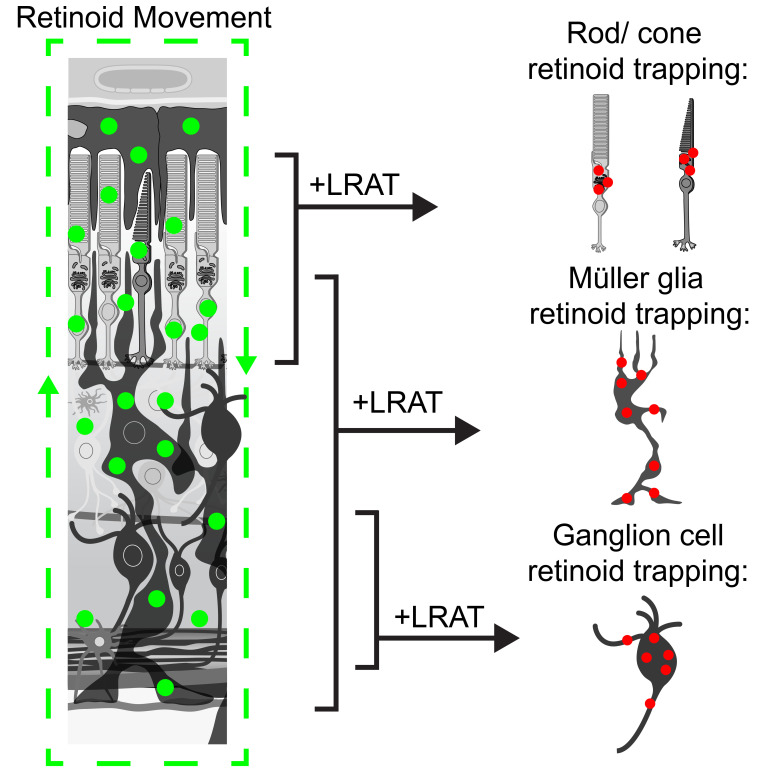

## Introduction

Retinoids are derivatives of vitamin A (all-*trans*-retinol) that are essential for vision and retinal health (1, 2). 11-*cis*-Retinal is the essential visual chromophore that responds to incoming light within the mammalian retina; it is loaded onto densely packed opsin proteins in the outer segments of the rod and cone photoreceptors (PRs) (3–5). Photoisomerization of each opsin-bound 11-*cis*-retinylidene chromophore to all-*trans*-retinylidene initiates a signaling cascade in the PRs known as phototransduction, generating electrochemical signals that are relayed from the PRs to the brain through several intermediary retinal cell types (6, 7). The all-*trans*-retinylidene is then hydrolytically cleaved from the opsin, and the released all-*trans*-retinal must be converted back to 11-*cis*-retinal to restore the light-sensitivity of PRs (3, 8). Regeneration of 11-*cis*-retinal occurs through a multistep pathway known as the visual cycle (1), which involves complementary enzymes that process distinct biochemical intermediates in the PRs and retinal pigment epithelium (RPE). Retinol dehydrogenase (RDH) enzymes in the PRs catalyze the reduction of all-*trans*-retinal to all-*trans*-retinol, which diffuses and is trapped in the RPE for esterification by lecithin:retinol acyltransferase (LRAT) (9). The resulting all-*trans*-retinyl esters (all-*trans*-REs) are the most stable visual cycle intermediates, and they are stored in self-aggregating reservoir structures known as retinosomes within the RPE (9–12). In response to the need for 11-*cis*-retinal, all-*trans*-REs from the retinosomes are hydrolyzed and isomerized to 11-*cis*-retinol by the enzyme RPE65, and the 11-*cis*-retinol is oxidized to 11-*cis*-retinal by RPE-localized RDHs, such as RDH5 (1). The 11-*cis*-retinal then diffuses back to the PRs to re-form visual pigments and sustain light sensitivity within the PRs. There is also evidence for an alternative visual chromophore regeneration pathway in the RPE and Müller glia (MG), involving RGR-opsin and retinaldehyde-binding protein 1 (RLBP1; also known as cellular retinaldehyde-binding protein [CRALBP]), which are expressed in both cell types (13–15). RGR has been shown to catalyze the photoisomerization of all-*trans*-retinal to 11-*cis*-retinal upon stimulation with visible light (16, 17). Both proteins in MG could assist in providing chromophore from the outer retina to intrinsically photosensitive retinal ganglion cells (ipRGCs), which use 11-*cis*-retinal for nonvisual light sensing using melanopsin (18).

Mutations in genes that encode proteins involved in the synthesis of 11-*cis*-retinal, import of 11-*cis*-retinal precursors to the retina, and regeneration of 11-*cis*-retinal are linked to inherited retinal diseases (IRDs) that lead to blindness, including retinitis pigmentosa, Leber congenital amaurosis, and Stargardt disease (19). Therefore, understanding the biodistribution of retinoids at different stages of the visual cycle is imperative for elucidating the pathogenesis of IRDs stemming from mutations in visual cycle–associated genes. The development of genome-editing therapeutics offers promise for permanent correction of some underlying genetic causes of IRDs (20, 21). In many cases, however, additional visual cycle modulation will be needed to halt the progression of active retinal degeneration, especially in retinas that have been chronically poisoned by excess all-*trans*-retinal and its condensation byproducts such as the pyridinium bisretinoid A2E and others (22, 23). Accordingly, there is an interest in tracking changes in retinoid distribution throughout different cellular compartments of the retina in healthy and diseased states. Such comprehensive retinoid analysis is challenging, given the low concentration and transience of certain retinoid intermediates coupled with the lack of spatial resolution, particularly within cells of the inner retina (e.g., MG or RGCs).

**Figure 1:**
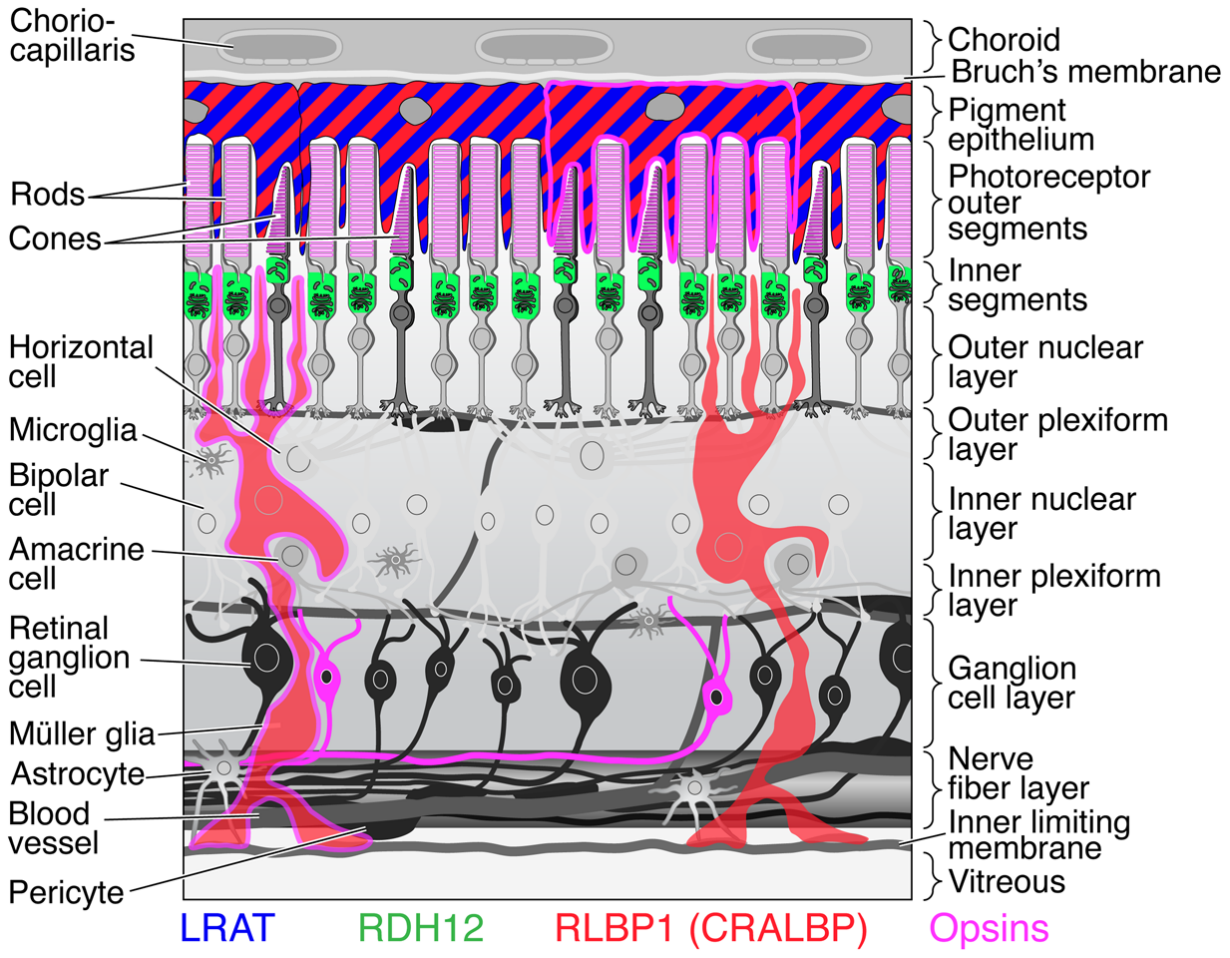
Retinoid-related protein expression in mouse retina. Rhodopsin is concentrated in the outer segments of rod PRs; cone opsins are in the outer segments of cone PRs; melanopsin is found in a subset of RGCs; RGR-opsin is found in a subset of MG and the RPE cells (collectively called opsins); LRAT is predominantly expressed in the RPE; CRALBP is localized in both the RPE and MG; and RDH12 in the PR-IS. Not depicted are other retinol/ alcohol dehydrogenases, including RDH8 expressed in PRs and RDH5 expressed in the RPE cells; STRA6 (stimulated by retinoid acid 6), a membrane transporter and a cell surface receptor for RBP4, expressed in the RPE; retinol-binding protein 3 (RBP3 or IRBP), found primarily in the interphotoreceptor matrix of the retina between the RPE and the PRs; cellular retinol-binding proteins (such as RBP1), involved in intracellular protein transport for uptake and metabolism; and retinoic acid receptors related proteins (37).

In this study, we tracked retinoid distribution across retinal cell types using cell-selective LRAT expression. LRAT under PR-, MG-, and RGC-specific promoters sequestered retinols, forming stable REs distinct from those in the RPE. RE isomeric composition changed with genetic ablation of retinoid-handling proteins (RDH12, CRALBP) (Figure 1). These REs were visualized by 2-photon microscopy (TP) and formed retinosome-like structures in cell types normally lacking retinosomes.

## Results

### Ectopic expression of LRAT in PR-Lrat^+^ animals.

**Figure 2:**
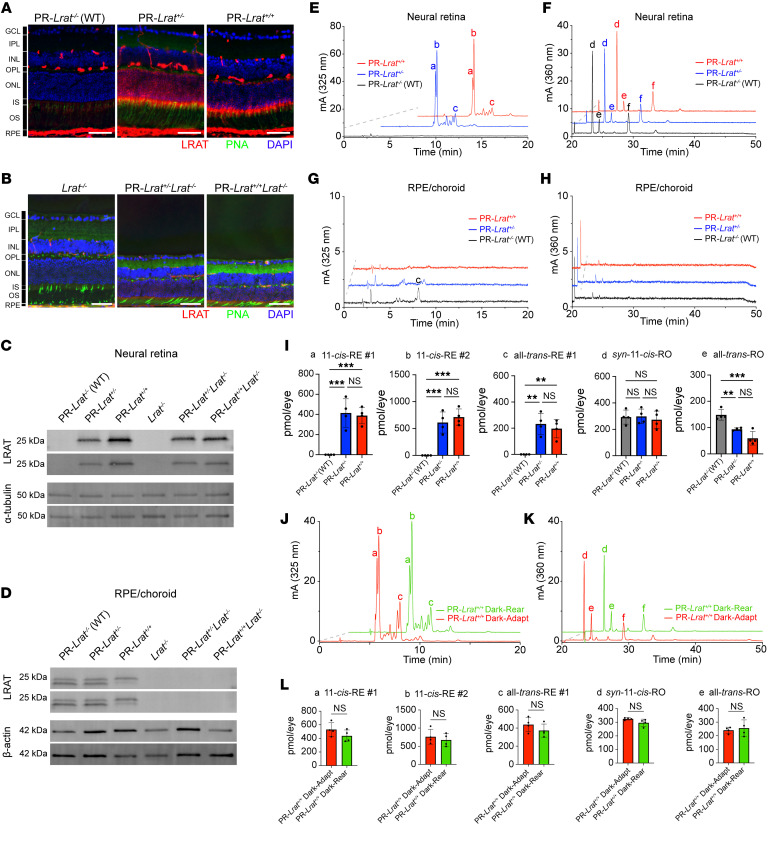
Expression of Lrat in mouse PR. (**A**) IF staining of the retina with LRAT and PNA in pigmented 12-week-old mice (scale bar: 50 μm). Contrast levels were uniformly enhanced across images to enhance the legibility of each channel (LRAT and PNA) in the figure. Raw images were taken at the same exposure settings across genotypes. (*n* = 2 per genotype). Images were taken at ~400–800 μm from the optic nerve (original magnification, ×40). (**B**) IF staining of retina with LRAT (red channel) and PNA (green channel) in PFA-fixed cryosections from 12-week-old albino mice (scale bar: 50 μm). LRAT and PNA signals enhanced (*n* = 4 *Lrat^–/–^*; *n* = 3 *PR-Lrat^+^Lrat^–/–^* mice). Images were taken at ~800 μm from the optic nerve (original magnification, ×20). (**C**) Immunoblotting of neural retinas (6- to 7-week-old mice; *n* = 2). (**D**) Immunoblotting of RPE and choroid (6- to 7-week-old mice; *n* = 2). (**E** and **F**) HPLC traces of neural retinas from 6- to 7-week-old DA mice (~10 hours), showing the RE-elution time frame (**E**) and RO-elution time frame (**F**). (**G** and **H**) HPLC traces of RPE/choroid from 6- to 7-week-old DA mice. (**I**) Quantification of retinoids in neural retina retinoid extracts from mice. (**J** and **K**) HPLC traces of whole-eye retinoid extracts from 6-week-old DA (~10 hours, red trace) and dark-reared (DR, green trace) *PR-Lrat^+/+^* mice at the RE-elution time frame (**J**) and RO-elution time frame (**K**). (**L**) Quantification of retinoids in whole-eye retinoid extracts from 6-week-old DA (red) and DR (green) mice. In **E**–**H**, **J**, and **K**, a and b, 11-*cis*-RE peaks; c, all-*trans*-RE peaks; d, syn-11-*cis*-RO peaks; e, syn-all-*trans*-RO peaks; and f, anti-11-*cis*-RO peaks. *P ≤ 0.05; **P ≤ 0.01; ***P ≤ 0.001; ****P ≤ 0.0001, 1-way ANOVA (**I**) and unpaired *t* test (**L**). Data are shown as the mean ± SD.

Given the stability of REs and the robust catalytic activity of LRAT, we hypothesized that we could leverage ectopic expression of LRAT to see which retinol isomers reach different cellular compartments of the neural retina. We predicted that we would see 11-*cis*-RE isomers rather than all-*trans*-RE isomers in MG if they participate in alternative visual chromophore regeneration. As a proof of concept, we first wanted to test our LRAT monitoring system in retinoid-rich PRs. Using PR-*Lrat* mice that express murine LRAT under the native PR-specific *Gcap1* locus (Supplemental Figure 1; supplemental material available online with this article; https://doi.org/10.1172/JCI198648DS1), we examined the extent and localization of LRAT protein production in PR-*Lrat^+^* PRs. LRAT immunofluorescent (IF) staining of retinal cryosections from PR-*Lrat^–/–^* (WT), PR-*Lrat^+/–^*, and PR-*Lrat^+/+^* mice revealed LRAT protein in the RPEs of each genotype, with a second band of LRAT signal selectively localizing to the PR inner segments (PR-IS) in PR-*Lrat^+/–^* and PR-*Lrat^+/+^* cryosections (Figure 2A). We confirmed that LRAT staining was eliminated from the RPEs of PR-*Lrat^+^Lrat^–/–^* mice (which lack native LRAT expression), but it still appeared in the PR-IS (Figure 2B). A single prominent band corresponding to LRAT (molecular weight = 26 kDa) appeared in blots of neural retinal homogenates from the PR-*Lrat^+^* and PR-*Lrat^+^Lrat^–/–^* mice, but no signal was evident in the corresponding lanes for WT and *Lrat^–/–^* controls (Figure 2C). Two bands corresponding to LRAT (24) were present in blots of RPE/choroid homogenates from PR-*Lrat^–/–^* (WT), PR-*Lrat^+/–^*, and PR-*Lrat^+/+^* mice, with no signal in the corresponding lanes for *Lrat^–/–^*, PR-*Lrat^+/–^Lrat^–/–^*, and PR-*Lrat^+/+^Lrat^–/–^* mice (Figure 2D). Therefore, LRAT is expressed in the PRs of PR-*Lrat^+^* mice, even in the absence of native LRAT expression in the RPE.

We assessed whether introduction of LRAT into PRs resulted in retinal degeneration. Using optical coherence tomography (OCT) and histology on C57BL/6J (WT), PR-*Lrat^+/–^*, and PR-*Lrat^+/+^* animals, we evaluated retinal structure. OCT indicated that PR-*Lrat^+^* mice had intact outer nuclear layers (ONLs), though the definition of the outer limiting membrane (OLM) and ONL borders was decreased compared with that of the WT controls (Supplemental Figure 2A). Scanning laser ophthalmoscopy (SLO) revealed only small autofluorescent spots localized to the outer retinal layers of PR-*Lrat^+^* mice (Supplemental Figure 2B). H&E staining of retinal cryosections showed no substantial variations in WT control and PR-*Lrat^+^* nuclei counts at 4 weeks of age and minimal changes at 12 weeks. We observed progressive ONL thinning in PR-*Lrat^+/–^* mice and more advanced thinning in PR-*Lrat^+/+^* mice by 26 weeks of age, indicating that the introduction of the PR-*Lrat* allele resulted in a slow, progressive loss of PRs (Supplemental Figure 2, C and D). This was more pronounced when examining the histology of the PR-*Lrat^+^Lrat^–/–^* mice compared with that of the *Lrat^–/–^* controls in Figure 2B. We chose to focus mostly on 6- to 8-week-old mice for downstream phenotyping experiments, because mice at this age have fully mature retinas and there is negligible loss of PR nuclei relative to that of age-matched WT animals.

We also attempted the same knockin strategy for PR-localized RPE65 expression under the *Gcap1* promoter to test whether RPE-localized enzymes of the visual cycle could be coexpressed in PRs. We detected an *Rpe65* transcript in the neural retinas of PR-*Rpe65^+/+^* mice via PCR (Supplemental Figure 3C), but RPE65 protein was undetectable via IF (Supplemental 3A) or immunoblotting (Supplemental Figure 3B). This result indicated that there are differences in the conditions necessary for functional expression of LRAT and RPE65; therefore, we discontinued this line of study.

### Ectopic expression of LRAT in PRs effectively traps distinct retinoid species within the PR-IS.

To examine whether PR-localized LRAT is biochemically active and capable of synthesizing REs, we performed HPLC-based retinoid analyses on mouse extracts from neural retina and RPE/choroid tissues (Figure 2, E–I). We identified unique peaks eluting within the RE time frame in neural retina extracts from dark-adapted (DA) (~10 h) PR-*Lrat^+/–^* and PR-*Lrat^+/+^* mice (Figure 2E). These peaks exceeded all-*trans*-retinyl palmitate in the RPE of WT mice. The absorbance characteristics of the 2 predominant REs in the neural retinal extracts from PR-*Lrat^+^* mice matched the published λ*_max_* values of 11-*cis*-retinyl palmitate (318 nm), which is normally absent from the retina and RPE of WT mice (25). There were also smaller, later eluting peaks with λ*_max_* values matching the published value of all-*trans*-retinyl palmitate (25). Examining the peaks corresponding to *syn*-11-*cis*-retinal oxime and all-*trans*-retinal oxime, we found no marked differences in visual chromophore content in neural retina extracts of WT and PR-*Lrat^+^* mice (Figure 2F). REs in RPE/choroid extracts from WT and PR-*Lrat^+^* mice were minimal, with small, quantifiable all-*trans*-retinyl palmitate peaks in WT extracts. No appreciable visual chromophore was present in RPE/choroid extracts (Figure 2H). The earlier-eluting 11-*cis*-RE averaged 414.4 pmol/eye in neural retina extracts from PR-*Lrat*^+/–^ mice and 389.2 pmol/eye in neural retina extracts from PR-*Lrat*^+/+^ mice; the second, later-eluting 11-*cis*-RE averaged 612.1 pmol/eye in neural retina extracts from PR-*Lrat^+/–^* mice and 713.3 pmol/eye in neural retina extracts from PR*-Lrat*^+/+^ mice. These two 11-*cis*-REs were absent in extracts from WT control mice. There was also a significant increase in neural retina-localized all-*trans*-retinyl palmitate content in each PR-*Lrat^+^* genotype compared with the WT control, with an average of 232.9 pmol/eye more all-*trans*-retinyl palmitate in PR-*Lrat^+^* neural retina extracts (*P_adj_* = 0.0013) and an average of 196.4 pmol/eye more all-*trans*-retinyl palmitate in PR-*Lrat^+/+^* neural retina extracts compared with the WT controls (Figure 2I; *P_adj_* = 0.004). To determine whether PR-*Lrat^+^* neural retinal RE accumulation was a light-dependent process, we performed whole-eye retinoid analyses on 6-week-old dark-reared and DA (~10 hours) PR-*Lrat^+/+^* mice (Figure 2, J–L). There were no significant differences in the measured retinoid species, indicating that accumulation of 11-*cis*- and all-*trans*-REs occurred in PR-*Lrat^+/+^* mice regardless of visual cycle progression and circadian light exposure (Figure 2L; *P_adj_* > 0.05).

We subjected purified PR-*Lrat^+/–^* REs to alkaline hydrolysis, which releases the corresponding retinol and fatty acid moieties (Supplemental Figure 4, A–C). We found that the absorbance spectrum of the retinol (Supplemental Figure 4C), with λ_max_ = 316 nm, was close to that of 11-*cis*-retinol (λ_max_ = 318 nm). We also investigated whether the 2 predominant 11-*cis*-RE peaks found in PR-*Lrat^+^* whole-eye retinoid extracts had fatty acid tails reflecting the ratio of available *sn*-1 acyl donors on PCs present in PR membranes; i.e., predominantly stearoyl and palmitoyl moieties (26). Using LC-ESI-MS**,** [M˙]^+^ ions of the REs (27) and all-*trans*-retinyl palmitate standard were detected at concentrations below 0.5 pmol/injection. Separation of the pooled extracts from PR-*Lrat^+/–^* mice on a C18 column resulted in 2 major peaks (at 7.65 and 9.23 min), both with λ_max_ = 320 nm (Supplemental Figure 4, D and E), corresponding to the 2 major 11-*cis*-RE peaks (at 6–7 min) under normal-phase HPLC. MS analysis identified these 2 11-*cis*-REs as retinyl palmitate (7.65 min, *m*/*z* 524.46, 0.4 ppm) and retinyl stearate (9.23 min, m/z 552.49, 0.9 ppm). The 11-*cis*-REs eluting slightly later than all-*trans*-retinyl palmitate (7.60 min) on reverse-phase HPLC are also consistent with their *cis* conformation. Thus, we demonstrated that the 2 predominant REs were 11-*cis* isomers.

### Effects of ectopic expression of LRAT on PR visual cycle kinetics, electrophysiology, and distribution of cone cells.

We also wanted to assess whether our LRAT-based retinoid monitoring system perturbed visual cycle kinetics and PR electrophysiological responses in young (8-week-old) animals, which would complicate our conclusions about what retinoid isomers are normally present within PR-IS. To determine whether ectopic expression of LRAT in PRs affected regeneration of 11-*cis*-retinal following bleaching, we performed retinoid analyses on extracts from whole eyes from 8-week-old mice. *Syn*-11-*cis*-RO levels did not significantly differ between time point–matched WT and PR-*Lrat^+^* mice, indicating no substantial changes in visual cycle kinetics with the introduction of LRAT into PRs (Supplemental Figure 5). 11-*cis*-RE levels decreased after bleaching and during recovery in DA PR-*Lrat^+/+^* mice, possibly due to mobilization of PR-REs.

When we examined rod- and cone-specific responses to light using electroretinography (ERG), we found that there were no marked differences in amplitudes between WT and PR-*Lrat^+^* mice (Supplemental Figure 6, A–G). We also examined the distribution of cones in neural retina flat mounts from *Lrat^–/–^*, PR-*Lrat^+/–^Lrat^–/–^*, and PR-*Lrat^+/+^Lrat^–/–^* mice. We found that there was no effect of the PR-*Lrat^+^* transgene on the morphology and distribution of M- and S-cones; each genotype displayed the *Lrat^–/–^*-characteristic loss of cones in the inferior region by 12 weeks of age (Supplemental Figure 6, H–J).

### Effects of successive light exposure on the ratio of 11-cis-RE — all-trans-RE species in PR-Lrat^+^ mice.

Given our findings that suggest that ectopic LRAT has minimal effects on PR visual cycle kinetics, chromophore content, and electrophysiology, we wanted to see if the system could capture transient perturbations in retinoids by subjecting PR-*Lrat^+/–^* mice to successive bleaches with light and seeing if they can sequester transiently elevated all-*trans*-retinol as REs after bleaching with light. To test the responsiveness of our system, we subjected PR-*Lrat^+/–^* mice to twice-daily bleaches (10,000 lux for 5 minutes) to isomerize the opsin-bound 11-*cis*-retinylidene. After 24 hours DA of the bleached animals, ) we harvested pairs of whole eyes from each animal, extracted the retinoids, and analyzed them (Supplemental Figure 7, A–H). We found that successive bleaching of PR-*Lrat^+/–^* animals led to a significantly decreased ratio of summed 11-*cis*-retinoid species to all-*trans* retinoid species (predicted least-squares [LS] mean difference = 3.601, *P* < 0.0001) relative to that of DA, unbleached PR-*Lrat^+/–^* animals (Supplemental Figure 7H), which we attributed to the dramatic increase in all-*trans*-RE #1 (peak c in Supplemental Figure 7A) levels (predicted [LS] mean difference = 925.4 pmol/eye; *P* < 0.0001 Supplemental Figure 7E). These results indicate that PR-localized LRAT can respond to transient increases in all-*trans*-retinal by sequestering its reduced derivative, all-*trans*-retinol, as all-*trans*-REs.

An analogous diminution of the *cis*:*trans* RE ratio was observed in PR-*Lrat^–/–^* (WT) animals, but to a lesser degree (predicted [LS] mean difference = 1.404; *P* = 0.0026), raising a concern that we might not be able to distinguish changes in retinoid isomeric composition in the RPE versus PRs after bleaching. To address this ambiguity, we performed the same bleaching experiments on DA-unbleached and 3-day-bleached WT and PR-*Lrat^+/–^* animals, but instead of processing whole eyes for retinoids, we dissected the neural retina from the underlying RPE/choroid under dim red light and processed the dissected tissues separately to eliminate RPE-localized changes in RE levels (Supplemental Figure 8). In this case, we found no REs in either the unbleached or bleached WT mice, whereas we observed significant increases in 11-*cis*-RE #1 (peak a in Supplemental Figure 8A; predicted [LS] mean difference = 162.9 pmol/eye; *P* = 0.0213) and all-*trans*-RE #1 (peak c in Supplemental Figure 8A; predicted [LS] mean difference = 346.4 pmol/eye; *P* < 0.0001) in the bleached PR-*Lrat^+/–^* mice relative to the unbleached controls (Supplemental Figure 8, E–G); 11-*cis*-RE #2 levels (bleached vs. unbleached) did not significantly differ (Supplemental Figure 8F, *P* > 0.05). The ratio of 11-*cis*- to all-*trans-*retinoids in the neural retina samples showed a unique and significant decrease within the bleached PR-*Lrat^+/–^* cohort relative to the unbleached cohort (predicted [LS] mean difference = 1.630; *P* = 0.0286) but not in the bleached versus unbleached WT cohorts (Supplemental Figure 8K; *P* = 0.124). These results confirm a neural retina-specific increase in all-*trans*-RE #1 levels within bleached PR-*Lrat^+/–^* mice relative to unbleached PR-*Lrat^+/–^* controls, which significantly altered the ratio of 11-*cis-* to all-*trans-*retinoids, indicating that PR-localized LRAT is not only responsible for trapping 11-*cis*-retinol sourced from the RPE, but also for sequestering all-*trans*-retinol after bleaching of rhodopsin. Our electrophysiological, visual cycle kinetics, and successive bleaching experiments in PR-*Lrat^+^* mice indicate that the REs sequestered by PR-localized LRAT do not reenter the visual cycle and do not accelerate visual chromophore regeneration.

### In situ visualization of PR-localized RE species using TP imaging.

**Figure 3:**
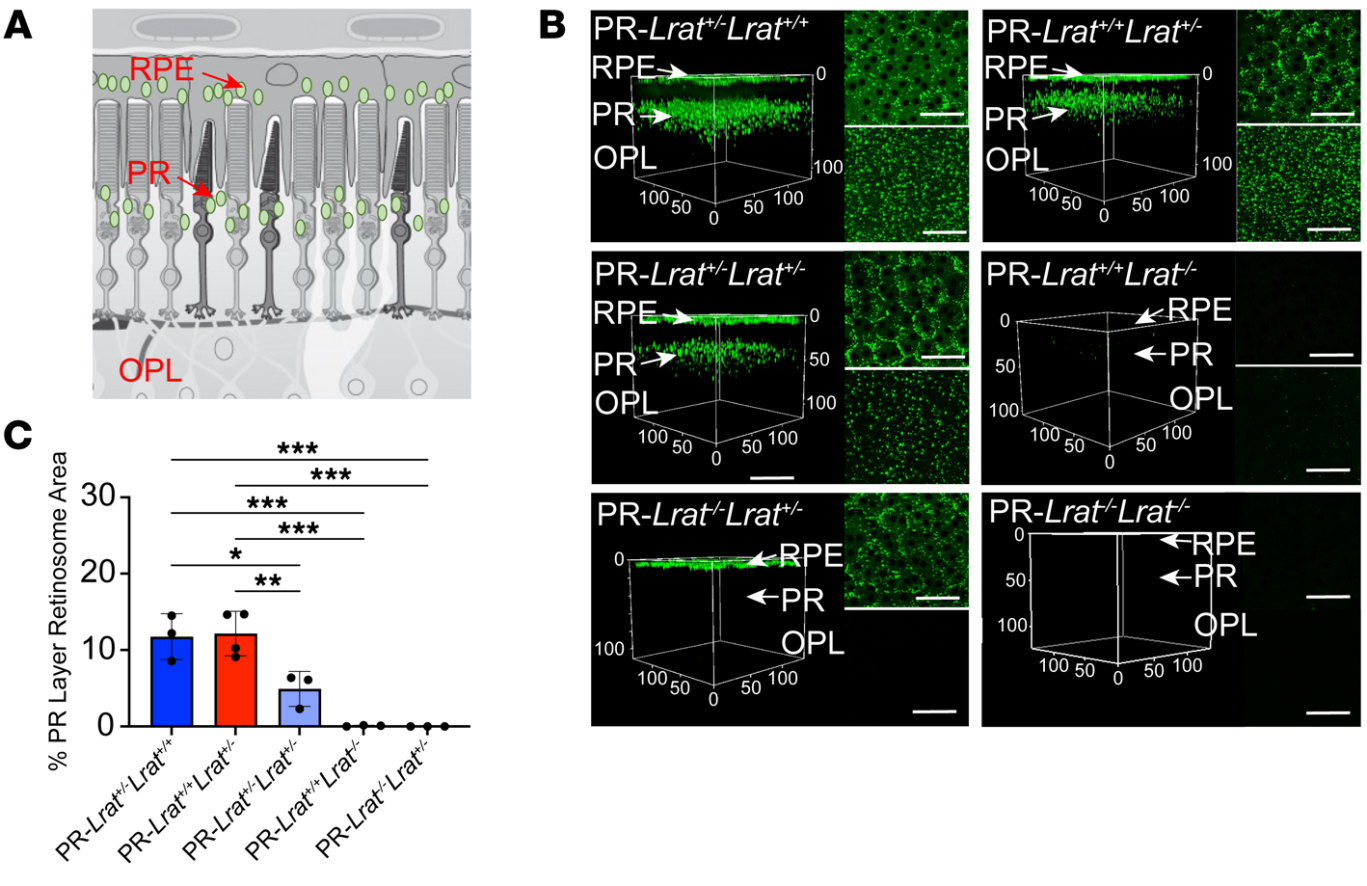
RE structures in mice expressing LRAT under the *Gcap1* promoter. (**A**) Cross-sectional diagram of the outer retina indicating the RPE, PR-IS (labeled as PR), and outer plexiform layer (OPL). Retinosome-like structures are represented by green oval shapes. (**B**) TP imaging of intact mouse eyes. Images show examples of 3D retinal visualizations, assembled from 56 frames and acquired every 2 μm along the retinal thickness. The RPE is at z = 0 μm. The top right inset images display RPE en face, and the bottom right inset images display the maximum projection through the PRs. Axes are labeled in μm; inset images correspond to the same region as the 3D reconstructions. Scale bars: 50 μm. (**C**) Calculated area of PR retinosome-like structures as a function of genotype, quantified in maximum projection images, starting 14 μm beneath the RPE and culminating 110 μm beneath the RPE. Data are shown as the mean ± SD. Significant results are provided, *P ≤ 0.05; **P ≤ 0.01; ***P ≤ 0.001; ****P ≤ 0.0001, Tukey’s multiple comparisons tests post 1-way ANOVA.

To assess localization of REs in situ and confirm cell-specific localization of RE products, we performed TP imaging on intact eyes from albino PR-*Lrat^+^* mice alongside genetic controls (Figure 3 and Supplemental Videos 1–4). PR-*Lrat^+^* mice with at least one WT *Lrat* allele (*Lrat^+/+^* or *Lrat^+/–^*) had RE-rich retinosomes in the RPE layer and an additional, unique layer of retinosome-like structures present in the PR layer (Figure 3, A and B). These retinosome-like structures were not present in the WT controls, suggesting that the TP scan was detecting REs generated by PR-localized LRAT in PR-*Lrat^+^* mice. In contrast, only minimal retinosome signal was detected in the PRs of PR-*Lrat^+/+^Lrat^–/–^* mice, suggesting that while PR-LRAT protein was still expressed (Figure 2), it had minimal access to retinol substrates to synthesize REs in the absence of an intact visual cycle (i.e., lack of natural LRAT in the RPE). Comparing the area of retinosome-like structures across each genotype, we found that they were densest in the PRs of PR-*Lrat^+/–^Lrat^+/+^* and PR-*Lrat^+/+^Lrat^+/–^* mice, less dense in the PRs of PR-*Lrat^+/–^Lrat^+/–^* mice, highly diminished in the PRs of PR-*Lrat^+/+^Lrat^–/–^* mice, and undetectable in the PRs of PR-*Lrat^–/–^Lrat^+/–^* mice (Figure 3C). No retinoids were observed in the neural retinas or RPE of PR-*Lrat^–/–^Lrat^–/–^* controls.

Next, we compared the phasor signature (corresponding to fluorescence lifetime) of the retinosome-like structures found in the PR-IS of the PR-*Lrat^+^* mice to that of the retinosomes normally found within WT [B6(Cg)-*Tyr^c-2J^*/J] and *Rpe65^–/–^* mice (Supplemental Figure 9) (28). These phasor signatures are unique and easily distinguishable, facilitating identification of different retinoid species in situ (29). We observed that the retinosome-like structures found in the PR layer of PR-*Lrat^+/–^Lrat^+/–^* mice occupied the same area of the phasor plot as the retinosomes found in the RPE of WT and *Rpe65^–/–^* mice (Supplemental Figure 9A). We compared phasor data from the retinoids in PRs of litter-matched PR-*Lrat^–/–^Lrat^+/–^* (WT) negative control mice, the retinosome-like structures of PR-*Lrat^+^* mice, and the phasors of endogenous PR retinoids of WT mice. As expected, the compounds present in the PRs of the PR-*Lrat^–/–^Lrat^+/–^* negative control mice did not have a phasor signature resembling the retinosome-like structures found in PR-*Lrat^+^* mice and in native RPE retinosomes. This distinction was evident when we compared mean phasor time values from the RPE of WT and *Rpe65^–/–^* mice to those of the PR-*Lrat^+/–^Lrat^+/–^* and PR-*Lrat^–/–^Lrat^+/–^* PRs (Supplemental Figure 9B). Superimposition of the fluorescence excitation spectra demonstrated that the spectrum for RPE retinosomes of WT mice was identical to that of the retinosome-like structures in the PRs of PR-*Lrat^+/–^Lrat^+/–^* mice (Supplemental Figure 9C). TP-based retinoid spectra from intact mouse eyes, however, do not provide the resolution necessary to distinguish between 11-*cis*-RE and all-*trans*-RE isoforms, as their excitation-emission characteristics are similar. As *Gcap1* displays higher levels of expression in cones compared with rods (30), we tested whether differential expression of LRAT driven by the Gcap1 promoter would manifest as preferential localization of retinosome-like structures in cones. We found that the retinosome-like structures were evenly distributed across the PRs and were not selectively produced in cones (Supplemental Figure 9D).

### Effects of genetic perturbations in retinoid-handling genes on retinoid distribution.

**Figure 4:**
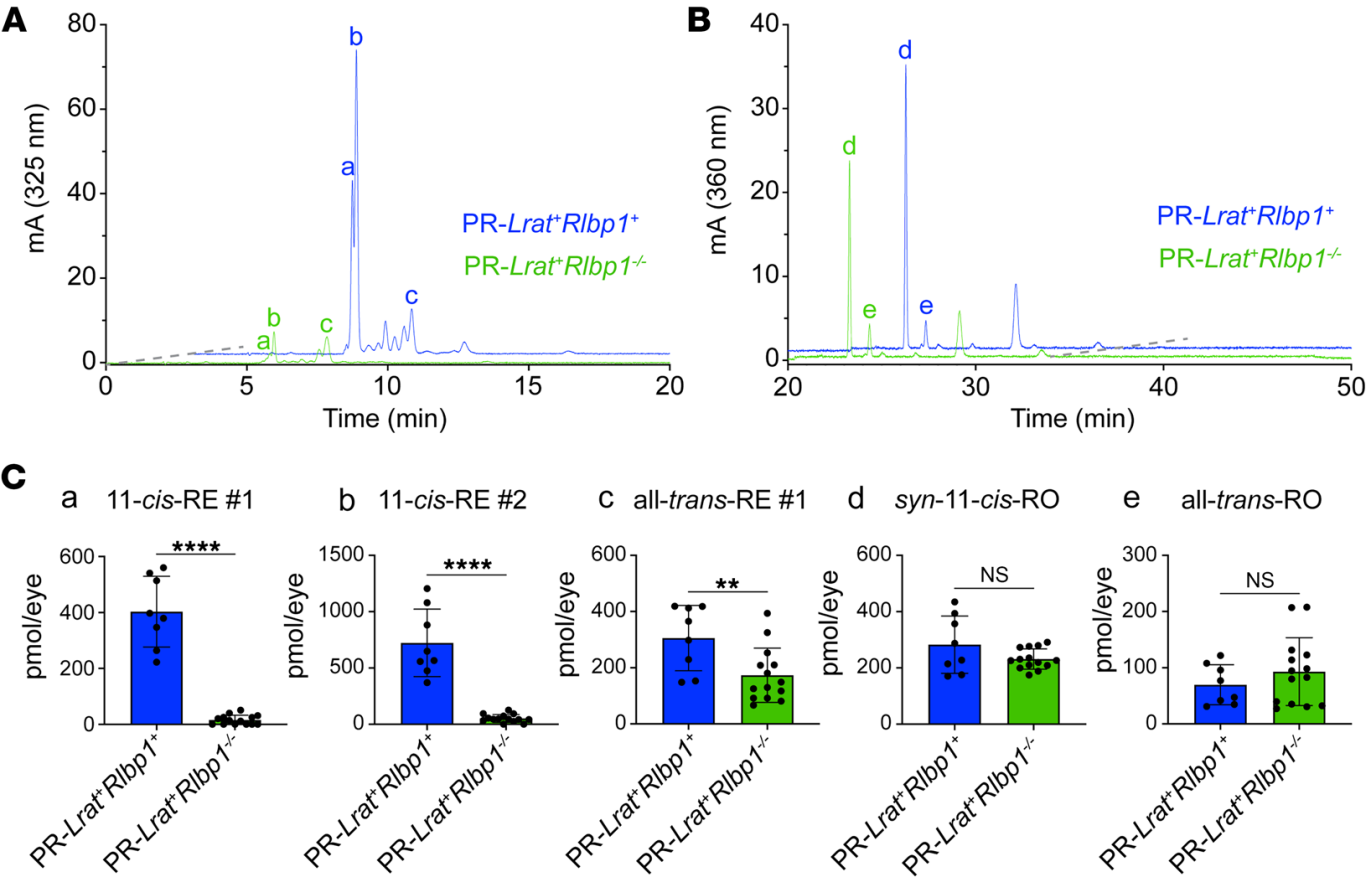
Dependence of retinyl-ester accumulation on CRALBP in PR-*Lrat^+^* mice. (**A** and **B**) Representative baseline HPLC traces for aliquots of whole-eye retinoid extracts from 6- to 8-week-old DA PR-*Lrat^+^Rlbp1^+^* mice (pooled heterozygote and homozygote animals; blue trace) and PR-*Lrat^+^Rlbp1^–/–^* mice (green trace), with a focus on the RE-elution time frame (**A**) and RO-elution time frame (**B**). The 2 predominant, earlier-eluting 11-*cis*-RE peaks are labeled a and b; the later-eluting, predominant all-*trans*-RE peak is labeled c. Traces are offset for ease of comparison between genotypes; dashed gray lines indicate angle of offset. (**C**) Quantification of the content of (a) 11-*cis*-RE #1, (b) 11-*cis*-RE #2, (c) all-*trans*-RE #1, (d) *syn*-11-*cis*-RO, and (e) all-*trans*-RO in whole-eye retinoid extracts from DA, 6- to 8-week-old mice (PR-*Lrat^+^Rlbp1^+^*, *n* = 8; PR-*Lrat^+^Rlbp1^–/–^*, *n* = 14). Results of unpaired *t* tests are provided above each plot. *P ≤ 0.05; **P ≤ 0.01; ***P ≤ 0.001; ****P ≤ 0.0001. Data are shown as the mean ± SD.

After confirming the consistency of our LRAT monitoring system in PR-*Lrat^+^* mice, we then sought to cross our PR-LRAT mice with other mouse models that have disruptions of retinoid-handling genes. We hypothesized that we would be able to observe cell-type resolved disruptions in retinoid trafficking and isomeric composition if we used our monitoring system in mice lacking 11-*cis* retinoid shuttling in the RPE and MG (*Rlpb1^–/–^* mice), in mice lacking a visual cycle (*Lrat^–/–^* mice), or mice lacking PR-localized RDHs linked to human retinopathy (*Rdh12^–/–^* mice). First, we crossed PR-*Lrat^+^* mice with *Rlpb1^–/–^* mice, which have a global knockout of the *Rlbp1* gene, a carrier protein implicated in accelerating dark adaptation and serving as a selective, intracellular reservoir of 11-*cis* retinoids in both the MG and RPE (15, 31). Comparing whole-eye retinoid extracts from the PR-*Lrat^+^Rlbp1^+^* (pooled heterozygote and homozygote PR-*Lrat^+^Rlbp1^+^* animals) and PR-*Lrat^+^Rlbp1^–/–^* mice, we found a large decrease in the amount of 11-*cis*-RE synthesis in the PR-*Lrat^+^Rlbp1^–/–^* mice relative to the PR-*Lrat^+^Rlbp1^+^* mice (–387.5 ± 33.69 pmol/eye for 11-*cis*-RE #1, peak a in Figure 4A; *P* < 0.0001; –671.3 ± 79.59 pmol/eye for 11-*cis*-RE #2, peak b in Figure 4A; *P* < 0.0001; Figure 4). There was a much smaller decrease in the level of all-*trans*-RE #1 (peak c in Figure 4A) synthesis (–132.3 ± 46.06 pmol/eye; *P* = 0.0094) in the PR-*Lrat^+^Rlbp1^–/–^* mice, and no significant difference in the levels of *syn*-11-*cis*-RO or all-*trans*-RO, (Figure 4, B and C) suggesting that there was a selective decrease in 11-*cis*-RE synthesis by PR-LRAT in the absence of *Rlbp1*.

**Figure 5:**
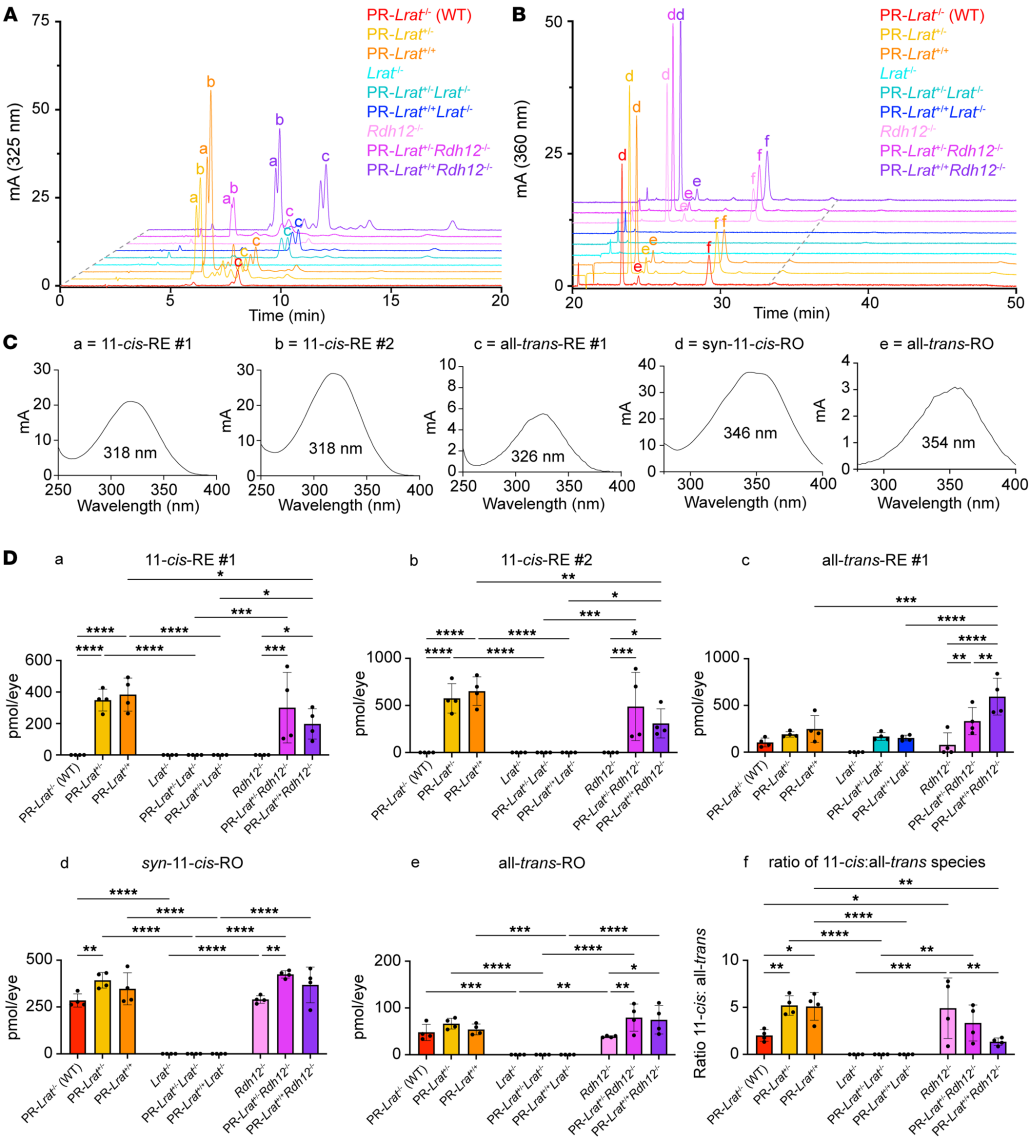
Baseline profiling of retinoids isolated from 6- to 7-week-old dark-adapted animals. (**A**) Representative HPLC traces of RE-elution patterns for aliquots of whole-eye retinoid extracts from 6- to 7-week-old DA mice. Individual RE peaks are labeled a–c: 11-*cis*-RE #1 (a); 11-*cis*-RE #2 (b); and all-*trans*-RE #1 (c). Quantification of each peak is shown in **D** (dashed gray lines indicate angle of offset). (**B**) Representative HPLC traces of RO-elution patterns for each genotype. Individual RO peaks are labeled d–f: *syn*-11-*cis*-RO (d); all-*trans*-RO (e); and *anti*-11-*cis*-RO (f). Quantification of *syn*-11-*cis*-RO and all-*trans*-RO is shown in **D** (dashed gray lines indicate angle of offset). (**C**) Representative absorption spectra for 11-*cis*-RE #1, 11-*cis*-RE #2, all-*trans*-RE #1, *syn*-11-*cis*-RO, and all-*trans*-RO. λ_max_ values are provided beneath each peak. (**D**) Quantification of 11-*cis*-RE #1, 11-*cis*-RE #2, all-*trans*-RE #1, *syn*-11-*cis*-RO, and all-*trans*-RO for each genotype (*n* = 4). A ratio of the sum of 11-*cis* retinoids quantified and the sum of all-*trans* retinoids quantified is provided. Results of Tukey’s multiple comparisons tests performed after 2-way ANOVA are provided. *P ≤ 0.05; **P ≤ 0.01; ***P ≤ 0.001; ****P ≤ 0.0001. Data are shown as the mean ± SD.

We then assessed the retinoid content of PR-*Lrat^+^Lrat^–/–^* mice and *Lrat^–/–^* controls, which lack an intact visual cycle and have no 11-*cis*-RO present in PRs (Figure 5). Retinoid extracts from PR-*Lrat^+^Lrat^–/–^* mice lacked 11-*cis*-REs, retinal oximes, or retinols but consistently had 2 all-*trans*-RE peaks. The litter-matched *Lrat^–/–^* controls, however, had no detectable levels of all-*trans*-REs or any other retinoid species, consistent with previous phenotyping of *Lrat^–/–^* mice (24). PR-IS–localized RDH12 has a role in catalyzing the reduction of IS-localized retinals to retinols to limit the buildup of toxic retinal byproducts (32). Therefore, we crossed the PR-*Lrat^+^* mice with a global-*Rdh12^–/–^* line. We found that PR-*Lrat^+^Rdh12^–/–^* mice exhibited 11-*cis*-RE accumulation as in the PR-LRAT knockin mice, but there was a significant decrease in 11-*cis*-RE accumulation with PR-*Lrat^+/+^Rdh12^–/–^* mice versus PR-*Lrat^+/+^* mice, with a mean difference of –185.9 pmol/eye for 11-*cis*-RE #1 (peak a in Figure 5A; *P_adj_* =0.0207) and a mean difference of –342.0 pmol/eye for 11-*cis*-RE #2 (peak b in Figure 5A, *P_adj_* = 0.0091). Conversely, there was an increase in all-*trans*-RE #1 levels (peak c in Figure 5A) in the PR-*Lrat^+/+^Rdh12^–/–^* mice relative to PR-*Lrat^+/+^* mice (mean difference = 347.2 pmol/eye; *P_adj_* = 0.0003). The all-*trans*-RE #1 levels in the PR-*Lrat^+/+^Rdh12^–/–^* mice were significantly higher than in PR-*Lrat^+/–^Rdh12^–/–^* mice (mean difference = 262.8 pmol/eye; *P_adj_* = 0.0048) and *Rdh12^–/–^* mice (mean difference = 516.7 pmol/eye; *P_adj_* < 0.0001). The all-*trans*-RE #1 levels in PR-*Lrat^+/–^Rdh12^–/–^* mice were also significantly higher than those of the *Rdh12^–/–^* mice (mean difference = 253.9 pmol/eye; *P_adj_* = 0.0064). There were no statistically significant changes in *syn*-11-*cis*-RO (peak d in Figure 5B) levels between the PR-*Lrat^+^* single knockin mice and PR-*Lrat^+^Rdh12^–/–^* mice (Figure 5D; accompanying retinoid spectra included in Figure 5C), though there was an increase in all-*trans*-RO (peak e in Figure 5B) in PR-*Lrat^+^Rdh12^–/–^* mice relative to the *Rdh12^–/–^* mice (Figure 5D). The PR-*Lrat^+/+^Rdh12^–/–^* mice had a significantly decreased ratio of 11-*cis* to all-*trans* retinoids relative to the *Rdh12^–/–^* mice (mean difference = –3.573; *P_adj_* = 0.0036) and PR-*Lrat^+/+^* mice (mean difference = –3.756; *P_adj_* = 0.0022). There was also a significant increase in the ratio of summed 11-*cis*: all-*trans* retinoids in the *Rdh12^–/–^* mice relative to the WT controls (mean difference = 2.90; *P_adj_* = 0.0188), which we attribute to decreased levels of baseline all-*trans*-RE #1 accumulation in *Rdh12^–/–^* mice. There was an increase in the ratio of 11-*cis-* to all-*trans*-retinoids in the PR-*Lrat^+/–^* (*P_adj_* = 0.0094) and PR-*Lrat^+/+^* (*P_adj_* = 0.0122) mice relative to the WT controls. In short, introduction of PR-*Lrat* into *Rlbp1*-knockout and *Lrat*-knockout animals revealed that PR-localized REs accumulate in the IS in a visual cycle– and CRALBP-dependent manner, whereas elimination of *Rdh12* appears to increase the ratio of all-*trans*-RE to 11-*cis*-RE synthesis in the PR-IS due to lingering all-*trans*-retinal before it is reduced and trapped in PR retinosomes (Figure 5D, panel f).

### Effects of ectopic LRAT expression in MG and RGCs.

**Figure 6:**
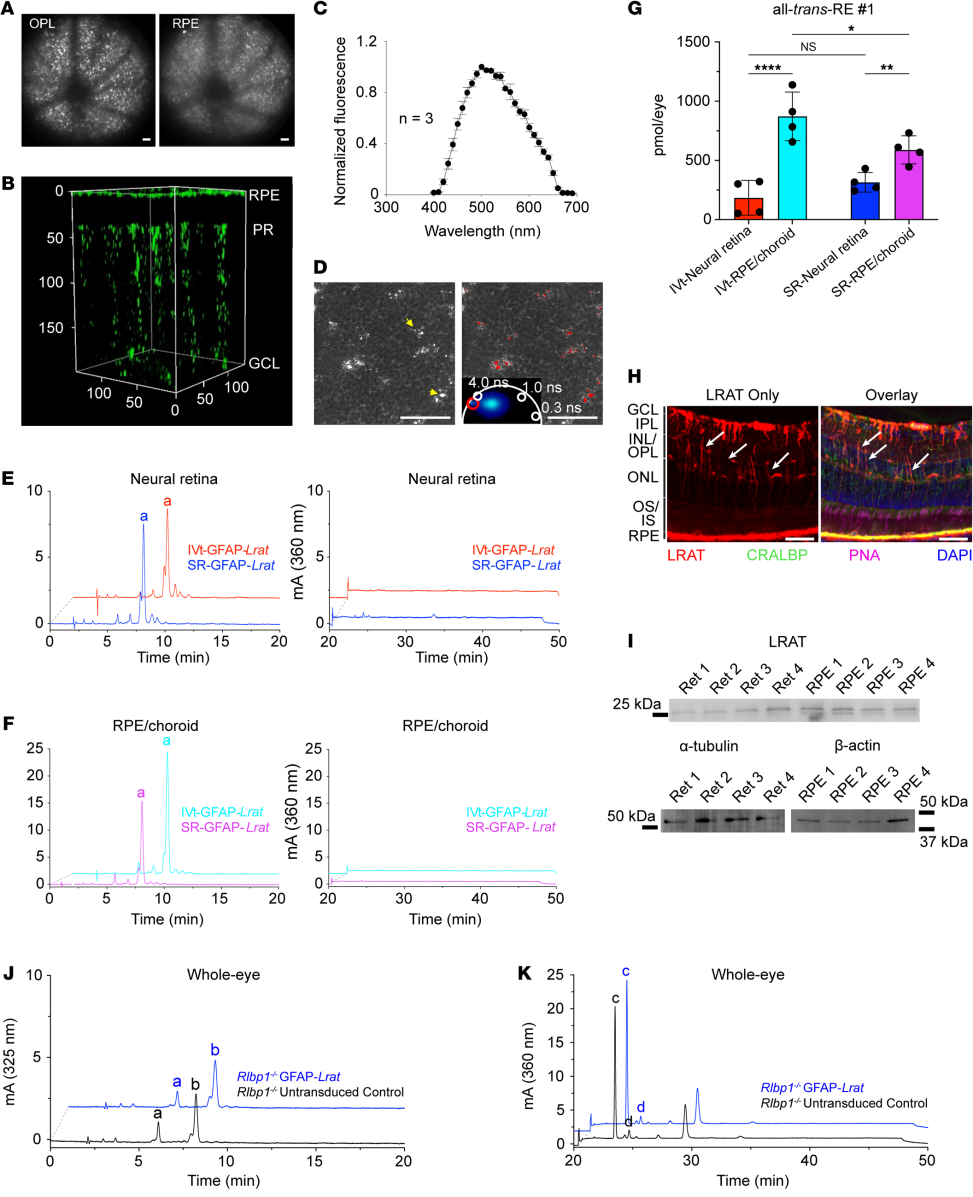
Retinosome-like structures within MGs after intravitreal and subretinal injections with AAV-7m8-GFAP-*Lrat*. (**A**) TP excitation images of a WT mouse after 3 weeks intravitreal (IVt) injection (scale bar: 50 μm). (**B**) 3D visualizations from TP en face ex vivo eyes (2 μm along the retinal thickness; RPE at *z* = 0 μm; axes labeled in μm). (**C**) Fluorescence emission spectra, ex vivo eyes at the ONL (*n* = 3, error bars indicate SD). (**D**) Left: TP-fluorescence image at the ONL; yellow arrows point to round spots with 0.8 μm–1.5 μm diameters. Right: Red-colored image pixels correspond to phasor points encircled in red in the phasor plot in the inset (scale bar: 50 μm). (**E**) HPLC retinoid traces of neural retina extracts from subretinal (SR) and IVt-injected WT mice. RE- and RO-elution time frames are shown on the left and right, respectively. All-*trans*-RE #1 peaks are marked as a (*n* = 4). (**F**) Retinoid traces from RPE/choroid extracts from WT mice after TP imaging. (**G**) Comparison of all-*trans*-RE #1 content in neural retinal extracts and RPE/choroid extracts from mice. Results of Tukey’s multiple comparisons tests after 2-way ANOVA. *P ≤ 0.05; **P ≤ 0.01; ***P ≤ 0.001; ****P ≤ 0.0001. *n* = 4. (**H**) IF staining of WT retinal cryosection 3 weeks after IVt injection. White arrows indicate regions with LRAT signal spanning MG cell bodies (*n* = 4; scale bar = 50 μm). (**I**) Immunoblotting of dissected eye from WT animals 3 weeks after IVt injection (*n* = 4). (**J** and **K**) Retinoid traces from DA untransduced *Rlbp1^–/–^* control eyes 3 weeks after injection with AAV-7m8-GFAP-*Lrat*. For **J** and **K**, 11-*cis*- and all-*trans*-RE are labeled a and b, respectively; *syn*-11-*cis*-RO and all-*trans*-RO are labeled c and d, respectively (*n* = 2 for *Rlbp1^–/–^* control, *n* = 4 for *Rlbp1^–/–^* AAV-7m8-GFAP-*Lrat*). Full datasets are included in Supplemental Figures 10 and 11.

We then wanted to determine if our LRAT monitoring system could detect 11-*cis*-retinol byproducts in MG, which would support putative alternative visual chromophore regeneration in MG. To test this, we designed an AAV-7m8-GFAP-*Lrat* construct for targeted transduction of murine MG. Three weeks after subretinal (SR) or intravitreal (IVt) injection of albino WT [B6(Cg)-*Tyr^c-2J^*/J] mice, we found a substantial accumulation of retinosome-like, autofluorescent punctae within the neural retinas of live mice, using 740 nm TP excitation (Figure 6A). Using 3D volumetric reconstructions of TP images in ex vivo eyes, we found that the autofluorescent spots extended from the RGC layer (GCL) to the PR-IS, which aligns with the characteristic span of MG cell bodies within the neural retina (Figure 6B) (33). Collected spectra aligned almost identically with those from the PR-localized retinosome-like structures using the same TP method (Figure 6C). Examination of these structures revealed that they ranged from 0.8 to 1.5 μm in diameter (Figure 6D). These autofluorescent punctae appeared consistently using TP excitation in mice transduced with AAV-7m8-GFAP-*Lrat*, regardless of whether the mice were injected via SR or IVt routes (Supplemental Figure 10, A and B). Differences in the phasor signatures could be attributable to a difference in lipid composition. These structures contained all-*trans*-RE peaks in each of the transduced mice (Figure 6, E and F, and Supplemental Figure 10, C and D), with no marked differences between the 2 injection groups (Figure 6G). We stained cryosections of IVt-injected AAV-7m8-GFAP-*Lrat* animals with anti-LRAT and anti-CRALBP antibodies coupled with fluorophore-conjugated PNA and DAPI, identifying regions of LRAT staining in the inner retina stretching down to the PR ONL (Figure 6H). These cell bodies, which spanned several layers of the neural retina, were positive for CRALBP, indicating that MG were transduced with AAV-7m8-GFAP-*Lrat*. The localization of the LRAT signal in the transduced animals differed considerably from that of PR-LRAT, as visualized using confocal microscopy (Supplemental Figure 10E). We confirmed expression of LRAT protein in the neural retina homogenates of the AAV-7m8-GFAP-*Lrat*–transduced animals, using immunoblotting (Figure 6I).

We then applied our same rationale of determining whether MG-localized LRAT could detect transient shifts in local retinol isomeric composition, predicting that an increase in all-*trans*-REs would provide support for RGR activity and alternative visual chromophore regeneration occurring in MG. As such, we examined whether 3 days of consecutive bleaching followed by DA had any effect on the isomeric composition of REs accumulating in the MG of WT mice transduced with AAV-7m8-GFAP-*Lrat* and observed no appreciable effects on RE isomeric composition or rate of RE accumulation within the MG (Supplemental Figure 11, A–C). We noticed that following DA, in both the unbleached and 3-day-bleached animals transduced with AAV-7m8-GFAP-*Lrat*, a small, earlier-eluting RE peak appeared, which was spectrally attributable to an 11-*cis*-RE (peak a in Supplemental Figure 11A), followed by the predominant RE peak corresponding to an all-*trans*-RE (peak b in Supplemental Figure 11A). We transduced *Lrat* into the MG of the *Lrat^–/–^* animals, expecting RE accumulation to be eliminated from the MG but surprisingly found that there were still trace amounts of all-*trans*-RE #1 within dissected *Lrat^–/–^* neural retina extracts (Supplemental Figure 11, D and E). To examine whether ectopic LRAT-mediated accumulation of all-*trans*-REs in the MG was dependent on CRALBP pulling retinoids inwards from the RPE/PRs to the MG, we transduced *Rlbp1^–/–^* mice with AAV-7m8-GFAP-*Lrat* and processed them as described above. We found no difference in the amount of RE accumulation in whole-eye retinoid extracts from the LRAT-transduced versus untransduced *Rlbp1^–/–^* mice, leading us to conclude that CRALBP is necessary for any MG-localized RE accumulation (Figure 6, J and K, and Supplemental Figure 11, F and G).

**Figure 7:**
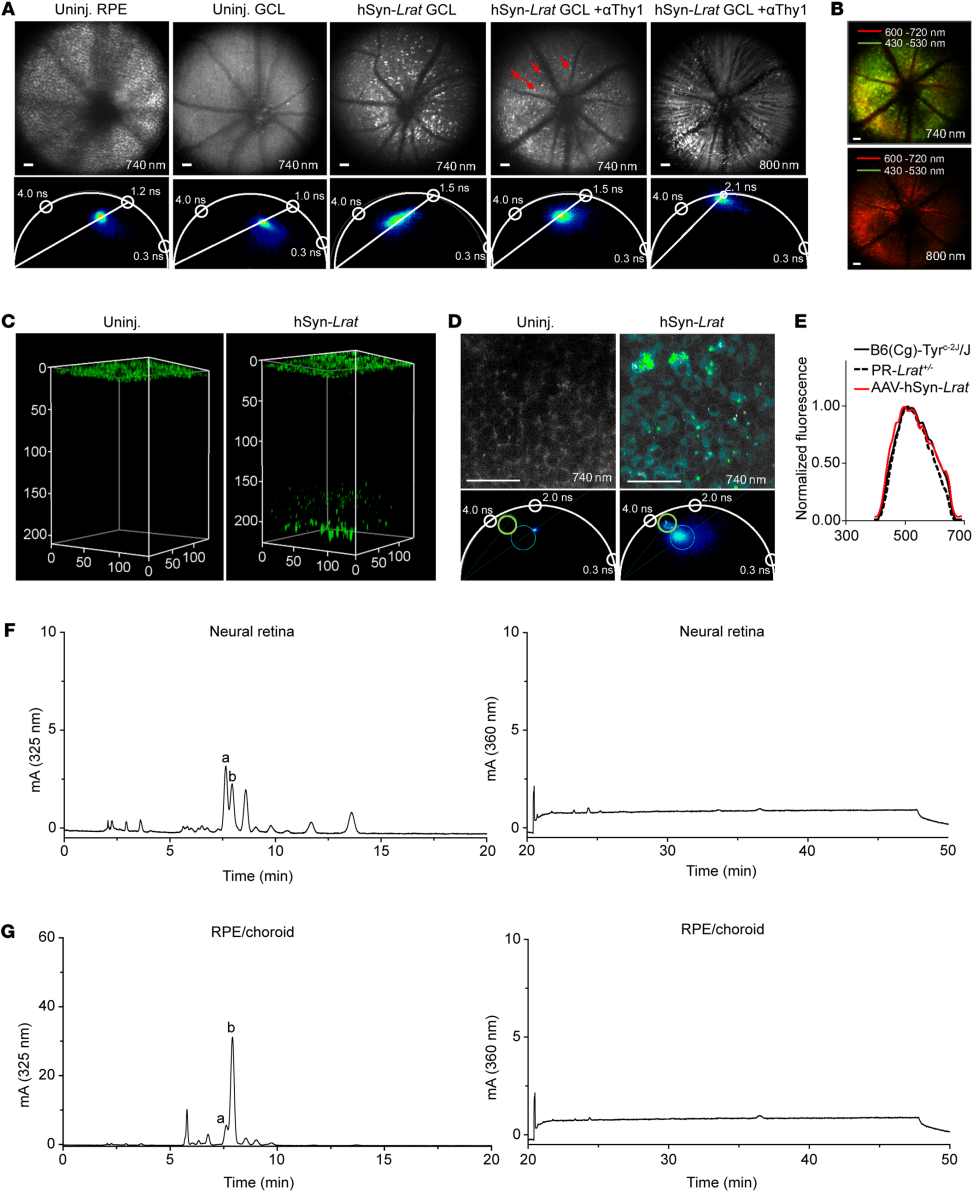
Retinosome-like structures in the mouse GCL after intravitreal injection with AAV2-hSyn-*Lrat*. (**A** and **B**) Data from TP-excitation imaging through the pupil in a live mouse. Excitation wavelengths are indicated in images. (**A**) Fluorescence intensity–based images (top row) and phasor plots (bottom row), including an uninjected (uninj.) WT eye on the RPE layer; an uninj. WT eye on the GCL; a WT eye 6 weeks after IVt injection with AAV2-hSyn-*Lrat* on the GCL; a WT eye 6 weeks after IVt injection with AAV2-hSyn-*Lrat,* supplemented with rhodamine-conjugated anti-Thy1.2 antibody. Phasor lifetimes indicated in phasor plots. Red arrows indicate bright granules. Scale bar: 50 μm. (**B**) Immunohistochemistry. Top (740 nm laser) and bottom (800 nm laser) images show the GCL layer 6 weeks after IVt injection with AAV2-hSyn-*Lrat* and 48 hours after IVt injection with αThy1-rhodamine. Detection pass bands: 430–530 nm for retinosome emission, and 600–720 nm for rhodamine emission. Scale bar: 50 µm. (**C**) 3D-volume visualizations assembled from en face fluorescence intensity–based images acquired every 2 μm along the retinal thickness in the ex vivo mouse eyes. Scales are given in μm; images are pseudo-colored in green. (**D**) Ex vivo en face FLIM of GCL in uninj. and injected mouse eyes. Phasor scales are provided in white. Scale bar: 50 μm. (**E**) TP fluorescence emission spectra from WT RPE, PR-*Lrat^+/–^Lrat^+/–^* PRs, and the GCL from AAV2-hSyn-*Lrat–*injected WT mice. (**F**) HPLC traces of retinoid extracts from the neural retinas of B6(Cg)-*Tyr^c-2J^*/J (WT) mice 6 weeks after IVt injection with AAV2-hSyn-*Lrat* and TP imaging (light-adapted, *n* = 4). (**G**) HPLC traces of retinoid extracts from the RPE/choroid of B6(Cg)-*Tyr^c-2J^*/J (WT) mice 6 weeks after IVt injection with AAV2-hSyn-*Lrat* and TP imaging (light-adapted, *n* = 4). For **F** and **G**, all-*trans*-RE peaks are labeled a and b. Left traces are focused on the RE-elution and the right traces on the RO-elution. Full sets of retinoid traces in **F** and **G** are featured in Supplemental Figure 13, A and B.

Given the predominance of all-*trans*-RE isoforms in LRAT-transduced MG, we wanted to see if this same observation of all-*trans*- rather than 11-*cis*-RE accumulation occurred in RGCs, which would indicate restricted 11-*cis*-retinol delivery from MG to RGCs. After confirming that transduction of RGCs with AAV2-hSyn-GFP displayed GCL localization of the GFP signal, we designed an AAV2-hSyn-*Lrat* construct for selective, IVt transduction of RGCs (34, 35) to assess whether there were any retinol species in the RGCs (Supplemental Figure 12). After IVt injection of albino WT [B6(Cg)-*Tyr^c-2J^*/J] mice with AAV2-hSyn-*Lrat*, we observed autofluorescent, retinosome-like structures localized to the GCL by 6 weeks after transduction using 740 nm TP excitation. To validate GCL localization, we injected a fluorophore-conjugated anti-Thy1 antibody IVt 48 hours before TP imaging and observed colocalization of the antibody signal with the autofluorescence punctae (Figure 7, A and B). 3D-volumetric reconstructions localized these autofluorescent, retinosome-like structures to the GCL (Figure 7, C and D). The spectrum of the retinosome-like structures in the RGCs was nearly identical to that of RPE-localized retinosomes in WT albino mice (Figure 7E). After TP imaging, we collected the light-adapted eyes and dissected the neural retina and RPE/choroid for retinoid analysis and observed an accumulation of all-*trans*-REs in the neural retina extracts (peaks a and b in Figure 7, F and G, and Supplemental Figure 13, A and B). These peaks were present in all of the transduced animals that we sampled. The elution pattern and ratios of the all-*trans*-RE peaks in the transduced RGCs differed from those of the PR-localized and MG-localized RE peaks. We then tested for the effects of light exposure/visual cycle progression. We saw that an earlier-eluting RE species within the neural retina extracts increased in concentration relative to the later-eluting all-*trans*-RE species with placement of the RGC-LRAT–transduced WT animals in the dark for the 6-week postinjection period (Supplemental Figure 13, C and D). After examining the spectrum of this earlier-eluting peak, we identified it as an 11-*cis*-RE (peak a in Supplemental Figure 13C). Thus, we determined that MG- and RGC-localized LRAT results in distinct patterns of RE synthesis and formation of retinosome-like structures.

### Analysis of LRAT transcript in single-cell/single-nuclei RNA-sequencing datasets for human retina and mature human retinal organoids.

**Figure 8:**
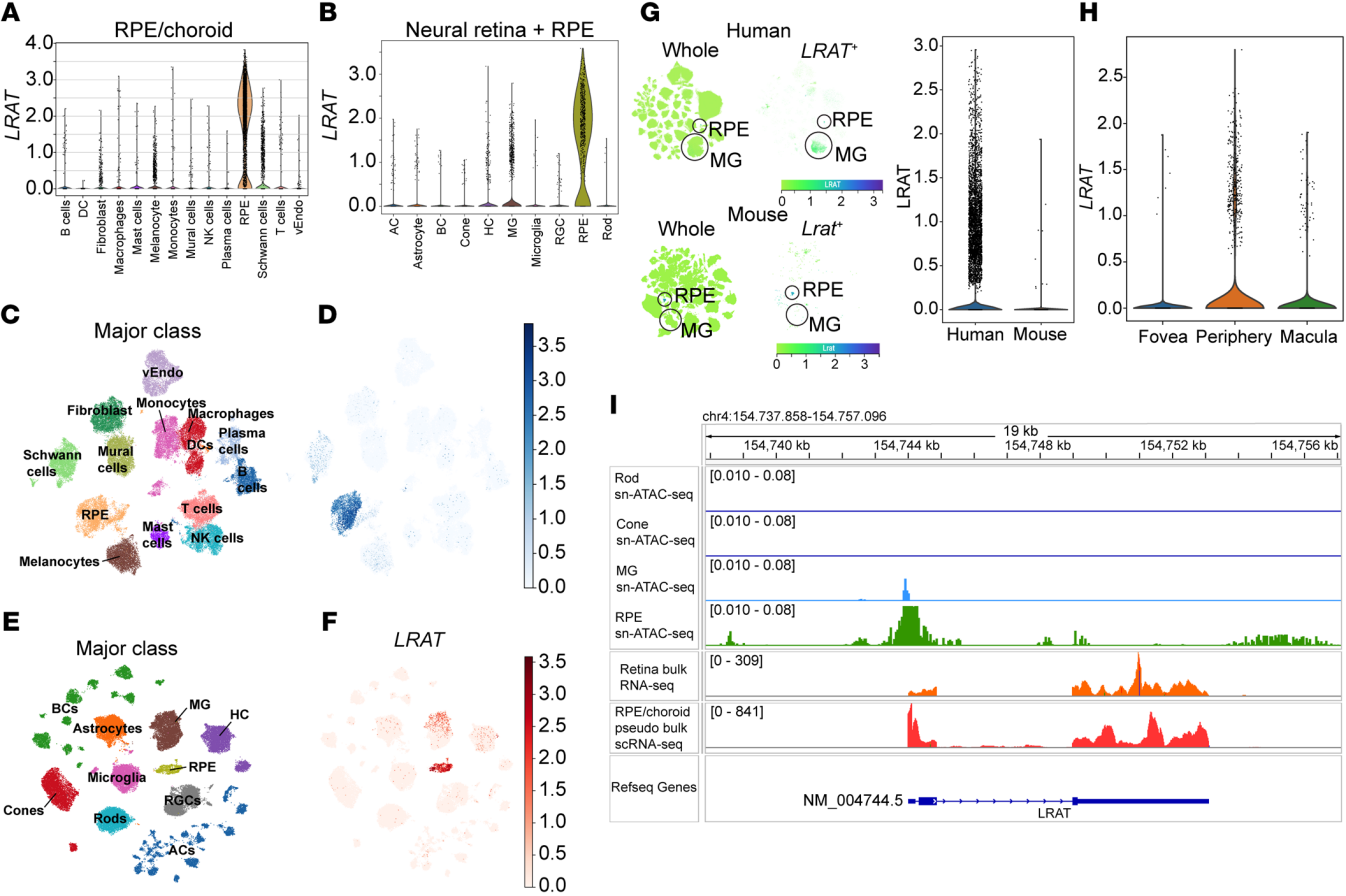
Expression of *LRAT* in the cell types of adult human RPE/choroid and retina. LRAT gene expression across cell types in adult human RPE/choroid (**A**) and neural retina/ RPE (**B**) based on sn-/sc-rNA-seq datasets. UMAP plots show the cell cluster identities (**C**) and *LRAT* expression (**D**) in adult human RPE/choroid; and the cell cluster identities (**E**) and *LRAT* expression (**F**) in adult human RPE/retina. (**G**) UMAP plots comparing LRAT^+^ cells from human retina sn-rNA-seq and mouse retina sc-rNA-seq datasets and an accompanying violin plot comparing LRAT gene expression between human and mouse MG from retinal sc-rNA-seq datasets. (**H**) Violin plot of *LRAT* gene expression across human MG collected from the fovea, peripheral, and macular areas of the retina, downsampled from the human CellxGene sn-rNA-seq atlas (36). (**I**) Genome tracks display bulk RNA-seq data from adult human retina, pseudo-bulk sc-rNA-seq data from adult human RPE/choroid, and sn-ATAC-seq data from adult human retina for rod cells (Rod), cone cells (Cone), MG, and RPE, with the Refseq gene NM_004744.5 LRAT spliceoform.

Owing to our observation of LRAT activity in transduced murine MG, we investigated whether there were any noncanonical LRAT transcripts within human and murine neural retina cell types involved at a low level in retinoid storage or visual chromophore regeneration. To accomplish this, we analyzed single-nuclei RNA-sequencing (sn-RNA-seq) data from adult human retina and single-cell RNA-sequencing (sc-RNA-seq) data from adult human RPE and choroid tissues. *LRAT* showed the highest expression in RPE cells, with additional expression observed in MG within the neural retina (Figure 8, A–F, and Supplemental Data File 1). Significantly higher LRAT expression was found in human MG versus mouse MG (Mann-Whitney *U* test, *P* = 5.09 × 10^–122^, Figure 8G and Supplemental Data File 2). UMAPs of LRAT^+^ MG from the human sn-RNA-seq and mouse sc-RNA-seq atlases reflected this difference (Figure 8G) regardless of donor age (Supplemental Data File 3). Notably, when we partitioned the MG from the human sn-RNA-seq atlas (36) based on retinal region, we found that there was a significant increase in *LRAT* expression in MG collected from the periphery compared with those collected from the foveal/macular regions (log fold change = 2.91, *P_adj_* = 2.24 × 10^–10^; Figure 8H, Wilcoxon’s rank-sum test, Supplemental Methods). Bulk RNA-seq analysis of adult human retina and pseudo-bulk sc-rNA-seq of RPE/choroid revealed that the predominant *LRAT* spliceoform is shared between the two tissues and that it resembles the NM_004744.5 spliceoform of *LRAT* (Figure 8I and Supplemental Figure 14). Correspondingly, the region chr4:154743707-154744257 (hg38) was identified as the primary promoter of *LRAT* in these tissues, based on sn-ATAC-seq datasets from adult human retina and RPE/choroid. Consistent with expression patterns, this promoter region exhibited the highest accessibility in RPE cells, though it remains open in MG cells (Figure 8I). We identified putative cis-regulatory elements and transcription factor motifs driving LRAT expression within the RPE and MG, based on sn-ATAC-seq data (Supplemental Figure 14). Among these, 1 cis-regulatory element is predicted to be a strong enhancer of *LRAT*, with exclusive accessibility in RPE cells, which could explain the abundant *LRAT* transcription found within the RPE.

We also examined sc-RNA seq datasets of human embryonic stem cell–derived retinal organoids for the presence of *LRAT* transcript and found trace amounts of *LRAT* expression in annotated RPE, extracellular matrix–associated cells, and glial cells within D200 (200 days after seeding) organoids (Supplemental Figure 15, A and B). Comparing *LRAT* expression in D180 and D260 induced pluripotent stem cell–derived organoid sc-rNA-seq datasets, we found that *LRAT* expression was present in the D260 glial cell population but not within the D180 glial cell population. This observation of *LRAT* transcription in the more mature D260 glial cell population aligns with the differentiation time frame of MG and suggests that a subset of MG present in human retinal organoids could express *LRAT* (Supplemental Figure 15C).

We extracted total RNA from dissected human neural retina and RPE/choroid tissues obtained from a pair of human donor eyes, reverse-transcribed it into cDNA, and performed both RT-PCR and qPCR analyses for *LRAT* and *RPE65* to assess the ratio of *LRAT* to *RPE65* transcript levels, using *GAPDH* as an internal control. We found that the difference between GAPDH-normalized neural retina *LRAT* amplicon intensity and the corresponding *GAPDH*-normalized RPE/choroid *LRAT* amplicon intensity (difference in mean ± SEM = 0.8216 ± 0.0638) was lower than the corresponding difference of *GAPDH*-normalized *RPE65* amplicon intensities (difference in mean ± SEM = 1.064 ± 0.075), suggesting that some *LRAT* transcript present in the neural retina is not attributable to RPE-associated cross-contamination (Supplemental Figure 16, A–C). This pattern was consistent when we performed qPCR on the same cDNA templates for *LRAT*, *RPE65*, and *GAPDH*. The difference in mean *LRAT* ΔCq values between the neural retina and RPE/choroid samples (difference in mean ± SEM = 0.484 ± 0.093) was smaller than the difference in mean *RPE65* ΔCq values between the neural retina and RPE/ choroid samples (difference in mean ± SEM = 2.123 ± 0.240), which indicates that there is *LRAT* transcript present within the neural retinal samples that cannot be attributed to contaminating RPE-derived transcripts (Supplemental Figure 16, D and E).

### Retinoid analyses of dissected whole human neural retina, whole RPE/choroid, perimacular neural retina punches, and RPE/ choroid punches.

**Figure 9:**
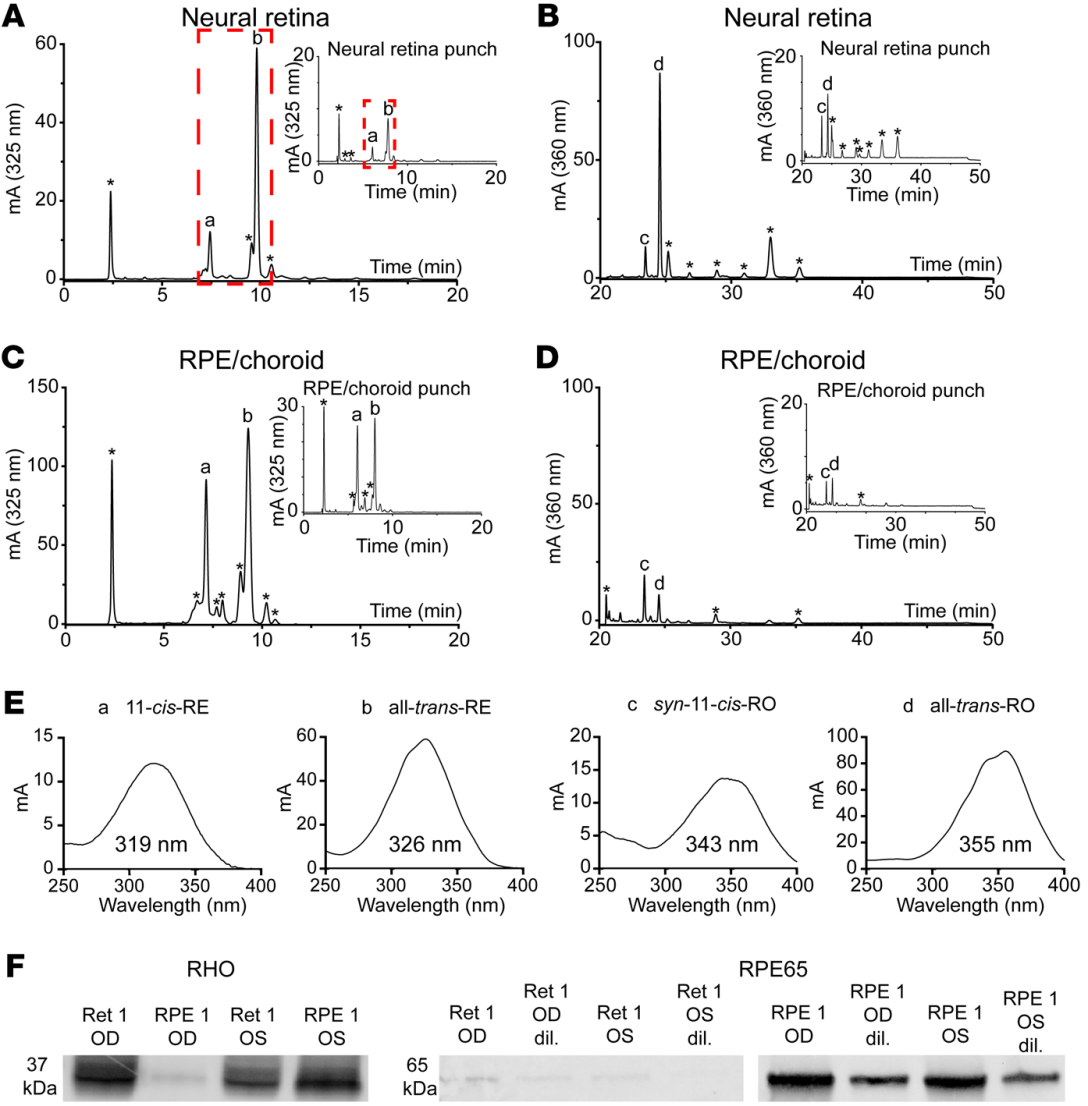
Retinoid analyses of dissected human neural retina and RPE/choroid tissue. (**A**) Representative HPLC trace of RE-elution extracted from dissected neural retina from a single human eye. Representative HPLC trace of REs extracted from a single human dissected neural retina. The top right inset is a representative HPLC trace of REs from 6 pooled, 6 mm perimacular punches from human neural retina. Red dashed boxes highlight a neural retina-localized 11-*cis*-RE (a) and an all-*trans*-RE (b). (**B**) Representative HPLC trace of ROs extracted from a single human dissected neural retina. The top right inset is a representative HPLC trace of ROs extracted from 6 pooled, 6 mm perimacular punches from human neural retina. (**C**) Representative HPLC trace of REs from a single human dissected RPE/choroid. The top right inset is a representative HPLC trace of REs extracted from 6 pooled, 6 mm perimacular punches from human RPE/choroid. (**D**) Representative HPLC trace of ROs extracted from a single human dissected RPE/choroid. The top right inset is a representative HPLC trace of ROs extracted from 6 pooled, 6 mm perimacular punches from human RPE/choroid. Peaks corresponding to 11-*cis*-RE (a), all-*trans*-RE (b), *syn*-11-*cis*-RO (c), and all-*trans*-RO (d) are labeled; peaks marked with an asterisk denote nonretinoid peaks, phase-shift artifacts, or retinoid peaks that are not central to our study. (**E**) Spectra of individual peaks corresponding to 11-*cis*-RE, all-*trans*-RE, *syn*-11-*cis*-RO, and all-*trans*-RO, from **A** and **B**. λ_max_ values are provided for each retinoid beneath each peak. (**F**) Immunoblots for rhodopsin (RHO) and RPE65 in 2 homogenates of whole human neural retina and RPE/choroid, taken from the right (OD) and left (OS) eyes of a single donor. RPE65 blotting was conducted on undiluted and 1:3.5-diluted (dil.) homogenates of neural retina (Ret) and RPE/choroid (RPE).

To search for RE products of MG-localized LRAT in human tissue, we extracted and analyzed retinoids present in human whole neural retina and pooled perimacular neural retina punches and compared them with retinoid extracts from the accompanying RPE/choroid material. We found that REs were present within both dissected whole neural retina and perimacular neural retina punches (Figure 9, A and E). All-*trans*-RE was the predominant peak (peak b in Figure 9, A and E) in the neural retinal extracts; 11-*cis*-RE was present in lower concentrations (peak a in Figure 9, A and E) and some samples lacked substantial 11-*cis*-RE peaks (see Supplemental Figure 17 for compiled traces). *Syn*-11-*cis*-RO (*syn*-11-*cis*-retinal derived) and photo-isomerized all-*trans*-RO (all-*trans*-retinal derived) were present within the neural retina samples, with some cross-contamination in the corresponding RPE/choroid samples (Figure 9, B and D, and Supplemental Figure 17). Robust RE peaks were present within the corresponding RPE/choroid samples (Figure 9C). Together, these results indicate the presence of 11-*cis*- and all-*trans*-REs within the neural retina whose ratio is distinct from that of the REs present in the RPE/choroid. We conducted anti-rhodopsin and anti-RPE65 immunoblots using dissected human neural retina and RPE/choroid material to assess the degree of cross-contamination of the dissected tissues (Figure 9F). Both of the human neural retina samples we analyzed had little RPE/choroid-derived RPE65 contamination. There was a substantial amount of rhodopsin contamination in one of our RPE/choroid samples, but this did not alter the conclusion that there was minimal RPE/choroid contamination in both of our neural retina samples, suggesting that the REs observed in the neural retinas were not contaminated by the RPE/choroid.

## Discussion

The visual system relies on the capture of light by pigments in the RPE, MGs, and selected RGCs (37, 38) (Figure 1 and Supplemental Figure 18). Not only do PR pigments and the photoisomerase RGR require renewal of active chromophore, but nonvisual opsins also depend on this process. These include OPN3 (expressed in retinal RGCs and MGs), OPN4 (melanopsin, expressed in intrinsically photosensitive RGCs), and OPN5 (expressed in RGCs) (39). The delivery of retinoids — the chromophores of these opsins — is essential for this function. The complexity of studying these processes arises from two main challenges. First, retinoid processing enzymes, binding proteins, and chromophore-accepting receptors are distributed throughout the retina (Figure 1 and Supplemental Figure 18). Second, retinoids are present at minute levels in the retina and RPE, apart from abundant, chromophore-bound opsins and the REs efficiently sequestered in retinosomes by LRAT (Figure 1). The results of this study with ectopically expressed LRAT highlight a tightly regulated equilibrium of retinoid species across various cellular compartments of the mammalian neural retina. We have monitored this equilibrium with unprecedented resolution using TP imaging combined with cell-selective expression or elimination of proteins involved in the visual cycle and retinoid trafficking. Our study reveals tight regulation of isomeric composition between cells of the neural retina, detecting a stark difference between the 11-*cis*-enriched PR-IS and the all-*trans*-enriched MG and RGC cellular compartments, with additional insights into how cell type–specific retinol levels and isomeric ratios change with perturbations to retinoid cycling and delivery. Since Wald’s discovery of the chemistry of vision based on retinoids, their isomerization, and redox processes (2), steady progress has been made in characterizing the molecular machinery involved in this process. Cumulatively, this progress should lead to improvements in designing approaches to treat retinal diseases.

### Expression of LRAT in PRs.

We first demonstrated that LRAT could function in a nonendogenous, retinoid-rich cellular environment, using the *Gcap1* promoter in mice for PR-specific expression. The retina remained stable in young animals (6 to 8 week old), allowing us to make observations without interference from degeneration. The PR-LRAT mice actively sequestered excess 11-*cis*-retinol, eliminating the endogenous, CRALBP-mediated feedback mechanism (Figure 4). These REs could be formed by 11-*cis*-retinal provided directly by the RPE, which could diffuse to the IS and is reduced by RDHs to form 11-*cis*-retinol, susceptible to LRAT-mediated esterification. Another possibility is that the 11-*cis*-retinol could be released by MG-localized CRALBP and diffuse to the IS, where it is esterified. 11-*cis*-REs were not found in the retina of PR-LRAT mice when CRALBP was globally deleted from the retina, which could be explained by 2 different mechanisms. First, CRALBP deletion in the RPE dramatically slows classical visual cycle kinetics, which could reduce the level of 11-*cis*-retinal present in the IS compartments available for RDH-mediated reduction and subsequent LRAT-mediated esterification. Second, the loss of MG-localized CRALBP could eliminate 11-*cis*-retinol found in the neural retina, thereby eliminating this potential source for PR-LRAT. To probe the first mechanism, we investigated the impact of deleting of the major IS-localized 11-*cis*-retinal reductase, RDH12, on 11-*cis*-RE levels in PR-LRAT mice (25, 32, 40). We found that PR-*Lrat^+/+^Rdh12^–/–^* mice had significantly lower levels of 11-*cis*-REs while all-*trans*-RE production was elevated. This suggests that deletion of *Rdh12* impairs RPE-derived 11-*cis*-retinal reduction and PR-LRAT-mediated esterification of the resulting 11-*cis*-retinol. In the absence of RDH12, the impairment in 11-*cis*-retinal reduction could allow more time for nonspecific *cis*- to *trans* isomerization, which would account for the higher levels of all-*trans*-REs observed in PR-*Lrat^+/+^Rdh12^–/–^* mice. The prolonged presence of all-*trans*-retinal allows this isomer to be slowly reduced and trapped by PR-LRAT and links our finding to a previous hypothesis that RDH12-linked pathogenesis in humans is related to toxic levels of all-*trans*-retinal formed as a result of rhodopsin bleaching (1, 2). As such, our monitoring system provides further insight into the potential mechanisms underlying the severe, retinal-degenerative phenotype observed in humans with *RDH12* mutations (25, 32, 41).

When endogenous LRAT was deleted globally and paired with PR-LRAT low but measurable levels of all-*trans*-RE were observed in the PRs. This suggests that while LRAT in the RPE is critical for isomerization (i.e., visual chromophore synthesis), low levels of all-*trans*-retinol still enter the neural retina from circulation and bypass intermediate sequestration in the RPE. This observation is consistent with the rescue of vision through systemic delivery of synthetic chromophore in *Lrat*-deficient mice (42). Notably, these experiments provided no evidence for an intraretinal visual cycle in mice, as no 11-*cis*-RE was trapped in the PRs when *Lrat* expression in the RPE was abolished.

The PR-REs do not affect the normal rate of chromophore regeneration after bleaching, as observed by ERG and visual cycle kinetics. Successive rounds of intense light exposure increased PR-localized all-*trans*-REs and shifted the ratio of 11-*cis* to all-*trans* retinoids within the neural retinas. This supports our hypothesis that successive exposure to intense light would lead to increased local levels of all-*trans*-retinol substrates for PR-LRAT. We found that the 2 predominant 11-*cis*-REs in the PR-*Lrat^+^* mice were 11-*cis*-retinyl palmitate and 11-*cis*-retinyl stearate. This mirrors the predominant sn-1 fatty acids on phosphatidylcholine (PC) in rat rod outer segments (26).

### Expression of LRAT in MG.

When *Lrat* was delivered to MG via viral transduction, TP imaging showed RE structures spanning the length of MG cell bodies from the GCL to the junction of the PR ONL and IS. Remarkably, we found that the vast majority of REs in the MG-LRAT mice were all-*trans*-REs and their presence was largely CRALBP dependent. This dependence on CRALBP for all-*trans*-RE synthesis was surprising, given the preference that CRALBP exhibits for 11-*cis*-retinol/11-*cis*-retinal versus all-*trans*-retinol/all-*trans*-retinal (15). Ectopic expression of LRAT in MG leads us to conclude that unbound all-*trans*-retinol is far more abundant in murine MG endoplasmic reticulum (ER) membranes than its 11-*cis* counterpart, where it can be esterified by MG-LRAT. CRALBP in the MG could sequester 11-*cis* retinoid isomers and shield them from MG-LRAT esterification. As with PR-*Lrat^+^Lrat^–/–^* mice, all-*trans*-retinol freely diffuses through the MG layer and is trapped as all-*trans*-RE in MG-transduced *Lrat^–/–^* mice. This conclusion is supported by our observation that successive bleaching had no significant effects on all-*trans*-RE accumulation within WT mice transduced with MG-LRAT, indicating that influx of all-*trans*-retinol into the MG remains relatively fixed, even with elevations in all-*trans*-retinol/retinal within the PRs. Our studies of MG-*Lrat^+^Lrat^–/–^* mice showed that trace amounts of all-*trans*-retinol continue to diffuse or be shuttled to the MG independent of an intact visual cycle and that CRALBP is necessary for MG-localized RE synthesis. We speculate that all-*trans*-retinol could be oxidized and utilized by RGR in the photochemical production of 11-*cis*-retinal in MG.

### Expression of LRAT in RGCs.

RGCs occupy the innermost layer of the neural retina, and much is unknown about how a subset of intrinsically photosensitive RGCs obtain visual chromophore (11-*cis*-retinal) for nonvisual light sensing (18). When *Lrat* was introduced by AAV to RGCs, punctate RE structures were observed in the GCL of WT mice using TP imaging. Retinoid analyses showed that the REs within the RGCs were almost exclusively all-*trans*-REs under light-adapted conditions; the HPLC elution characteristics of these REs differed from those of the MG-LRAT and PR-LRAT REs, suggesting a different array of acyl donors in RGC membranes. Notably, the dominant all-*trans*-RE isoforms present under light-adapted conditions shifted to a more balanced ratio of 11-*cis*-REs to all-*trans*-REs when the transduced WT mice were maintained in a darkroom for 6 weeks after transduction. These results indicate the presence of free 11-*cis*-retinol within transduced RGCs during sustained periods of visual cycle inactivity.

### LRAT in human MG and retinoids in human retina.

In our RNA-seq analyses of MG, we observed a remarkable difference between the mouse and human samples. The sc- and sn-RNA-seq datasets detected *LRAT* transcript in human MG but no *Lrat* transcript in mouse MG. Notably, we found a regional difference in the number of human MG expressing LRAT, with a substantial enrichment in *LRAT^+^* MG within the peripheral retina. This distribution refuted our initial hypothesis that more *LRAT^+^* MG would reside in the cone-rich foveal/macular regions if MG were a key source for regeneration of visual chromophore to supply the cones. Thus, we posit that human peripheral MG could increase reserves of 11-*cis*-retinol for visual chromophore regeneration in peripheral PRs (mainly cones) and/or increase rates of retinal reduction (i.e., detoxification) via esterification of retinols using LRAT. Additionally, our bulk-RNA-seq and sn-ATAC-seq analyses of human retina and RPE/choroid indicated that there is a shared, predominant *LRAT* splice form expressed in each tissue and that both RPE and MG have a similar region of open chromatin accessibility at the *LRAT* promoter. The presence of biochemically active LRAT in human MG is supported by RE peaks observed in dissected whole human neural retina and neural retina punches, coupled with our assessment of minimal RPE/choroid cross-contamination of the dissected retinas.

In human retinal organoids, there is a spike in *LRAT* expression within glial cells at later (280 days after seeding) stages of maturation, consistent with the onset of MG differentiation and maturation (43). This finding suggests that the spike in *LRAT* expression within the more mature D260 (260 days after seeding) human retinal organoids could be attributable to late-stage differentiation of “peripheral,” *LRAT^+^* MG, as seen in the mature human sc-RNA-seq datasets. Cumulatively, these results suggest additional, unexplored differences in mouse and human retinoid homeostasis, potentially because of contrasting rod-dominant versus cone-dominant retinae or nocturnal versus diurnal vision, with implications for visual chromophore regeneration and retinoid detoxification in human peripheral retina.

### Limitations.

Observations of the human retina were made with light-adapted, postmortem tissue obtained hours after death. Studies of the mouse retina cannot address certain aspects of retinoid flux occurring in the human retina, particularly within the foveal/ macular regions, as mice lack these structures.

Our murine models have some variance in their genetic backgrounds. Although we use litter-matched controls for many of our comparisons among the PR-*Lrat^+^*, PR-*Lrat^+^Lrat^–/–^*, PR-*Lrat^+^Rlbp1^–/–^*, and PR-*Lrat^+^Rdh12^–/–^* mice, we cannot rule out the effects of varied genetic backgrounds on retinoid content when comparing with *Lrat^–/–^*, *Rlbp1^–/–^*, and *Rdh12^–/–^* lines. Standardizing mouse backgrounds via traditional backcrossing can take more than ten generations, which would have been infeasible for all the lines generated in our study. This shortcoming is mitigated by the use of litter-matched controls and by the length of our dark-adaptation period, but it complicates comparisons made between PR-*Lrat^+^* mice independently crossed with other knockout strains. For our viral transduction experiments, we could not determine the effects of nonspecific transduction and expression of GFAP-LRAT in astrocytes (44). Despite this potential complication, TP imaging and IF staining both indicate regions of MG-specific GFAP-*Lrat* transduction following IVt injection. The same limitation applies to the use of our AAV2-hSyn-*Lrat* construct to transduce RGCs. Initial transduction experiments with AAV2-hSyn-eGFP suggested some off-target transduction of other retinal neural cell types. We did, however, colocalize the majority of retinosome-like signal to RGCs via injection of a fluorophore-labeled, RGC-specific anti-Thy1.2 antibody prior to imaging.

We cannot rule out that the ectopic LRAT expression disrupts normal retinal physiology, though we attempted to address this by comparing our LRAT-knockin mice with other, litter-matched mice with additional genetic perturbations to retinoid-handling genes (e.g., *Rlbp1^–/–^*, *Rdh12^–/–^*, *Lrat^–/–^*). As LRAT likely acts as a powerful, artificial retinoid sink that perturbs endogenous retinoid concentrations within different cellular compartments, we avoided making statements about the RE concentrations found in our LRAT-knockin mice correlating with the absolute abundance of retinol substrates within the cell types we profiled and instead focused on the relative concentrations and ratios of 11-*cis*-RE and all-*trans*-RE isoforms, which can yield information on what retinol species are available within specific cell types for sequestration by LRAT. Overall, we believe that these limitations do not substantially affect our conclusions.

## Methods

### Sex as a biological variable.

For mouse experiments, both male and female animals were used in approximately equal ratios. As the amounts of visual chromophore and visual cycle biochemistry is known to be consistent between male and female mice, sex was not specifically evaluated as a biological variable. For human experiments, the sex of the donors was not disclosed, and given the low biological sample number, sex was not evaluated as a biological variable.

### Animal husbandry.

Animals were housed under 12-hour/12-hour light/dark cycles and fed a standard soy protein-free diet (Teklad 2020X, Envigo) ad libitum. All in vivo and in vitro experiments were performed on both male and female mice in approximately equal numbers.

### Statistics.

Statistical analyses were done using GraphPad Prism version 10 for Mac, unless otherwise stated. Means are reported with SD for unpaired 2-tailed *t* tests, ordinary 1-way ANOVA, and 2-way ANOVA, unless otherwise stated. ANOVAs were followed post hoc by either Tukey’s multiple comparisons tests or Fisher’s LSD tests. *P* values ≤ 0.05 were considered significant.

### Study approval.

All animal procedures complied with the NIH *Guide for the Care and Use of Laboratory Animals* (National Academies Press, 2011) and the ARVO Statement for the Use of Animals in Ophthalmic and Vision Research and were approved by the Institutional Animal Care and Use Committee of University of California, Irvine (protocols AUP-21-096, AUP-21-031, and AUP-24-073). For experiments involving human tissue samples, this study was approved by the Institutional Review Board of the University of Iowa as applicable and adhered to the tenets set forth in the Declaration of Helsinki, with patients providing written, informed consent. For donor eyes, full consent was provided by the next of kin of the donors.

### Data availability.

sc-RNA-seq data have been deposited at NCBI Gene Expression Omnibus (GEO) and are publicly accessible under accession GSE243413 (mouse) and GSE265774 (human). Published RPE/choroid data were obtained from 7 published sc-RNA-seq studies (45–51) and from the GEO database (GSE203499). sn-RNA-seq data on the human retina were obtained from published studies (36, 52–54). Raw data and statistical analyses can be found in the Supporting Data Values file. Additional, processed sc-RNA- and sn-RNA-seq analyses can be found in Supplemental Data Files 1–3.

## Author contributions

Conceptualization: ZJE, PDK, and KP. Methodology: ZJE, GP, SWD, JZ, ZD, CRM, JW, JS, RFM, RC, PDK, and KP. Software: JW, JS, RFM, RC, BAT, and RFM. Validation: ZJE, GP, SWD, JZ, ZD, CRM, JW, JS, RFM, RC, PDK, and KP. Formal analysis: ZJE, GP, SWD, JZ, ZD, CRM, JW, JS, RC, BAT, and RFM. Investigation: ZJE, GP, SWD, JZ, ZD, CRM, JW, JS, RFM, and RC. Resources: ZJE, BAT, RFM, PDK, and KP. Data curation: ZJE, GP, SWD, JZ, ZD, CRM, JW, JS, RFM, RC, and KP. Writing of the original draft: ZJE, GP, SWD, JZ, ZD, CRM, PDK, and KP. Review and editing of the manuscript: all authors. Visualization: ZJE, GP, SWD, JZ, ZD, CRM, JW, JS, RFM, RC, PDK, and KP. Supervision: PDK and KP. Project administration: ZJE, PDK, BAT, RFM, and KP. Funding acquisition: ZJE, SWD, PDK, and KP.

## Funding support

This work is the result of NIH funding, in whole or in part, and is subject to the NIH Public Access Policy. Through acceptance of this federal funding, the NIH has been given the right to make the work publicly available in PubMed Central.

- NIH grants (R01EY009339 to KP, R01EY034519 to KP and PDK, R01EY033308 to BAT and RFM, T32GM008620 and F30EY033642 to SWD, and 5F31EY034027 to ZJE).
- Department of Veterans Affairs grants (I01BX004939 and IK6BX006800 to PDK).
- An unrestricted grant from Research to Prevent Blindness to the Gavin Herbert Eye Institute at the University of California, Irvine.
- NIH core grant P30EY034070 to the Gavin Herbert Eye Institute at the University of California, Irvine.

## Supplementary Material

Supplemental data

Supplemental data set 1

Supplemental data set 2

Supplemental data set 3

Unedited blot and gel images

Supplemental video 1

Supplemental video 2

Supplemental video 3

Supplemental video 4

Supporting data values

## Version 1 11/18/2025

In-Press Preview

## Version 2 02/02/2026

Electronic publication
