# Supplementary material for: Retinol tracing within murine neural retina reveals cell type–specific retinol transport and distribution
Source: J Clin Invest. 2025 Nov 18;136(3):e198648. doi: 10.1172/JCI198648 (PMC12867154; doi:10.1172/JCI198648)
Supplement: Supplemental data [file jci-136-198648-s162.pdf]

SUPPLEMENTARY MATERIALS

Retinol tracing within murine neural retina reveals cell type-specific retinol transport and distribution

Zachary J. Engfer,[1,2] Grazyna Palczewska,[1] Samuel W. Du,[1,2] Jianye Zhang,[1] Zhiqian Dong,[1] Carolline Rodrigues Menezes,[1,2] Jun Wang,[3] Jianming Shao,[3] Budd A. Tucker,[4] Robert F. Mullins,[4] Rui Chen,[1] Philip D. Kiser,[1,2,5,6] Krzysztof Palczewski[1,2,7,8,*]

[1] Brunson Center for Translational Vision Research, Department of Ophthalmology and Visual Sciences, Gavin Herbert Eye Institute, University of California, Irvine, CA 92697, USA

[2] Department of Physiology & Biophysics, University of California, Irvine, CA 92697, USA

[3] Department of Molecular and Human Genetics, Baylor College of Medicine, Houston, TX 77030, USA

[4] Department of Ophthalmology and Visual Sciences, University of Iowa, Iowa City, IA 52242, USA

[5] Department of Clinical Pharmacy Practice, University of California, Irvine, CA 92697, USA

[6] Research Service, VA Long Beach Healthcare System, Long Beach, CA 90822, USA

[7] Department of Chemistry, University of California, Irvine, CA 92697, USA

[8] Department of Molecular Biology and Biochemistry, University of California, Irvine, CA 92697, USA

*Corresponding author: kpalczew@uci.edu (K.P.). Phone: 206-406-3972. Mailing address: 847 Health Sciences Quad, Irvine, CA, 92697-4375.

TABLE OF CONTENTS

SUPPLEMENTAL Materials and Methods
SUPPLEMENTAL Tables
SUPPLEMENTAL Figures and Legends
SUPPLEMENTARY Video Captions

REFERENCES

SUPPLEMENTAL MATERIALS AND METHODS

Mice

Genotyping: Tail tips were collected using sterilized scissors; and digested at 55 °C overnight with agitation in 100 µL Direct Lysis Reagent (Cat #: 401-E, Viagen Biotech, Los Angeles, CA), supplemented with 5% (v/v) of 20 mg/mL proteinase K (Cat #: 501-PK, Viagen Biotech, Los Angeles, CA). The digest reactions were then inactivated by incubation at 85 °C for 1.25 h. The resulting tail digests were used as templates for all genotyping PCR reactions performed in-house, using Gotaq Green Master mix (Cat #: M7123, Promega, Madison, WI), and using the primers listed in **Supplemental Table 1**. *Rlbp1*-knockout genotyping assays were performed using an automated genotyping service provided by Transnetyx (Transnetyx, Cordova, TN). PCR-setup information is provided in **Supplemental Table 4**. Albino mice, utilized for two-photon experiments, were obtained by crossing pigmented mice of different breeds onto the albino C57B6(Cg)*Tyr*$^{c\text{-}2J}$/J background and selecting *Gcap1*^{Lrat} animals with *Tyr*$^{c\text{-}2J}$ albinism.

Gcap1^{Lrat} (PR-*Lrat*$^{+}$) and *Gcap1*^{Rpe65} (PR-r*pe65*$^{+}$) mice: *Gcap1*^{Lrat} and *Gcap1*^{Rpe65} knock-in mouse lines were independently generated by Ingenious Targeting Laboratory (Ingenious Targeting Laboratory, Ronkonkoma, NY), using iTL BF1 (C57BL/6J FLP) cells containing a successfully recombined neomycin cassette and the respective *Lrat* or *Rpe65* transgenes recombined into the native *Gcap1* locus. These cells were independently microinjected into BALB/c blastocysts, and highly chimeric mice were mated to C57BL/6J mice to generate germline neo-deleted mice. Founder animals were screened for Rd1 and Rd8 mutations and the FLP transgene, and crossed for at least two generations with pigmented C57BL/6J animals. *Gcap1*^{Lrat} and *Gcap1*^{Rpe65} progeny were screened to exclude any mutant alleles and the FLP transgene from the mice that were used for experiments.

Lrat$^{-/-}$ mice: *Lrat*$^{-/-}$ mice were initially generated on a 129/Sv background in-lab, then crossed with C57BL/6J to eliminate the Rd8 mutation (1), before extensive backcrossing onto pigmented C57BL/6J and albino C57B6(Cg)*Tyr*$^{c\text{-}2J}$/J backgrounds. Pigmented *Gcap1*^{Lrat}*Lrat*$^{-/-}$ animals were generated by crossing pigmented *Gcap1*^{Lrat} animals with pigmented *Lrat*$^{-/-}$ animals, both bred on the C57BL/6J background.

Cralbp$^{-/-}$*Gnat1*$^{-/-}$ mice: The *Cralbp*$^{-/-}$*Gnat1*$^{-/-}$ double knock-out mouse line was a gift from the Kefalov laboratory (UC Irvine). These mice were generated by crossing *Gnat1*$^{-/-}$ mice (2)and *Cralbp*$^{-/-}$ mice (3). The double knock-out mice were generated on a complex, mixed BALB/c x 129/Sv x C57BL/6J background. Initial *Cralbp*$^{-/-}$*Gnat1*$^{-/-}$ founder mice were screened by the Kefalov lab for *Rpe65* Leu→Met mutant alleles, and bred to have homozygous *Rpe65*$^{Leu/Leu}$ alleles before crossing with *Gcap1*^{Lrat} mice on the C57BL/6J background. These mice were then crossed with *Gcap1*^{Lrat} mice on the C57BL/6J background to generate a variety of *Gcap1*^{Lrat}*Cralbp*$^{-}$*Gnat1*$^{-}$ genotypes.

Rdh12$^{-/-}$ mice: *Rdh12*$^{-/-}$ knock-out mice were obtained from the Jean Bennett laboratory (University of Pennsylvania). These mice were originally generated on a C57BL/6 and BALB/c mixed background (4), and later

outcrossed onto a BALB/c albino background before breeding with pigmented *Gcap1*^{Lrat} mice on a C57BL/6J background to generate independent *Gcap1*^{Lrat}*Rdh12*$^{-/-}$ albino and *Gcap1*^{Lrat}*Rdh12*$^{-/-}$ pigmented mice.

Retinoid analysis

Animals were dark-adapted (DA) overnight prior to euthanasia and collection of eyes for retinoid analysis, unless otherwise stated. To initiate retinoid analysis, eyes were thawed and suspended in 1 mL of retinoid-extraction buffer (pH = 8.0), containing 10 mM sodium phosphate, 100 mM hydroxylamine hydrochloride, and 50% methanol by volume, then homogenized using a glass Dounce homogenizer (cat #: 885501-0021, DWK Life Sciences, Wertheim, Germany). For analyses of whole human neural retina and RPE/ choroid samples, and human neural retina/ RPE punches, the tissue samples were pre-homogenized in 800 µL of 100 mM sodium phosphate buffer (pH = 8.0), using a Dounce homogenizer, and 80 µL of each homogenate was taken for immunoblotting analysis. The remaining 720 µL volume was mixed with 1 mL of retinoid extraction buffer and processed through the remainder of the retinoid analysis protocol. After homogenization, the eye homogenates were transferred to 15-mL conical tubes and incubated for 25 min at room temperature to allow the hydroxylamine to convert retinaldehydes to retinal oximes. Following incubation, 2 mL of 3 M NaCl was added to the homogenates and briefly agitated, followed by 3 mL of ethyl acetate. Tubes containing the homogenates were shaken vigorously for 2 min before centrifugation at 3,220*g* in a tabletop centrifuge for 15 min at 20°C. The top ethyl acetate layers containing retinoids were collected and combined in borosilicate glass tubes. An additional 3 mL of ethyl acetate was added to each sample, and the extraction steps were repeated. The ethyl acetate fractions were combined, and the retinoids were dried under vacuum. After drying, the retinoids were re-dissolved in 400 µL hexanes (or 250 µL in the case of dissected retina and RPE/choroid preparations from transduced RGC-*Lrat*$^{+}$ mice, and human consolidated neural retina and RPE/choroid peri-macular punch preparations) in 1.7 mL Eppendorf tubes, and the mixtures were centrifuged at 16,100*g* in a tabletop centrifuge for 15 min at 4°C to precipitate undissolved material. After centrifugation, the top 350 µL (or 200 µL in the case of dissected retina and RPE/choroid preparations from transduced RGC-*Lrat*$^{+}$ mice and human consolidated neural retina and RPE/choroid punch preparations) of the retinoid extracts were collected, placed into amber HPLC vials with 400 µL glass insets, and loaded onto the HPLC instrument. All steps of retinoid extraction prior to loading onto the HPLC were performed in a darkroom under dim red light. A 100-µL aliquot of each retinoid extract was injected onto an Infinity II series LC instrument (Agilent, Santa Clara, CA) with an attached Agilent Rx-SIL HPLC column (cat #: 880975-901, Neta Scientific, Hainesport, NJ). The elution protocol used eluent A (99.4:0.6% hexanes: ethyl acetate) for 20 min followed by eluent B (90:10% hexanes: ethyl acetate) for 25 min before a switch back to eluent A for 5 min. HPLC runs were conducted at a flow rate of 1.4 mL/min. Eluents were detected spectrophotometrically at 325 nm and 360 nm. Areas under the peaks (in mA) were obtained using the peak integration function on the Agilent OpenLAB CDS ChemStation software (Agilent, Santa Clara, CA). Any peaks below the threshold for automatic peak integration were counted as undetectable (0). Areas under the peaks were multiplied by conversion factors derived from standard curves for 11-*cis*-retinyl esters, all-*trans*-

retinyl esters, *syn*-11-*cis*-retinal oxime, or all-*trans*-retinal oxime, all dissolved in hexanes, to quantify each retinoid in units of pmol/eye.

Two-photon imaging

Two-photon (TP) excitation imaging was accomplished using a custom TP-imaging system based on a Leica TCSSP8 architecture and equipped with a tunable Vision S (Coherent) Ti:sapphire laser, spectral detector, and photon-counting unit for fluorescence decay measurements. The horizontal scanning beam together with the vertical scanning beam spanned the imaged sample at typically 1.2 s per frame, resulting in images typically comprised of 256 by 256 or 512 by 512 pixels (5). In vivo and ex vivo imaging, except for spectral characterization and colocalization experiments, was done with the photon-counting detector in a non-descanned configuration. For the PR-retinosome and cone PR co-localization imaging, two-photon excited fluorescence light was separated into two spectral channels and collected with a descanned detector. Light of 740 nm was used for imaging RPE, PR, Müller glia and GCL-retinosomes. Light of 800 nm was used for imaging of rhodamine-conjugated anti-Thy1.2 (αThy1) antibody injected eyes. For *ex vivo* imaging of enucleated, intact mouse eyes, a 1.0 NA 20x Leica objective was used. For in vivo imaging, an anesthetized mouse was placed on a heated mechanical stage, with its eye covered with GenTeal Lubricant Eye Gel (0.3% Hypromellose, Alcon, Geneva, Switzerland) and a thin 3.2-mm-diameter 0-diopter contact lens (Cantor and Nissel, Northamptonshire, UK) to avoid drying the cornea. To efficiently couple excitation light, and capture fluorescence from the living mouse retina and the RPE, a newly redesigned periscope objective was utilized (6, 7). Spectral measurements and phasor analyses of FLIM data were used to confirm the identities of fluorescing compounds. To form a phasor plot, every pixel of the image was transformed into one phasor point on the phasor plot, such that the coordinates of each phasor point were derived from fluorescence-lifetime decay at the corresponding image pixel, as described previously (6). Phasor times (τp) were determined by the intersection of a straight line from the origin of the semicircle phasor plot through the phasor point of maximum density with the semicircle. Phasor plots were scaled in the same manner as those featured in previous publication (8). Fluorescence emission spectra were collected with a Leica TCSSP8 spectral detector. ImageJ Analyze-Particles software (NIH) was used to quantify the area occupied by retinosomes. LAS X FLIM/FCS 3.5.6 was used for FLIM analyses.

Intravitreal and subretinal injections

Mice were anesthetized by intraperitoneal injection of a cocktail consisting of 20 mg/mL ketamine and 1.60 mg/mL xylazine in PBS, at doses of 100 mg/kg of ketamine and 8 mg/kg of xylazine, and their pupils were dilated by topical administration of 1% tropicamide ophthalmic solution (Akorn, 17478-102-12). The corneas were hydrated with GenTeal Severe Lubricant Eye Gel (0.3% Hypromellose, Alcon, Geneva, Switzerland). Intravitreal injections were performed using an ophthalmic surgical microscope (Zeiss). An incision was made through the sclera 1 mm posterior to the limbus on the nasal side, using a 27-gauge needle. A 34-gauge blunt-end needle (World Precision Instruments; NF34BL-2), connected to an RPE-KIT (World Precision Instruments; RPE-KIT) with SilFlex tubing (World Precision Instruments; SILFLEX-2), was inserted through the scleral incision while avoiding the lens and advanced into the intravitreal space. The needle was further advanced to the subretinal

space in the case of subretinal injections. Each mouse received 1.7×10^{10} genome copies per eye, controlled with a UMP3 UltraMicroPump (World Precision Instruments, UMP3-4). After surgery, the mice were placed on a heating pad and anesthesia was reversed with an intraperitoneal injection of atipamezole in PBS (2.5 mg/kg, cat #: 032800, MWI Animal Health, Boise, ID). Triple-antibiotic ophthalmic ointment (neomycin, polymyxin, and bacitracin) was administered to the cornea to facilitate recovery. For in vivo labeling of cones for two-photon imaging applications, 1 µL of undiluted PNA-rhodamine (cat #: RL-1072, Vector Laboratories) was injected subretinally per eye 48 h prior to sacrifice. For Thy1.2 in vivo labeling of RGCs, mice were injected with 1, 2, or 3 µL anti-Thy1.2 antibody (1.8 mg/mL, rat-anti-mouse CD90 (Thy1.2) monoclonal, clone: FF-10, cat #: MCA1474T, BioRad Laboratories, Hercules, CA) conjugated in-house with rhodamine (degree of labeling = ~1.3).

Histology

Animals were euthanized using CO_2 followed by cervical dislocation. Eyes were removed using forceps and the superior pole of the eye was marked with a permanent marker followed by immersion of the whole eyes in Hartman's fixative (Sigma Aldrich, St. Louis, MO, USA) for 24 h at RT. The eyes were then washed 3 x 5 min with 1x phosphate-buffered saline (PBS, pH 7.4) at room temperature. Following removal of the Hartman's fixative and washing, the whole eyes were dehydrated using a sucrose gradient (10-30% weight/volume) in PBS, and incubated overnight at 4°C in 750 µL of a solution consisting of 1 part 30% sucrose (weight/volume) in PBS and 2 parts O.C.T. medium (Sakura Finetek USA Inc., Torrance, CA, USA). The eyecups were then warmed to room temperature, embedded in O.C.T. medium (Sakura Finetek USA Inc., Torrance, CA, USA) within plastic cryomolds, and frozen on a cooling block immersed in dry ice. Following embedding and freezing, the blocks were stored at -80°C prior to sectioning on a cryostat microtome at 10 µm thickness. Single sections running superior to inferior through the optic nerve were mounted on slides and stained using hematoxylin and eosin (H&E). Stained sections were mounted with coverslips and imaged using a microscope on the bright field setting at 40x magnification prior to stitching full-section images for photoreceptor nuclei counts (Keyence BZ-X800, Keyence, Itasca, IL). For the photoreceptor nuclei counts and construction of spider plots, 3-5 intact rows of photoreceptor nuclei were counted and averaged from stained sections at 200 µm intervals, from the optic nerve to a total distance of 2000 µm along the superior and inferior axes of the retinas. The averaged nuclei counts were grouped by genotype for each point along the retinas for statistical comparisons.

Immunofluorescence (IF) staining of cryosections and wholemounts

For immunofluorescence staining, animals were euthanized with CO_2 followed by cervical dislocation. Eyes were removed using forceps and the superior pole of the eye was marked with a permanent marker; eyes were washed briefly in 1 mL of 1x PBS (pH = 7.4), then immersed in 1 mL of 4% paraformaldehyde dissolved in 1x PBS (pH = 7.4) to fix for 20 min at room temperature and washed 3x 5 min with 1 mL of 1 x PBS (pH = 7.4). Following the wash steps, the lens and cornea were removed from each eye, and the posterior eyecups were washed briefly with an additional 1 mL of 1 x PBS. The PBS was removed, and the eyecups were immersed in 1 mL of fresh 4% paraformaldehyde dissolved in 1x PBS for 30 min at room temperature. The eyecups were then washed 3 x

5 min with 1 mL of 1 x PBS, dehydrated, embedded, and sectioned using the same method described in the Histology section above. Following warming of slides to room temperature, slides were baked at 60°C for 30 min to remove excess moisture. The slides were then cooled to room temperature, placed in a slide rack, and gently immersed in a 50% ethanol: 50% MilliQ water solution for 5 min with occasional agitation. This step was repeated with a 75% ethanol: 25% MilliQ water solution for 5 min, followed by two successive immersions in 100% ethanol for 5 min. The slides were dried flat, then a hydrophobic barrier was drawn around each section with a Vector Labs ImmEdge™ pen (cat #: H4000, Vector Laboratories, Newark, CA). The slides were blocked for 30 min at room temperature in a 0.45 µm filter-sterilized IF-blocking buffer containing 1% bovine serum albumin (mass/volume; Cat #: A7030, Millipore Sigma, Burlington, MA), 5% volume/volume normal goat serum (Cat #: G9023, Millipore Sigma, Burlington, MA), and 0.2% Triton-X-100 (Cat #: A16046, Thermo Fisher Scientific, Waltham MA) dissolved in 1x Dulbecco's PBS supplemented with calcium and magnesium (Cat #: 14040133, Thermo Fisher Scientific, Waltham MA). Following the blocking step, the IF-blocking solution was removed and replaced with primary-antibody solution diluted in fresh IF-blocking buffer and incubated for 24 h at 4°C. Secondary-only controls were incubated in fresh IF-blocking buffer only for 24 h at 4°C. Following incubation with the primary-antibody solution, the slides were washed 3x 5 min with fresh IF-blocking buffer at room temperature, and then the blocking buffer was replaced with secondary-antibody solution diluted in fresh IF-blocking buffer and incubated for 1 h at room temperature protected from light. Peanut agglutinin (PNA)-rhodamine was added at the secondary-antibody incubation stage. The slides were then washed 3x 5 min and mounted with VectaShield® HardSet™ antifade mounting medium with DAPI (Cat #: H-1500-10, Vector Laboratories, Newark, CA). Fluorescence images were obtained using a Keyence fluorescence microscope at 20X magnification (Keyence BZ-X800, Keyence, Itasca, IL). Z-stacks were processed using the Keyence BZ-X800 analysis software (Keyence, Itasca, IL). Confocal images were obtained using a Leica Stellaris SP8 confocal microscope system (Leica, Wetzlar, Germany) at 63X magnification; Z-stacks were processed using Fiji (9).

For neural retina wholemounts, the same enucleation and fixation protocol was used as for IF staining (above). Following 30 min of eyecup fixation, the eyecups were washed 3 x 5 min with 1 x PBS (pH = 7.4), and the eyecup was flattened by making 4-5 incisions with scissors starting at equidistant points around the rim of the eyecups 2/3 of the way to the optic nerve head in the center of the eyecups. After making the incisions and flattening the eyecup, the neural retina was mechanically dissociated from the underlying RPE/choroid by gently inserting forceps between the two layers and moving them to free the neural retina. Following removal of the whole neural retinas, they were placed in a 24-well tissue culture plate (cat #: 25-107, Genessee Scientific, El Cajon, CA) containing 500 µL of fresh IF-blocking buffer. The wholemounts were blocked for 30 min at room temperature with gentle rocking. The blocking buffer was then removed and replaced with 500 µL of primary-antibody solution diluted in IF-blocking buffer, and the tissues were incubated in primary solution for 48 h at 4°C with gentle rocking. The neural retina wholemounts were washed 3x 5 min with fresh IF-blocking buffer and then incubated in 500 µL of secondary-antibody solution in IF-blocking buffer for 1 h with gentle rocking and protection from light. After incubation with the secondary-antibody solution, the tissues were washed 3x 5 min with fresh IF-blocking buffer

and then mounted with VectaShield® HardSet™ antifade mounting medium with DAPI (Cat #: H-1500-10, Vector Laboratories, Newark, CA). Fluorescence images were obtained using a Keyence fluorescence microscope at 20X magnification (Keyence BZ-X800, Keyence, Itasca, IL) and then stitched to create full-field wholemount images using the Keyence BZ-X800 analysis software (Keyence, Itasca, IL).

Antibodies used:

Primaries: Mouse anti-mouse monoclonal LRAT, generated and purified in-house (1), 1:100 dilution (immunoblotting and IF); rabbit anti-mouse monoclonal CRALBP primary, Cat #: ab183728, Abcam, Cambridge, UK, 1:250 dilution (IF); rabbit anti-mouse polyclonal M-cone opsin, Cat #: NB110-74730, Novus Biologicals, Centennial CO, 1:1000 dilution (IF); rabbit anti-mouse polyclonal *S*-cone opsin, Cat #: NBP1-20194, Novus Biologicals, Centennial CO, 1:1000 dilution (IF); 1:100 dilution; mouse anti-mouse monoclonal rhodopsin antibody (1D4), purified in-house (10), 1:1000 dilution (immunoblotting); mouse anti-mouse monoclonal RPE65 primary (KPSA1), generated and purified in-house (11), 1:500 dilution (immunoblotting), 1:250 dilution (IF); rabbit anti-mouse monoclonal β-actin primary (13E5), Cat #: 4970, Cell Signaling Technology, Danvers, MA, 1:1000 dilution (immunoblotting); rabbit anti-mouse monoclonal α-tubulin, Cat #: 2144, Cell Signaling Technology, Danvers, MA. 1:1000 dilution (immunoblotting).

Secondaries: Goat anti-mouse IgG AF-647, Cat #: A-21236, Thermo Fisher Scientific, Waltham MA, 1:250 dilution (IF); goat anti-rabbit IgG AF-488, Cat #: A-11034, Thermo Fisher Scientific, Waltham MA, 1:500 (IF). Donkey anti-mouse IgG IR-680RD, Cat #: 926-68072, LI-COR, Lincoln, NE, 1:2000 dilution (immunoblotting); donkey anti-rabbit IgG IR-800CW, Cat #: 926-32213, LI-COR, Lincoln, NE, 1:2000 dilution (immunoblotting).

Auxiliary: PNA-rhodamine, Cat #: RL-1072, Vector Laboratories, Newark, CA, 1:500 dilution (IF).

Immunoblotting

Mice were euthanized using CO_2, followed by cervical dislocation. Eyes were enucleated and placed in chilled 1x PBS (pH = 7.4) on ice prior to dissection. Then, the lens and cornea were removed from each eye and the neural retina was gently peeled away from the underlying RPE/choroid material using forceps. The optic nerve and adipose tissue surrounding each eyecup were removed with scissors. The isolated neural retina and RPE/choroid were removed from PBS, placed in individual 1.7 mL tubes, and then frozen on dry ice. Tissues were stored at -80°C prior to homogenization and immunoblotting. Dissected mouse neural retina and RPE/choroid were independently homogenized in 200 µL of chilled 1 x RIPA lysis and extraction buffer (cat #: 89900, Thermo Fisher Scientific, Waltham, MA) in 1x PBS (pH = 7.4) supplemented with protease inhibitors (cat #: 11836170001, Millipore Sigma, Burlington, MA) for 30 s using a handheld homogenizer equipped with disposable pestles (cat #: 12-141-364, Thermo Fisher Scientific, Waltham, MA). Following an initial homogenization step, each homogenate was placed on ice. Samples were briefly centrifuged at 1000*g* for 30 s in a tabletop centrifuge at RT, then placed back on ice and sonicated for 20 s total in 5 s on, 5 s off intervals (25% amplitude), using a Qsonica Q500 sonicator (cat #: Q500A-110, Qsonica sonicators, Newtown, CT) equipped with a 5/64" probe. The samples were then centrifuged at 21,130*g* in a tabletop centrifuge for 10 min

at RT to precipitate un-solubilized debris. The cleared supernatants for each sample were harvested and transferred to fresh 1.7-mL Eppendorf tubes and 15 µL of each cleared sample was mixed with 4.5 µL of 4 x Laemmli buffer (cat #: 1610747, BioRad Laboratories, Hercules, CA) and 0.5 µL of β-mercaptoethanol (cat #: M6250-10ML, Millipore Sigma, Burlington, MA). 15 µL of each sample was loaded onto 4-20% Mini-PROTEAN TGX precast protein gels (cat #: 4561093, Bio-Rad Laboratories, Hercules, CA) and run in 1x Tris/Tricine/SDS running buffer (cat #: 1610744, Bio-Rad Laboratories, Hercules, CA). A lane with a Precision Plus Dual-Color protein standard ladder (Cat #: 1610374, Bio-Rad Laboratories, Hercules, CA) was run alongside the sample lanes. The gels were then blotted using a semi-dry electrophoretic transfer cell (Cat #: 1703940, Bio-Rad Laboratories, Hercules, CA) onto Immobilon-P™ PVDF membrane (cat #: IPVH00010, Thermo Fisher Scientific, Waltham, MA) activated briefly in methanol and equilibrated 15 min in Tris-glycine-methanol transfer buffer (0.025 M Tris base, 0.189 M glycine, 4.94 M methanol). Gels were equilibrated for 10 min in the same transfer buffer prior to blotting. After blotting, the membranes were incubated in 10 mL 1 x PBS-t (pH = 7.4; 0.05% volume/volume Tween®-20 (cat #: P1379-250ML, Sigma-Aldrich, St. Louis, MO)) at room temperature with gentle rocking. Then, the membranes were blocked in 5 mL of 5% (weight/volume) milk (cat #: M17200-1000.0, Research Products International, Mt Prospect, IL) dissolved in PBS-t for 30 min at room temperature with gentle rocking. The membranes were then placed in a primary-antibody solution diluted with 5 mL of 1% (weight/volume) milk in PBS-t and incubated for 24 h at 4°C with gentle rocking. After incubation with the primary-antibody solution, membranes were washed 3x 5 min with PBS-t and then placed in secondary-antibody solution diluted in 5 mL of PBS-t for 1 h rocking at room temperature, protected from light. Blots were then washed 3 x 5 min with PBS-t and imaged on a BioRad ChemiDoc™ MP Imaging System (BioRad Laboratories, Hercules, CA).

For human samples, 80 µL of dounce-homogenized tissue in 100 mM sodium phosphate buffer (pH = 8.0) prepared for retinoid analysis (see the Retinoid analysis section) was mixed with 80 µL of 2x RIPA buffer. Samples were then cleared by centrifugation at 21,130*g* at room temperature. The cleared samples were transferred to new tubes and 15 µL of each was mixed with 5 µL of Laemmli + β-mercaptoethanol; 10 µL of each mixture was loaded onto 4-20% Mini-PROTEAN TGX Precast Protein gels for electrophoresis and blotting. For diluted RPE65 blots, 4 µL of sample was diluted with 11 µL of 1x RIPA buffer and then mixed with 5 µL of Laemmli + β-mercaptoethanol. The remainder of the blotting protocol was kept consistent with that for the mouse samples.

Bulk mRNA purification, cDNA synthesis, and PCR/ qPCR of cDNA templates

<u>Primary human tissue bulk RNA isolation and first-strand cDNA synthesis</u>: Bulk RNAs were purified from whole human retina and RPE/choroid tissues through manual homogenization of frozen tissue in 200 µL of QIAzol Lysis Reagent (Cat #: 7936, Qiagen, Germantown, MD) for 30 s, using disposable, nuclease-free plastic pestles (cat #: 12-141-364, Thermo Fisher Scientific, Waltham, MA). The volume of QIAzol was then raised to 700 µL, and RNA from each sample was extracted using the Qiagen miRNAeasy Mini kit (cat #: 217004, Qiagen, Germantown, MD). Total RNA (200 ng) extracted from each sample was used for random hexamer-primed, first-strand cDNA-synthesis reactions using SuperScript™ III First-Strand Synthesis SuperMix (Cat #: 18080400,

Thermo Fisher Scientific, Waltham, MA). The resulting cDNA-synthesis solutions were diluted 1:1 with nuclease-free water, then 1 μL aliquots of the resulting mixtures were used for PCR amplifications of the cDNA coding regions of *LRAT*, *RPE65*, and *GAPDH*; 1 μL of each undiluted human retina and RPE/choroid cDNA-synthesis solution was used as a template for real-time-qPCRs of *LRAT*, *RPE65*, and *GAPDH*.

Mouse bulk RNA isolation and first-strand cDNA synthesis: The same extraction protocol as above was used for bulk RNA isolation from dissected mouse neural retina and RPE tissues. 75 ng of each RNA extraction was used for first-strand cDNA synthesis using SuperScript™ III First-Strand Synthesis SuperMix (Cat #: 18080400, Thermo Fisher Scientific, Waltham, MA). 1 μL of each mouse retina and RPE/choroid cDNA reaction was used as a template for PCR amplification of RPE65 cDNA coding regions.

LRAT, *RPE65*, and *GAPDH* cDNA coding-region PCRs from human neural retina and RPE/choroid samples: One μL of each neural retina and RPE/choroid cDNA template was diluted 1:1 with nuclease-free water, then 1 μL of the resulting mixture was used for 50 μL *LRAT*, *RPE65*, and *GAPDH* PCR amplifications of the cDNA coding regions using Phusion™ Plus Green PCR Master mix (Cat #: F632S, Thermo Fisher Scientific, Waltham, MA) and custom primers designed to specifically amplify *LRAT*, *RPE65*, and *GAPDH* coding regions (see **Tables S1** and **S2**). *RPE65* coding-region amplicons from human retina and RPE cDNAs were purified using a Qiagen MinElute PCR Purification Kit for PCR cleanup (Cat #: 28004, Qiagen, Germantown, MD); their sequences were confirmed by Oxford Nanopore (ONT) sequencing services offered through Plasmidsaurus (Plasmidsaurus, San Francisco, CA). LRAT coding-region amplicons from human retina and RPE cDNAs were gel extracted using a Monarch® DNA gel extraction kit (Cat #: T1020, New England Biolabs, Ipswich, MA), then the gel-extracted amplicons were re-amplified using Phusion™ Plus Green PCR Master mix (Cat #: F632S, Thermo Fisher Scientific, Waltham, MA) with HiFi adapter primers for assembly into a linearized pUC-19 vector for cloning. pUC-19 vectors were linearly amplified using Phusion™ Plus Green PCR Master mix (Cat #: F632S, Thermo Fisher Scientific, Waltham, MA). *LRAT* HiFi-adapter amplicons and the linear pUC-19 amplicon were purified using a Qiagen MinElute PCR Purification Kit for PCR cleanup (Cat #: 28004, Qiagen, Germantown, MD); the purified PCR products were then ligated together using NEBuilder® HiFi DNA Assembly Cloning Kit (Cat #: E5520S, New England Biolabs, Ipswich, MA). The ligation reactions were transformed into Stellar chemically competent cells (Cat #: 636766, Takara Bio, San Jose, CA), then colonies were picked, grown, and miniprepped using a PureLink™ Quick Plasmid Miniprep Kit (Cat #: K210010, Thermo Fisher Scientific, Waltham, MA). The miniprepped plasmids containing the *LRAT* coding-region amplicons from human neural retina and RPE/choroid were then sequenced by O.N.T., as described above (Plasmidsaurus, San Francisco, CA).

Signal intensity quantification of coding-region PCR amplicons for *LRAT*, *RPE65*, and *GAPDH*: Five μL of each coding-region PCR reaction was loaded onto a 1.75% agarose gel mixed with SYBR-Safe (Cat #: S33102, Thermo Fisher Scientific, Waltham, MA) and run for 27 min at 200 V. The gel was imaged using SYBR-Safe auto-exposure settings on a BioRad ChemiDoc™ MP Imaging System (BioRad Laboratories, Hercules, CA). The TIF image of the gel was then imported into FIJI, converted to grayscale, and analyzed using the Analyze ->

Gels tool (9). The resulting histograms for each lane were taken and the area-under-the-curve of each signal was measured for each lane. The signal intensity measurements were then plotted.

LRAT, *RPE65*, and *GAPDH* real-time qPCRs from human neural retina and RPE/choroid cDNAs: One µL of each neural-retina and RPE/choroid cDNA-template reaction and 1 µL of each qSTAR primer mix was used for each 10 µL qPCR reaction, using Applied Biosystems PowerUp SYBR Green Master Mix (Cat #: A25742, Thermo Fisher Scientific, Waltham, MA). Reactions were set up in technical duplicate within 384-well thin-wall plates (Cat #: HSP3805, Bio-Rad Laboratories, Hercules, CA), sealed (Cat #: MSB1001, Bio-Rad Laboratories, Hercules, CA), and run on a Bio-Rad CFX384 Real-Time qPCR System (Bio-Rad Laboratories, Hercules, CA); raw Cq values were obtained on the accompanying Bio-Rad CFX Maestro 1.1 software (Ver: 4.1.2433.1219, Bio-Rad Laboratories, Hercules, CA). After averaging Cq values from technical duplicates, ΔCq values for comparing relative amounts of transcript in neural retina and RPE/choroid samples were obtained by performing the following calculation: $2^{-(\mathit{LRAT}\ or\ \mathit{RPE65}\ \text{mean Cq} - \mathit{GAPDH}\ \text{mean Cq})}$ and then plotted.

Coding-region PCR of *Rpe65* from mouse retina and RPE/choroid cDNA templates: One µL of each mouse neural retina and RPE/choroid cDNA template was used for 50 µL PCR reactions using Phusion™ Plus Green PCR Master mix (Cat #: F632S, Thermo Fisher Scientific, Waltham, MA). Primer information is provided in **Table S3** and PCR setup information is listed in **Table S4**.

Analysis of single-cell/single-nuclei in RPE/choroid and retina samples

Published RPE/choroid data were obtained from seven published sc-RNA-seq studies (12-18), and from the Gene Expression Omnibus (GEO) database (GSE203499). Single-nuclei RNA-sequencing (sn-RNA-seq) data of the human retina were obtained from published studies (19-22).

To obtain new data, sc-RNA-seq libraries were prepared using the Chromium Next GEM Single Cell 3' Kit v3.1 (10x Genomics) and sequenced on the Illumina NovaSeq 6000 platform (http://www.illumina.com). We processed the sc-RNA/sn-RNA-seq data according to the methods described in a previous study.(22) Briefly, reads were aligned using Cell Ranger (version 7.1.0), and ambient RNA contamination was corrected using SoupX. Low-quality cells were excluded, *i.e.*, those with fewer than 300 features, fewer than 500 transcript counts, or with over 5% (sn-RNA-seq cutoff) or 10% (sc-RNA-seq cutoff) of reads mapping to mitochondrial genes. Cell quality control was performed using cellqc (https://github.com/lijinbio/cellqc). Batch correction and data integration were performed using scVI (23). Major cell classes were annotated based on known marker genes from previous studies (for RPE/choroid sc-RNA-seq data)(12) or published reference-cell atlas (for retina sn-RNA-seq data)(24).

Single-nuclei Assay for Transposase-Accessible Chromatin sequencing (snATAC-seq) data from human RPE/choroid and retina were generated according to the methods described in a previous publication (22). Briefly, libraries for snATAC-seq were prepared with the Chromium Next GEM Single Cell ATAC Library and Gel Bead Kit v.1.1 (10x Genomics) and sequenced on an Illumina NovaSeq 6000 platform. The snATAC-seq data

from RPE/choroid and retina were processed using ArchR, following the same pipeline as in the previous study (22). Key steps included filtering out low-quality cells, removing doublets, clustering cells, annotating cell types by integrating sn-RNA-seq reference data, calling peaks, and generating BigWig files.

Identification of *LRAT*⁺ Müller glia in the neural-retina CellxGene single-cell/ single-nuclei atlases

Mouse retina sc-RNA-seq data (25) were downloaded from the CellxGene portal (https://cellxgene.cziscience.com/e/23f77ae6-10af-4307-b136-76b26654ae7d.cxg/). Human-retina sn-RNA-seq data (22), were obtained directly from the authors. Gene expression data were log-normalized using the Scanpy package prior to analysis. To compare normalized LRAT expression data for mouse *vs*. human Müller glia, the Mann–Whitney U test was performed on full human and mouse sc-RNA-seq datasets using the mannwhitneyu function in the scipy.stats package. Within the human retina sn-RNA seq dataset, LRAT expression differences across the fovea, peripheral, and macular regions were assessed using the Wilcoxon rank-sum test, as implemented using scanpy.tl.rank_genes_groups. For this comparison of regional differences in MG-localized LRAT expression, the human sc-RNA-seq dataset was down-sampled to a maximum of 5,000 cells per major cell class. For total human sn-RNA seq and mouse sc-RNA-seq UMAP generation to compare LRAT$^+$ MG, all cells for the human (22), and mouse (25) datasets were filtered, selecting cells with a LRAT expression value above zero, using the gene search tool and the rectangular marquee tool on the gene expression histogram to only display cells with an LRAT expression values >0. Images of the filtered UMAPs from the human and mouse atlases were downloaded for inclusion in **Figure 8G**.

For assessing LRAT expression as a function of age group, all annotated human Müller glia from the sn-RNA-seq dataset were isolated using the cell type isolation toolbar, and the same LRAT thresholding approach was applied to obtain the number of *LRAT*$^+$ Müller glia relative to the total number of Müller glia. The percent of total Müller glia that were *LRAT*$^+$ was subdivided as a function of age group; cells belonging to the "80-year-old and over stage" and "adult stage" were omitted due to the lack of specificity in age grouping (**Supplemental File 3**). The percentage of *LRAT*$^+$ Müller glia as a function of age group was compiled in Microsoft Excel (Microsoft, Redmond, WA), then plotted in Graphpad Prism version 10 for Mac (GraphPad Software, Boston, MA, USA, www.graphpad.com), and a simple linear regression was run to assess whether the %*LRAT*$^+$ cells differed as a function of age group (slope + 95% confidence bands).

Bulk RNA-seq and pseudo-bulk sc-RNA-seq data analyses

For one adult human RPE/choroid sample used for visualization with IGV, the library was prepared using the Chromium Next GEM Single Cell 5' Kit v2.0. Raw sequencing reads were mapped to the human reference genome (hg38) using STAR (2.7.10b), following a previously published study (26). The bulk RNA-seq data for the human adult retina samples were obtained from a previous publication (24). and were aligned to the human reference genome (hg38) using STAR (2.7.8a).

Motif identification

We identified the motifs in the promoter region and the strong enhancer (chr4:154674477-154674977) of *LRAT* using HOMER2 (27). We further selected the motifs associated with the transcription factors that display expression patterns similar to *LRAT* and are highly expressed in RPE and Müller glial cells.

Optical coherence tomography (OCT)

OCT was performed on mice anesthetized with ketamine (20 mg/mL) and xylazine (1.75 mg/mL) injected intraperitoneally (IP) at 0.1 to 0.13 mL/25 g body weight (bw), using standard methods detailed (28, 29). All tomograms were taken using a Bioptigen spectral-domain OCT device (Leica Microsystems Inc., Buffalo Grove, IL). Four frames of OCT B-scan images were acquired from a series of 1200 A-scans. Thickness of the outer nuclear layer (ONL) was measured at 500 µm from the optic nerve head at nasal, temporal, superior, and inferior quadrants for each eye; the values were averaged to determine the average ONL thickness for each animal.

Scanning laser ophthalmoscopy (SLO)

Baseline scanning laser ophthalmoscopy was performed on mice anesthetized with ketamine and xylazine (see **OCT** section for details), as detailed in Leinonen et al.,(28) using a Heidelberg Retinal Angiograph II (Heidelberg Engineering, Franklin, MA, USA) SLO machine in autofluorescence mode.

Electroretinography (ERG)

Prior to scotopic ERG recordings, mice were dark adapted for 24 h. Under a safety light, mice were anesthetized by isoflurane inhalation, and their pupils were dilated with 1% tropicamide ophthalmic solution (cat #: 17478-102-12, Akorn Operating Company) and 10% phenylephrine ophthalmic solution (cat #: 054243 MWI Animal Health, Boise, ID). 2.5% hypromellose (cat #: 9050-1, Akorn Operating Company) was then applied for corneal hydration. Each mouse was placed on a heated Diagnosys Celeris rodent-ERG device (Diagnosys LCC, Lowell, MA, USA). Ocular stimulator electrodes were placed on the corneas, and the reference and ground electrodes were positioned subdermally (between ears, and in the rear leg, respectively). For scotopic recordings, the eyes were stimulated with a green-light (peak emission 544 nm, bandwidth ~160 nm) of -0.3 log [cd • s/m^{-2}]. The responses for 10 stimuli, with an inter-stimulus interval of 10 s, were averaged. The a- and b-wave amplitudes were acquired from the averaged ERG waveform and manually inspected. Prior to photopic ERG recordings, mice were kept in a lighted vivarium. Topical dilation, induction of anesthesia, and placement on a rodent-ERG device were performed as described for scotopic recordings. Ocular stimulator electrodes were placed on the corneas; the ground electrode was positioned subdermally in the rear leg. To measure M-cone and S-cone function, stimulation was performed with alternating green- and UV-light pulses at increasing intensities. Green light stimulation had the prescribed characteristics described for scotopic recordings and intensity increments of −0.5, 0.5, 1.5, and 2.5 log [cd • s/m^{-2}]. UV light stimulation (peak emission 370 nm, bandwidth 50 nm) had intensity increments of −1, 0, 1, and 2 log [cd • s/m^{-2}]. The responses for 20–25 stimuli were averaged, and the a- and b-wave responses were acquired from the averaged ERG waveform. Data were analyzed with Espion V6 software (Diagnosys LCC).

Photobleaching and recovery

For assessing visual-cycle kinetics in PR-*Lrat*$^{+}$ mice, all mice were dark-adapted overnight (>12 h) prior to bleaching with light. Mice were treated with a 1% tropicamide ophthalmic solution (Cat #: 119758, Amerisource Bergen, Conshohocken, PA 19428) under dim red light and allowed to rest for15 min to allow for adequate pupil dilation. The mice were then placed in glass cubicles lined with mouse bedding material surrounded by white utility buckets and positioned underneath 10,000 lux LED lamps for 10 min to fully isomerize 11-*cis*-retinal present in the photoreceptors. Once the bleaching regimen was complete, mice were either immediately sacrificed or placed back in the darkroom to allow for progression of the visual cycle followed by sacrifice. For 3-day successive bleaching experiments, light-adapted mice were treated with tropicamide and bleached with 10,000-lux light for 5 min twice a day, spaced at 5 h intervals, using the same setup as described above. Mice were kept on the standard 12 h light / 12 h dark cycle during the sequential bleaching period. Following the 3 days of sequential bleaching, mice were dark-adapted for 24 h prior to sacrifice for retinoid analyses.

Alkaline hydrolysis of retinyl esters

Retinyl esters were collected and combined from four consecutive HPLC runs of whole-eye retinoid extracts from 12-week-old dark-adapted PR-*Lrat*$^{+/+}$ mice (100 µL injections per run). The combined, purified retinyl ester fractions were protected from light and dried under a stream of nitrogen gas. Following drying, the retinyl esters were resuspended in 4 mL methanol, and divided into two 2-mL aliquots. One aliquot was dried and taken through retinoid extraction in ethyl acetate (as described under Retinoid Analysis, above). Following partitioning into ethyl acetate, the retinoids were dried under vacuum and resuspended in 400 µL of hexanes for the starting material control run. To hydrolyze the retinyl esters and release retinol, the other 2-mL aliquot was combined with 3 mL of 0.5 M KOH in methanol and incubated at 42 °C for 15 min, protected from light. The resulting solution was taken through retinoid extraction in ethyl acetate in the same manner as the starting-material control. Following resuspension of each sample in 400 µL of hexanes, 100 µL of each sample was injected onto the HPLC consecutively for analysis, using the same method, materials, and instrument listed in the Retinoid Analysis section.

Mass spectrometry

A pair of PR-*Lrat*$^{+/-}$ mouse eyes were homogenized in 1 mL of methanol/water 1:1 (volume/volume). The resulting mixture was diluted with 2 mL of sodium chloride solution (5 M), extracted with ethyl acetate (2 x 3 mL), and centrifuged at 17,000*g* at 4°C for 15 min. The upper organic layer was dried under vacuum at room temperature. The resulting residue was reconstituted with 200 µL of acetone/acetonitrile (1/4, volume/volume) and centrifuged at 17,000*g* for 15 min at 4°C. Fifty microliters of the supernatant were injected into a Vanquish HPLC system (Thermo Fisher Scientific, Waltham, MA) coupled with a diode array detector (DAD) and a Q Exactive Orbitrap Mass Spectrometer. Elution was carried out on an ACQUITY BEH C18 Column (1.7 µm, 2.1 mm x 50 mm, Waters, Milford, MA) at a flow rate of 0.3 mL/min, using a mixture of 0.1% formic acid in water (A), and 0.1% formic acid in acetone/acetonitrile (1/4, volume/volume) (B) as the mobile phase at the following gradient: 0-1 min, 90% B; 3 min, 100% B; 15 min, 100% B. The acquisition was performed with a positive electrospray ionization mode in full scan (m/z 200–1000), between 3-15 min.

SUPPLEMENTARY TABLES

Table S1. Primers used for PCR amplification of the coding regions of human *LRAT*, *RPE65*, and *GAPDH* cDNA templates, for HiFi ligation of LRAT cDNA amplicons into pUC-19 for sequencing, and real-time-qPCR.[a]

LRAT-5'-cDNA-code-amp-F	CATGCTGGAGGTGGTGTCTTTACTAC
LRAT-3'-cDNA-code-amp-R	TACACACACTGACATGGGGGTATGAAG
LRAT-5'-cDNA-code-amp-HiFi-F	TATCACGAGGCCCTTTCGTCCATGCTGGAGGTGGTGTC
LRAT-3'-cDNA-code-amp-HiFi-R	GTCATCACCGAAACGCGCGATACACACACTGACATGGG
pUC19-HiFi-F	TCGCGCGTTTCGGTGATG
pUC19-HiFi-R	GACGAAAGGGCCTCGTGATAC
RPE65-5'-cDNA-code-amp-F	GTTGAGCATCCTGCTGGTGGTTACAAG
RPE65-3'-cDNA-code-amp-R	CCAAAAACATATCTTGCTGGAGTATGC
GAPDH-5'-cDNA-code-amp-F	GGAAGGTGAAGGTCGGAGTC
GAPDH-3'-cDNA-code-amp-R	CCTCTTGTGCTCTTGCTGGG
qSTAR-hLRAT-F	TGCGAGCACTTCGTGACCTACT
qSTAR-hLRAT-R	GCCAATCCCAAGACTGCTGAAG
qSTAR-hRPE65-F	TTTGGCACCTGTGCTTTCCCAG
qSTAR-hRPE65-R	GTTGGTCTCTGTGCAAGCGTAG
qSTAR-hGAPDH-F	GTCTCCTCTGACTTCAACAGCG
qSTAR-hGAPDH-R	ACCACCCTGTTGCTGTAGCCAA

[a] These primers were used for PCR reactions from neural-retina and RPE/choroid cDNA templates, subcloning of cDNA amplicons into pUC-19 for sequencing, and commercially purchased human qSTAR primer sets (OriGene Technologies, Rockville, MD).

Table S2. Reaction setup, thermocycler conditions, and expected amplicon sizes for PCR and qPCR reactions.[a]

Gene/ PCR Target	Primer Pairing	Thermocycler Conditions	Expected Amplicon Size(s)	Notes
PR-*Lrat*/ PR-*Rpe65* genotyping	WT and Mutant co-PCR:<br>PR-LRAT/RPE65-Geno1-F +<br>PR-LRAT/RPE65-Geno2-F +<br>PR-LRAT/RPE65-Geno3-R	PR-*Lrat*/ PR-*Rpe65* Knock-In PCRs:<br>1. 95 °C for 2 min<br>2. Denature 95 °C for 30 s<br>3. Anneal 57 °C for 30 s<br>4. Extension 72 °C for 45 s<br>5. Final Extension 72 °C for 5 min<br>Store at 4 °C<br>Repeat Cycles 2-4 30X	WT allele: 475 bp<br>KI allele: 590 bp	25 µL reaction volume. 2.5 µL of 10 µM primers used.<br>Tail lysates were diluted 1:50 in nuclease-free water for PCR reaction.
RD8 Mutation Check	WT allele PCR:<br>Rd8-F1 + Rd8-R<br>Mutant allele PCR:<br>Rd8-F2 + Rd8-R	1. 95 °C for 5 min<br>2. Denature 95 °C for 30 s<br>3. Anneal 65 °C for 30 s<br>4. Extension 72 °C for 30 s<br>5. Final Extension 72 °C for 7 min<br>Store at 4 °C<br>Repeat Cycles 2-4 35X	WT allele:<br>220 bp<br>Mutant allele:<br>244 bp	25 µL reaction volume. 2.5 µL of 10 µM primers used.

RD1 Mutation Check	WT allele PCR:<br>Rd1-Common + Rd1-WT<br>Mutant allele PCR:<br>Mutant allele PCR:<br>Rd1-Common + Rd1-Mutant	1. 95 °C for 2 min<br>2. Denature 95 °C for 30 s<br>3. Anneal 60 °C for 30 s<br>4. Extension 72 °C for 1 min<br>5. Final Extension 72 °C for 5 min<br>Store at 4 °C<br><br>Repeat Cycles 2-4 35X	WT allele: 318 bp<br><br>Mutant allele: 550 bp	25 μL reaction volume. 2.5 μL of 10 μM primers used.
FLP check	FLP Check PCR:<br>FLP-Check-F + FLP-Check-R	1. 95 °C for 2 min<br>2. Denature 95 °C for 30 s<br>3. Anneal 55 °C for 30 s<br>4. Extension 72 °C for 30 s<br>5. Final Extension 72 °C for 5 min<br>Store at 4°C<br><br>Repeat Cycles 2-4 30X	FLP (+): 330 bp<br><br>FLP (-): no band	25 μL reaction volume. 2.5 μL of 10 μM primers used.
Lrat$^{-/-}$ Genotyping Reaction	*Lrat* WT allele PCR:<br>LRAT-KO-Shared + LRAT-KO-WT<br>*Lrat* KO allele PCR:<br>LRAT-KO-Shared + LRAT-KO-Knockout	*Lrat* WT/ KO allele PCRs:<br>1. 95 °C for 3 min<br>2. Denature 95 °C for 30 s<br>3. Anneal 60 °C for 30 s<br>4. Extension 72 °C for 2 min<br>5. Final Extension 72 °C for 5 min	*Lrat* WT allele: 300 bp<br><br>*Lrat* KO allele: 370 bp	25 μL reaction volume. 2.5 μL of 10 μM primers used.

		Store at 4 °C<br><br>Repeat Cycles 2-4 30X		
Rdh12$^{-/-}$ Genotyping Reaction	*Rdh12* WT allele PCR:<br>RDH12-KO-Shared-F + RDH12-KO-WT-R<br>*Rdh12* KO allele PCR:<br>RDH12-KO-Shared-F + RDH12-KO-Knockout-R	*Rdh12* WT allele PCR:<br>1. 94°C for 2 min<br>2. Denature 94 °C for 30 s<br>3. Anneal 62 °C for 30 s<br>4. Extension 72 °C for 30 s<br>5. Final Extension 72 °C for 5 min<br>Store at 4 °C<br><br>Repeat Cycles 2-4 30X<br><br>*Rdh12* KO allele PCR:<br>1. 94 °C for 2 min<br>2. Denature 94 °C for 30 s<br>3. Anneal 60 °C for 30 s<br>4. Extension 72 °C for 30 s<br>5. Final Extension for 5 min<br>Store at 4 °C<br><br>Repeat Cycles 2-4 30X	*Rdh12* WT allele: 718 bp<br><br>*Rdh12* KO allele: 480 bp	25 µL reaction volume. 2.5 µL of 10 µM primers used.

Gnat1$^{-/-}$ Genotyping Reaction	<u>*Gnat1* PCR</u>:<br>GNAT1-KO-1 + GNAT1-KO-2 + GNAT1-KO-3 + GNAT1-KO-4	<u>*Gnat1* WT/ KO allele PCR</u>:<br>1. 98°C for 2 min<br>2. Denature 98 °C for 30 s<br>3. Anneal 55 °C for 30 s<br>4. Extension 72 °C for 75 s<br>5. Final Extension 72 °C for 7 min<br>Store at 4 °C<br><br>Repeat Cycles 2-4 34X	*Gnat1* WT allele: 300 bp<br>*Gnat1* KO allele: 200 bp	25 μL reaction volume. 2.5 μL of 10 μM primers used.
PR-*Rpe65* cDNA PCR	<u>PR-RPE65 coding region cDNA PCR</u>:<br>PR-RPE65-cDNA-Amp-F + PR-RPE65-cDNA-Amp-R	<u>PR-*Rpe65* Coding Region cDNA PCR</u>:<br>1. 98 °C for 1 min<br>2. Denature 98 °C for 10 s<br>3. Anneal 60 °C for 10 s<br>4. Extension 72 °C for 1 min<br>5. Final Extension 72 °C for 5 min<br>Store at 4 °C<br><br>Repeat Cycles 2-4 34X	*Rpe65* coding region amplicon:<br>1599 bp	50 μL reaction volume. 2.5 μL of 10 μM primers used. PhusionPlus Green master mix used.
LRAT coding region cDNA PCR	<u>*LRAT* coding region cDNA PCR</u>:<br>*LRAT*-5'-cDNA-code-amp-F + *LRAT*-3'-cDNA-code-amp-R	<u>*LRAT* coding region cDNA PCR:</u><br>1. 98 °C for 1 min<br>2. Denature 98 °C for 10 s<br>3. Anneal 60 °C for 10 s<br>4. Extension 72 °C for 30 s<br>5. Final Extension 72 °C for 5 min<br>Store at 4°C	*LRAT* coding region cDNA PCR amplicon:<br>709 bp	50 μL reaction volume. 2.5 μL of 10 μM primers used. PhusionPlus Green master mix used.

		Repeat Cycles 2-4 30X		
LRAT HiFi coding region cDNA PCR	<u>*LRAT* coding region cDNA PCR</u>:<br>*LRAT*-5'-cDNA-code-amp-HiFi-F + *LRAT*-3'-cDNA-code-amp-HiFi-R	<u>*LRAT* HiFi coding region cDNA PCR:</u><br>1. 98°C for 1 min<br>2. Denature 98 °C for 10 s<br>3. Anneal 60 °C for 10 s<br>4. Extension 72 °C for 30 s<br>5. Final Extension 72 °C for 5 min<br>Store at 4 °C<br><br>Repeat Cycles 2-4 30X	*LRAT* HiFi coding region cDNA PCR amplicon:<br>749 bp	50 µL reaction volume. 2.5 µL of 10 µM primers used. PhusionPlus Green master mix used.
pUC-19 Linear PCR for HiFi	<u>pUC19 HiFi PCR:</u><br>pUC19-HiFi-F + pUC19-HiFi-R	<u>pUC-19 Linear PCR for HiFi:</u><br>1. 98°C for 1 min<br>2. Denature 98 °C for 10 s<br>3. Anneal 60 °C for 10 s<br>4. Extension 72 °C for 1 min<br>5. Final Extension 72 °C for 5 min<br>Store at 4 °C<br><br>Repeat Cycles 2-4 30X	pUC-19 Linear PCR amplicon for HiFi:<br>2686 bp	50 µL reaction volume. 2.5 µL of 10 µM primers used. PhusionPlus Green master mix used.
RPE65 coding region cDNA PCR	<u>*RPE65* coding region cDNA PCR</u>:<br>*RPE65*-5'-cDNA-code-amp-F + *RPE65*-5'-cDNA-code-amp-R	<u>*RPE65* coding region cDNA PCR:</u><br>1. 98 °C for 1 min<br>2. Denature 98 °C for 10 s<br>3. Anneal 60 °C for 10 s<br>4. Extension 72 °C for 1 min	*RPE65* coding region cDNA PCR amplicon:<br>1617 bp	50 µL reaction volume. 2.5 µL of 10 µM primers used. PhusionPlus Green master mix used.

		5. Final Extension 72 °C for 5 min<br>Store at 4 °C<br><br>Repeat Cycles 2-4 30X		
GAPDH coding region cDNA PCR	<u>*GAPDH* coding region cDNA PCR:</u><br>*GAPDH*-5'-cDNA-code-amp-F + *GAPDH*-5'-cDNA-code-amp-R	<u>*GAPDH* coding region cDNA PCR:</u><br>1. 98°C for 1 min<br>2. Denature 98 °C for 10 s<br>3. Anneal 60 °C for 10 s<br>4. Extension 72 °C for 1 min<br>5. Final Extension 72 °C for 5 min<br>Store at 4 °C<br><br>Repeat Cycles 2-4 30X	*GAPDH* coding region cDNA PCR amplicon:<br>1042 bp	50 µL reaction volume. 2.5 µL of 10 µM primers used. PhusionPlus Green master mix used.
qSTAR *LRAT*, *RPE65*, *GAPDH* RT-qPCRs	Origene Primer Sets:<br>1. qSTAR-*LRAT*<br>2. qSTAR-*RPE65*<br>3. qSTAR-*GAPDH*	<u>qSTAR RT-qPCR reactions:</u><br>1. 50.0 °C for 2 min<br>2. 50.0 °C for 2 min<br>3. 95.0 °C for 2 min<br>4. 95.0 °C for 15 s<br>5. 60.0 °C for 1 min<br>6. Image<br>Repeat steps 2-6 35x	N/A	10 µL reaction volume. 1 µL of 10 µM primer sets used.

[a] PCR and qPCR thermocycler conditions, reaction setup, expected amplicon sizes, and primer combinations are listed; other details are described under Material and Methods.

Table S3. List of primers used for genotyping mice and for amplifying mouse RPE65 cDNA templates.[a]

Primer	*Sequences (5' to 3')*
PR-LRAT/RPE65-Geno1-F	GATCCTGTTAAGAAGGGATTAGCA
PR-LRAT/RPE65-Geno2-F	TCGACTGTGCCTTCTAGTTGCC
PR-LRAT/RPE65-Geno3-R	CCATCCTCTGCCCACCTTGT
Rd8-F1	GTGAAGACAGCTACAGTTCTGATC
Rd8-F2	GCCCCTGTTTGCATGGAGGAAACTTGGAAGACAGCTACA GTTCTTCTG
Rd8-R	GCCCCATTTGCACACTGATGAC
Rd1-Common	CATGTCCTACAGCCCCTCTC
Rd1-Mutant	AAGCTAGCTGCAGTAACGCCATTT
Rd1-WT	ACCATTTGCAAGGAAAGCAC
FLP-Check-F	ACAGAGACAAAGACAAGCGTTAGTAGG
FLP-Check-R	ATTTCCCACAACATTAGTCAACTCCGTTAGG
LRAT-KO-Shared	TCCAGTTCCAGACTCTTTCCACCCAC
LRAT-KO-Knockout	TGCGAGGCCAGAGGCCACTTGTGTAGC
LRAT-KO-WT	AAGTGCTGGGCATGGTGACTTGTG
RDH12-KO-Shared-F	GCTGAGCCACTTTCCCTGCCCT
RDH12-KO-WT-R	AGAGCCGCCAGAGCACAGCCT
RDH12-KO-Knockout-R	GCCCCGACTCATCTGCGTGTT
GNAT1-KO-1	GAGGATTGGGAAGACAATAGCAG

GNAT1-KO-2	CACCAGCACCATGTCGTAAG
GNAT1-KO-3	CGAGTTCATTGCCATCATCTACG
GNAT1-KO-4	ATACCCGAGTCCTTCCACAAGC
PR-RPE65-cDNA-Amp-F	ATGTCTATCCAAATTGAACACCCTGCT
PR-RPE65-cDNA-Amp-R	TCACAGGGATATTAGTCTCCACTTCAGC

[a] The primers were used for the amplification of the corresponding DNA regions for genotyping, sequencing, and transcription analysis, as described in Materials and Methods. The sequences were designed using Primer-BLAST tool.(30)

Table S4. Primers used for generating AAV-*Lrat* and AAV-tdT constructs.[a]

Primer	*Sequences (5' to 3')*
AAV-hSyn-F	TAGGAATTCGATATCAAGCTTATCG
AAV-hSyn-R	CATGGTGGCGGATCCG
AAV-LRAT-F	CGGATCCGCCACCATGAAGAACCCAATGCTGGAAGC
AAV-LRAT-R	AGCTTGATATCGAATTCCTAGCCAGACATCATCCACAAG
HF-AAV-hSyn-LRAT-F	GAATTCGATATCAAGCTTATCG
HF-AAV-hSyn-LRAT-R	CTAGCCAGACATCATCCAC
HF-IRES-F	TGTGGATGATGTCTGGCTAGCTCGAGCTTATTCCAGATG
HF-IRES-R	TGCTCACCATCATGTTATTATCATCGTGTTTTTC
HF-tdT-F	TAATAACATGATGGTGAGCAAGGGAGAG
HF-tdT-R	ATAAGCTTGATATCGAATTCTTACTTGTACAGCTCGTCC
AAV-GFAP-F	GATGTCTGGCTAGCCAGCTTATCGATAATCAACC
AAV-GFAP-R	TTGGGTTCTTCATGGTGGCGAATTCCCG
HF-AAV-GFAP-LRAT-F	CGCCACCATGAAGAACCCAATGCTGGAAG
HF-AAV-GFAP-LRAT-R	AAGCTGGCTAGCCAGACATCATCCACAAG

[a] These primers were used for cloning of AAV-*Lrat* constructs for transduction of Müller glia and retinal ganglion cells. Generation of AAVs is described in Materials and Methods.

SUPPLEMENTAL FIGURES

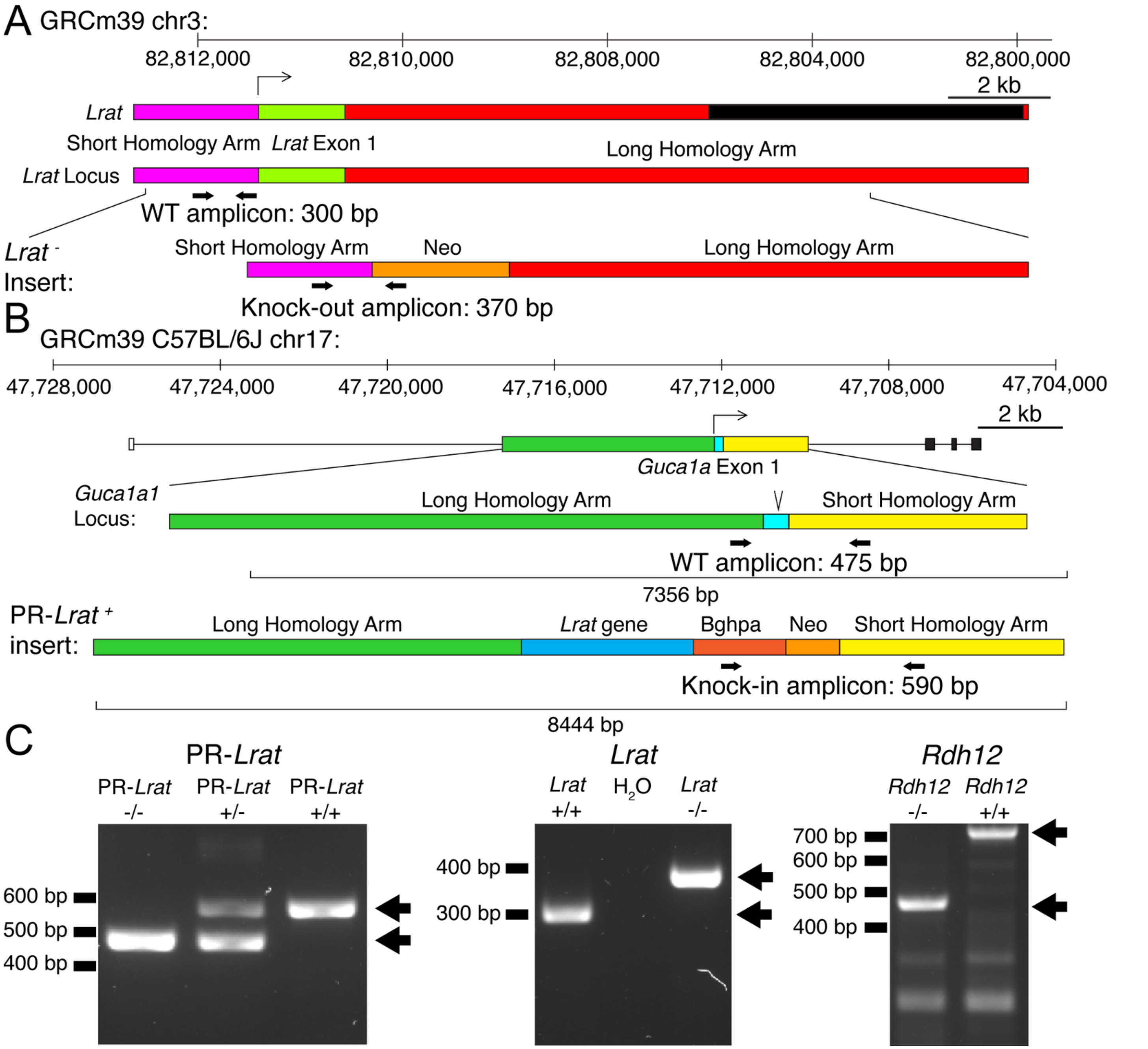

Supplemental Figure 1. Map of *Lrat* and *Guca1a1* genomic loci alongside the respective *Lrat* knock-out and *Lrat* knock-in recombination cassettes used to generate *Lrat*$^{-/-}$ and PR-*Lrat*$^{+}$ (*Gcap1*^{Lrat}) mice. A. Map of the murine *Lrat* genomic locus and the recombination cassette used to generate *Lrat*$^{-/-}$ mice. Primer annealing sites for genotyping WT and *Lrat* knock-out mice are mapped using black arrows. **B.** Map of the murine *Guca1a1* (syn. *Gcap1*) genomic locus and the recombination cassette used to generate PR-*Lrat*$^{+}$ (*Gcap1*^{Lrat}) mice. Primer annealing sites for genotyping WT and *Lrat* knock-in mice are mapped using black arrows. **C.** Representative genotyping PCR amplicons for *Lrat* knock-in (PR-*Lrat*$^{+}$/ *Gcap1*^{Lrat}), *Lrat* knock-out (*Lrat*$^{-/-}$), and *Rdh12* knock-out (*Rdh12*$^{-/-}$) mouse lines, with expected products highlighted for each reaction using black arrows. Molecular mass markers are shown to the left.

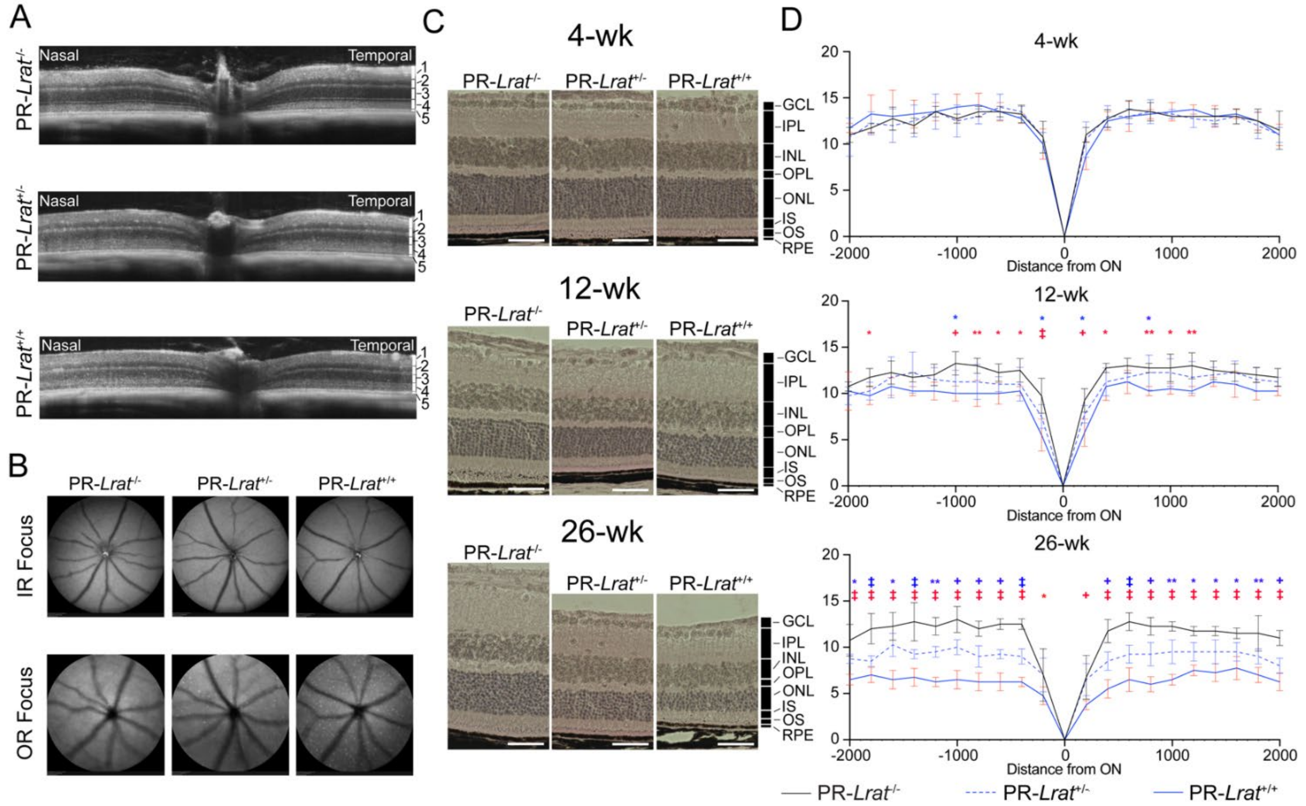

Supplemental Figure 2. Baseline phenotyping and natural history of PR-*Lrat*$^{+/-}$ and PR-*Lrat*$^{+/+}$ mice relative to age-matched PR-*Lrat*$^{-/-}$ (WT) controls. A. Representative OCT tomographs from 6-week-old PR-*Lrat*$^{-/-}$ (WT) and PR-*Lrat*$^{+}$ mice. Layers of the retina are labeled 1 to 5: 1. GCL and IPL; 2. INL and OPL; 3. ONL; 4. PR-IS and OS; 5. RPE. **B.** Representative SLO images focused on the inner retinal (IR) layers and outer retinal (OR) layers taken from 6-week-old WT and PR-*Lrat*$^{+}$ mice. Each SLO image was taken from the same eyes selected for representative OCT imaging. Representative OCTs and SLOs were selected after comparing 3 independent animals per genotype. **C.** Representative H&E staining of fixed cryosections from 4-week-old, 12-week-old, and 26-week-old PR-*Lrat*$^{+/-}$ and PR-*Lrat*$^{+/+}$ mice, with age-matched PR-*Lrat*$^{-/-}$ (WT) controls. Representative section images were taken within ~400 µm from the optic nerve (selected from n = 4 per genotype). **D.** Spider plots of mean PR nuclei counts from 4-week-old, 12-week-old, and 26-week-old WT and PR-*Lrat*$^{+}$ mice, taken at 200 µm intervals from the inferior (-2000 µm) to the superior (2000 µm) of each sampled cryosection (n = 4). SDs are provided for each averaged point per genotype. Results of statistically significant Tukey's multiple comparisons tests between the PR-*Lrat*$^{+}$ genotypes and WT controls following two-way ANOVA are provided above each point (* = $P \leq 0.05$; ** = $P \leq 0.01$; + = $P \leq 0.001$; ‡ = $P \leq 0.0001$). Statistics and SD bars are given in blue for PR-*Lrat*$^{+/-}$ mice and in red for PR-*Lrat*$^{+/+}$ mice. A two-way ANOVA analysis indicated no statistically significant variations in WT control and PR-*Lrat*$^{+}$ nuclei counts at 4-weeks of age, but statistically significant changes emerged at the 12- and 26-week time points. By 26-weeks of age, on average there were ~2.6 fewer nuclei per column at each point in cryosections from PR-*Lrat*$^{+/-}$ mice compared to age-matched WT controls, and ~5.2 fewer nuclei per column for PR-*Lrat*$^{+/+}$ mice.

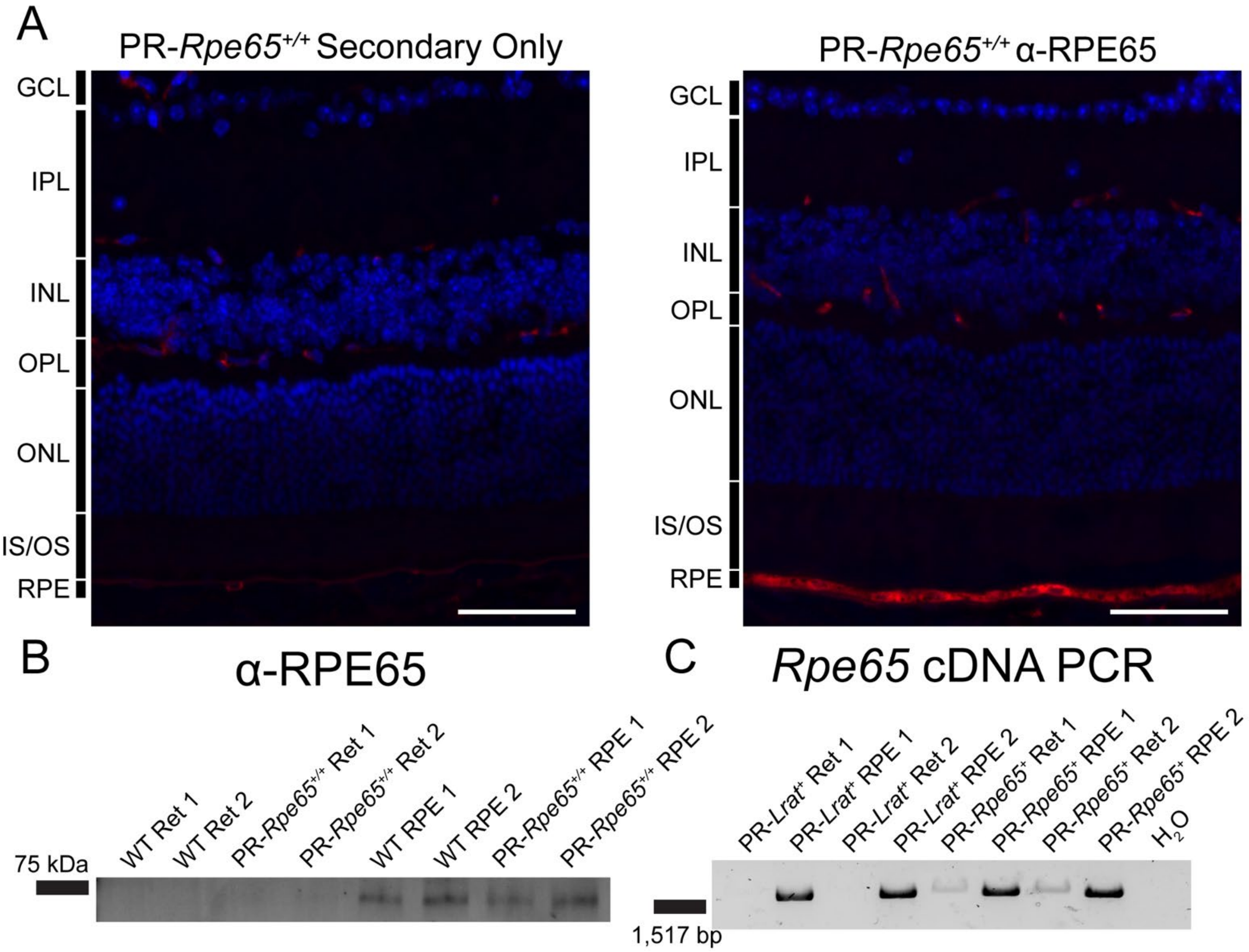

Supplemental Figure 3. RPE65 expression in PR-*Rpe65*$^{+/+}$ mice. A. Representative immunofluorescence staining of RPE65 (red signal) in PFA-fixed PR-*Rpe65*$^{+/+}$ retinal cryosections, with a secondary-only control on the left and anti-RPE65 stained section on the right. DAPI counterstaining is featured in blue; scale bars = 50 µm. Retina layers are labeled to the left of each image. **B.** Anti-RPE65 Western blotting of dissected neural retina (Ret) and RPE/choroid (RPE) homogenates from wild-type (WT) and PR-*Rpe65*$^{+/+}$ mice (n = 2 per genotype). A molecular mass reference ladder is included to the left. **C.** SYBR Safe-stained agarose gel image of PCR amplicons of the RPE65 coding region (expected size: 1,617 bp) run on a 1.75% agarose gel, using cDNA templates synthesized from neural retina (Ret) and RPE/choroid (RPE) bulk mRNA extracts from PR-*Lrat*$^{+/-}$ (negative control) and PR-*Rpe65*$^{+/+}$ mice. PCRs were conducted using custom primers designed to amplify the RPE65 coding region from each cDNA template (n = 2 per genotype). A molecular mass reference ladder is included to the left.

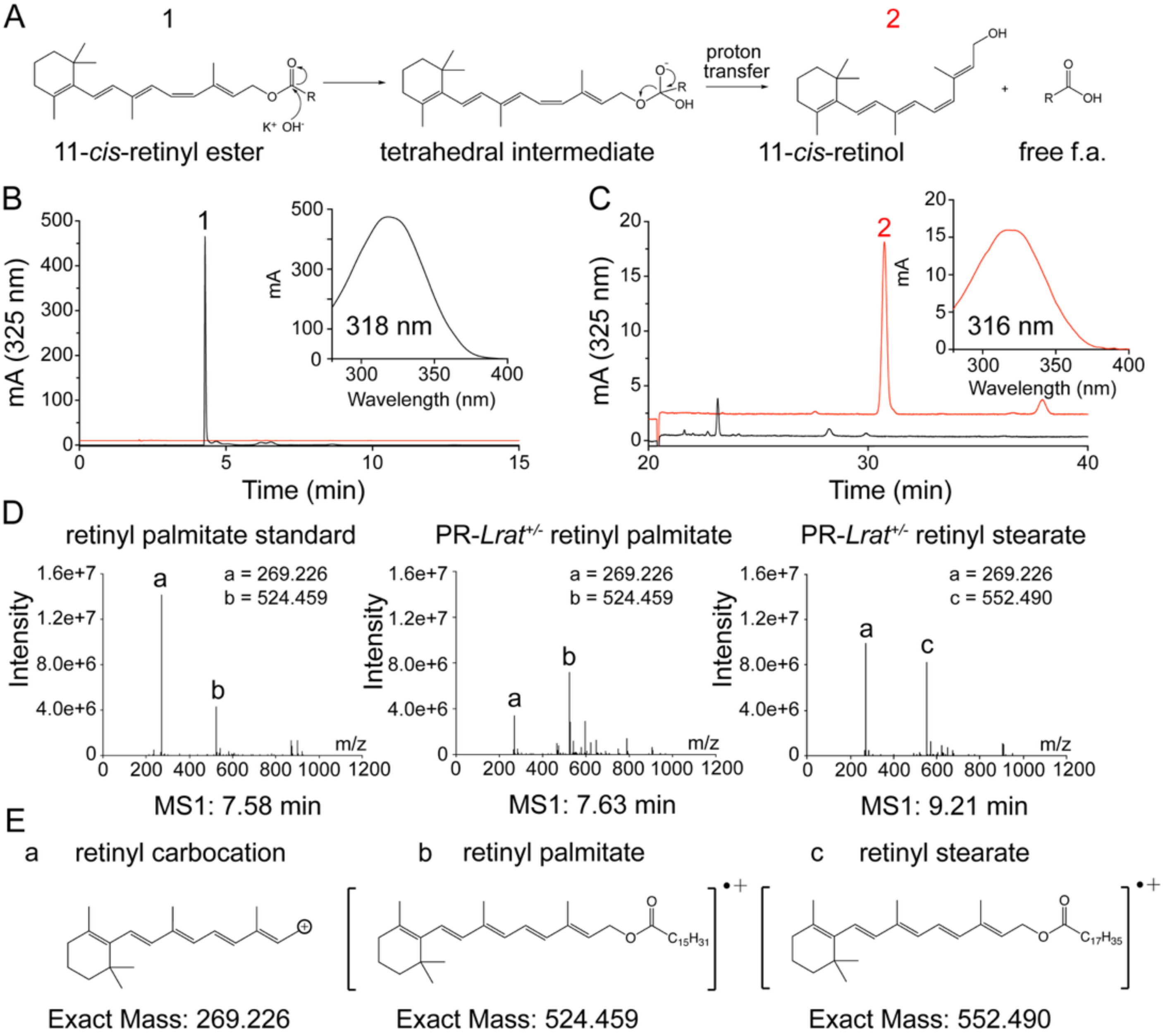

Supplemental Figure 4. Predominant retinyl ester species in whole-eye homogenates from PR-*Lrat*$^{+/-}$ mice. A. Reaction scheme for alkaline (KOH) hydrolysis to release retinol from the predominant RE species isolated *via* normal-phase HPLC of whole-eye extracts from dark-adapted PR-*Lrat*$^{+/-}$ mice. 11-*cis*-RE and free 11-*cis*-retinol structures are labeled 1 and 2 (R = variable fatty acid moiety). **B.** Pre-hydrolysis (black) and post-hydrolysis (red) normal-phase HPLC traces focused on the RE elution timeframe. The predominant 11-*cis*-RE peak is labeled “1.” An inset with the absorbance spectrum of peak “1” is included. **C.** Pre-hydrolysis (in black) and post-hydrolysis (in red) normal-phase HPLC traces focused on the retinol elution timeframe, with the post-hydrolysis 11-*cis*-retinol peak labeled “2.” An inset with the absorbance spectrum of peak “2” is included. **D.** MS1 precursor-ion spectra of a 100-pmol retinyl palmitate standard, and extracted retinyl palmitate, and extracted retinyl stearate from PR-*Lrat*$^{+/-}$ mice. Peak masses are included as insets and reverse-phase HPLC elution times are provided beneath each RE-species spectrum. **E.** Structures and masses of the retinoid parent ions. For panels **D-E**, MS1 peaks are labeled for retinyl carbocation (a), retinyl palmitate (b), and retinyl stearate (c).

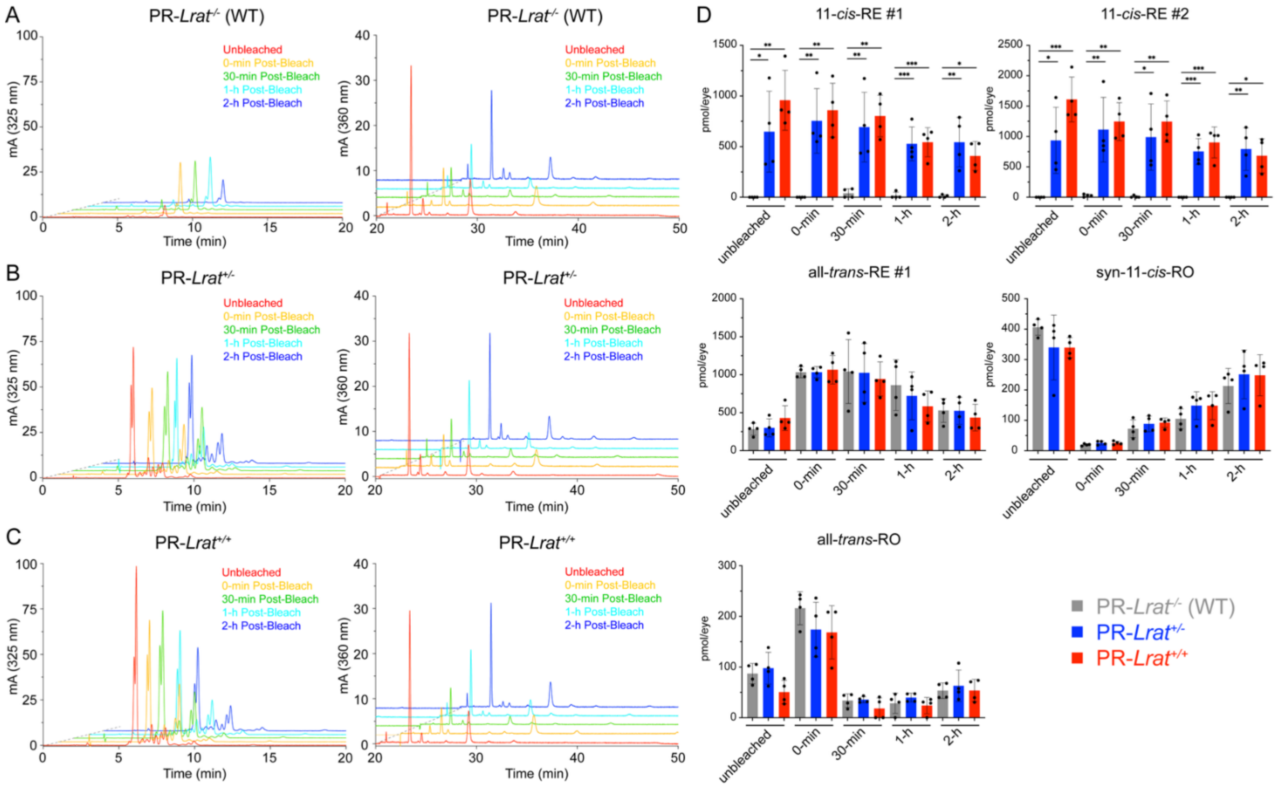

Supplemental Figure 5. Comparison of visual cycle kinetics and retinoid content of 8-week-old PR-*Lrat*$^{-/-}$, PR-*Lrat*$^{+/-}$, and PR-*Lrat*$^{+/+}$ mice, pre- and post-bleaching. A. Representative HPLC traces of whole-eye retinoid extracts from PR-*Lrat*$^{-/-}$ (WT) mice; samples were prepared from DA mice pre-bleaching with light (unbleached), 0-min post-bleach, 30-min post-bleach, 1-h post-bleach, and 2-h post-bleach, with a focus on the RE elution timeframe and RO elution timeframe in separate plots. Representative plots were selected from n = 4 per genotype. Traces are offset and ordered from unbleached to 2-h post-bleach. A dashed gray line indicates angle of offset. **B.** Representative HPLC traces of whole-eye retinoid extracts from PR-*Lrat*$^{+/-}$ mice; samples were prepared from DA mice pre-bleaching (unbleached), 0-min post-bleach, 30-min post-bleach, 1-h post-bleach, and 2-h post-bleach, with a focus on the RE elution timeframe and RO elution timeframe in separate plots. Representative plots were selected from n = 4 per genotype. Traces are offset and ordered from unbleached to 2-h post-bleach. A dashed gray line indicates angle of offset. **C.** Representative HPLC traces of whole-eye retinoid extracts from PR-*Lrat*$^{+/+}$ mice; samples were prepared from DA mice pre-bleaching (unbleached), 0-min post-bleach, 30-min post-bleach, 1-h post-bleach, and 2-h post-bleach, with a focus on the RE elution timeframe and RO elution timeframe in separate plots. Representative plots were selected from n = 4 per genotype. Traces are offset and ordered from unbleached to 2-h post-bleach. A dashed gray line indicates the angle of offset. **D.** Quantification of 11-*cis*-RE #1, 11-*cis*-RE #2, all-*trans*-RE #1, *syn*-11-*cis*-RO, and all-*trans*-RO levels for each genotype, per timepoint (n = 4). Statistically significant results of *post hoc* Tukey's multiple comparisons tests (timepoint-matched comparisons) performed after two-way ANOVAs are featured on each plot (* = *P* ≤ 0.05; ** = *P* ≤ 0.01; *** = *P* ≤ 0.001; **** = *P* ≤ 0.0001). Bars are plotted as means ± SD.

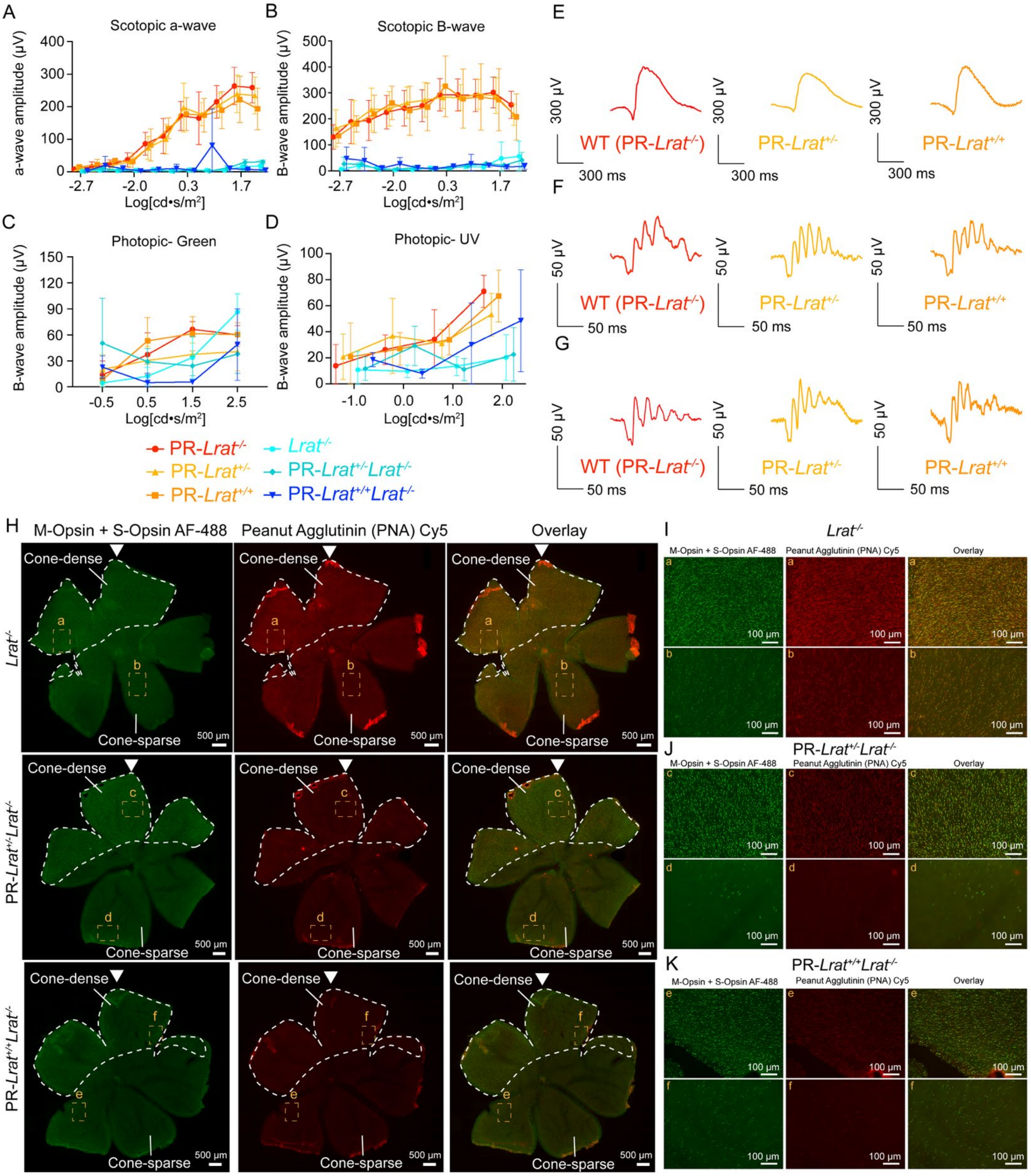

Supplemental Figure 6. In vivo baseline scotopic (rod-derived) and photopic (cone-derived) photoresponses of PR-*Lrat*$^{+}$ and PR-*Lrat*$^{+}$*Lrat*$^{-/-}$ mice, and immunohistochemical analysis of neural retina

wholemounts from 12-week-old mice. Baseline electroretinography (ERG) is shown for WT (PR-*Lrat*$^{-/-}$), PR-*Lrat*$^{+}$, *Lrat*$^{-/-}$, and PR-*Lrat*$^{+}$*Lrat*$^{-/-}$ mouse strains, with quantification of scotopic a-wave **(A)** and b-wave amplitudes **(B)** collected at 6-7 weeks of age. Quantification of cone-driven b-wave photopic response amplitudes, collected at 6-7 weeks of age, in response to green **(C)** and UV **(D)** flashes. Scotopic, rod-driven responses to light stimulus of −2.0 log[cd • s/m^{-2}], ERG waveforms, are shown in Panel **E** for the various mouse strains. Photopic, cone-driven ERG waveforms upon green light stimulus of +2.5 log[cd • s/m^{-2}] are shown in panel **F** and upon UV light stimulus of +2.0 log[cd • s/m^{-2}] in panel **G**. The colors are consistent with panels **A-D**. Data are presented as mean ± SEM (n = 4). **H.** Full-field, stitched images of neural retina wholemounts from 12-week-old *Lrat*$^{-/-}$, PR-*Lrat*$^{+/-}$*Lrat*$^{-/-}$, and PR-*Lrat*$^{+/+}$*Lrat*$^{-/-}$ mice, divided into the M-opsin + S-opsin channel (combined staining in green), PNA channel (red), and superimposed overlays (green and red channels). The superior side of each retinal wholemount is marked with a solid white triangle, and cone-dense regions are outlined with a dashed white line; enlarged images from cone-sparse and cone-dense regions in panels **I**, **J**, and **K** are marked with a dashed orange boundary. Representative neural retina wholemount images were selected from n = 3-4 wholemounts per genotype (scale bars = 500 μm). **I-K.** Enlarged images at 20x magnification display the cone-dense (labeled a, c, and e by genotype) and cone-sparse regions (labeled b, d, and f by genotype) of the representative neural retina wholemounts featured in panel **A** (scale bars = 100 μm).

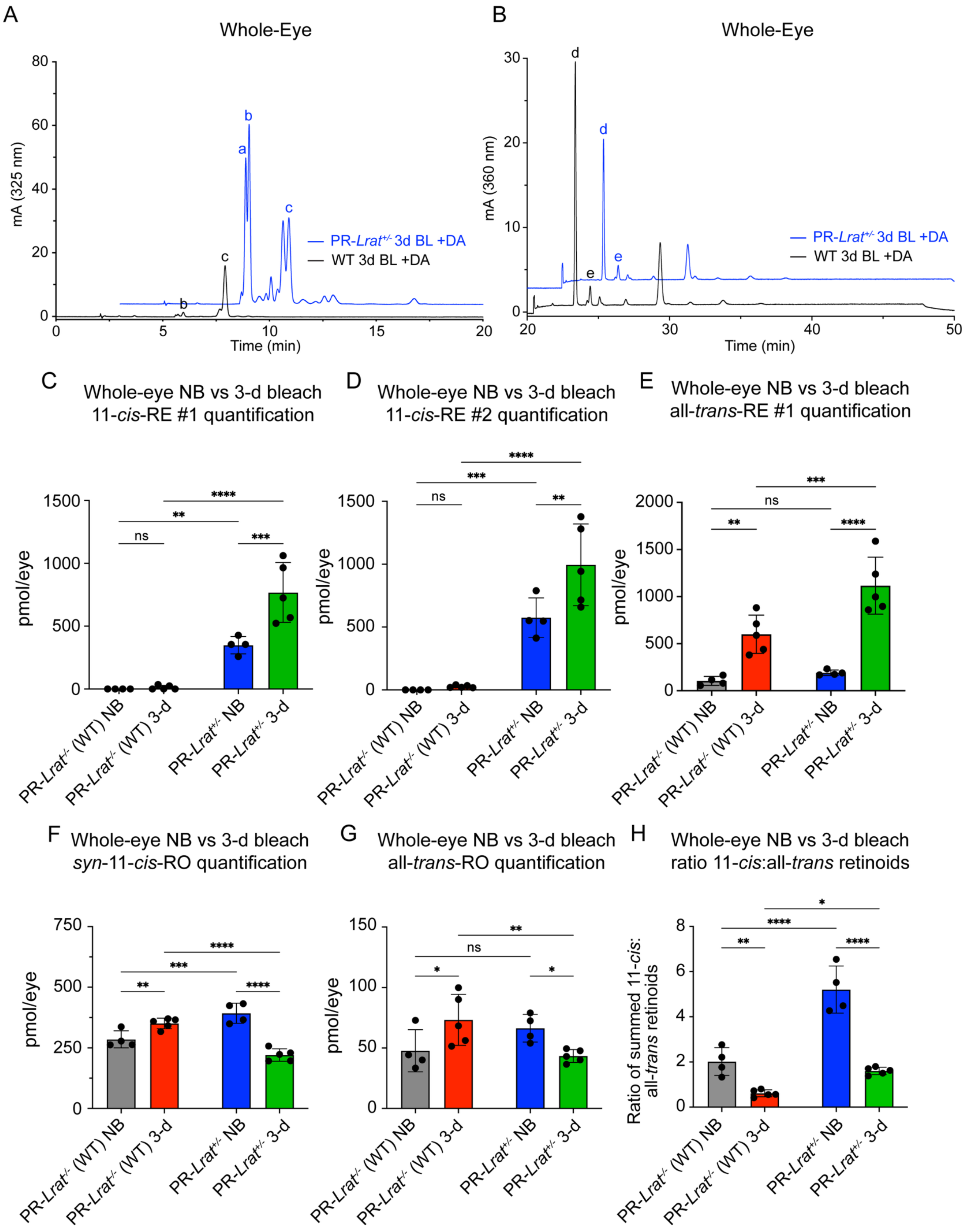
A
Whole-Eye
mA (325 nm)
Time (min)
PR-Lrat+/- 3d BL +DA
WT 3d BL +DA
a
b
c
B
Whole-Eye
mA (360 nm)
Time (min)
d
e
C
Whole-eye NB vs 3-d bleach
11-cis-RE #1 quantification
D
Whole-eye NB vs 3-d bleach
11-cis-RE #2 quantification
E
Whole-eye NB vs 3-d bleach
all-trans-RE #1 quantification
F
Whole-eye NB vs 3-d bleach
syn-11-cis-RO quantification
G
Whole-eye NB vs 3-d bleach
all-trans-RO quantification
H
Whole-eye NB vs 3-d bleach
ratio 11-cis:all-trans retinoids
pmol/eye
Ratio of summed 11-cis:
all-trans retinoids
PR-Lrat-/- (WT) NB
PR-Lrat-/- (WT) 3-d
PR-Lrat+/- NB
PR-Lrat+/- 3-d
ns

Supplemental Figure 7. Retinoid analyses of the whole mouse eyes after intense bleaches for 3 consecutive days. A. Representative HPLC traces, with a focus on the RE elution timeframe, of whole-eye retinoid extracts from 7-8-week WT mice (in black) and PR-*Lrat*$^{+/-}$ mice (in blue), bleached twice a day (5 min, 10,000 lux) for 3 consecutive days prior to 24 h of DA, harvest, and extraction. Peaks corresponding to 11-*cis*-RE #1, 11-*cis*-RE #2, and all-*trans*-RE #1 are labeled "a", "b", and "c," respectively. **B.** Representative HPLC traces, with a focus on the RO elution timeframe, of whole-eye retinoid extracts from 7-8-week WT mice (black) and PR-*Lrat*$^{+/-}$ mice (blue), bleached twice a day (5 min, 10,000 lux) for 3 consecutive days prior to 24 h of DA, harvest, and extraction. Peaks corresponding to *syn*-11-*cis*-RO and all-*trans*-RO peaks are labeled "d" and "e," respectively. **C-G.** Quantification and comparison of retinoid species in whole-eye extracts from DA unbleached (NB) and 3-day bleached (2x 5 min, 10,000 lux, 3 consecutive days) WT and PR-*Lrat*$^{+/-}$ mice (n = 4, unbleached; n = 5, 3-d bleached): 11-*cis*-RE #1 (**C**), 11-*cis*-RE #2 (**D**), all-*trans*-RE #1 (**E**), *syn*-11-*cis*-RO (**F**), and all-*trans*-RO (**G**). Results from uncorrected Fisher's LSD tests conducted after two-way ANOVAs are provided above the plot (ns = not significant, * = $P \leq 0.05$; ** = $P \leq 0.01$; *** = $P \leq 0.001$; **** = $P \leq 0.0001$). Whole-eye retinoid values for 6-7-week unbleached WT and PR-*Lrat*$^{+/-}$ mice are also used in **Figure 5**. **H.** Comparison of summed retinoid ratios (11-*cis* / all-*trans*) in whole eye extracts from unbleached and 3-d-post-bleach DA PR-*Lrat*$^{-/-}$ (WT) and PR-*Lrat*$^{+/-}$ mice (n = 4, unbleached; n = 5, 3-d bleached). Results from uncorrected Fisher's LSD tests conducted after a two-way ANOVA are provided above the plot (ns = not significant, * = $P \leq 0.05$; ** = $P \leq 0.01$; *** = $P \leq 0.001$; **** = $P \leq 0.0001$). Bars are plotted as means ± SD.

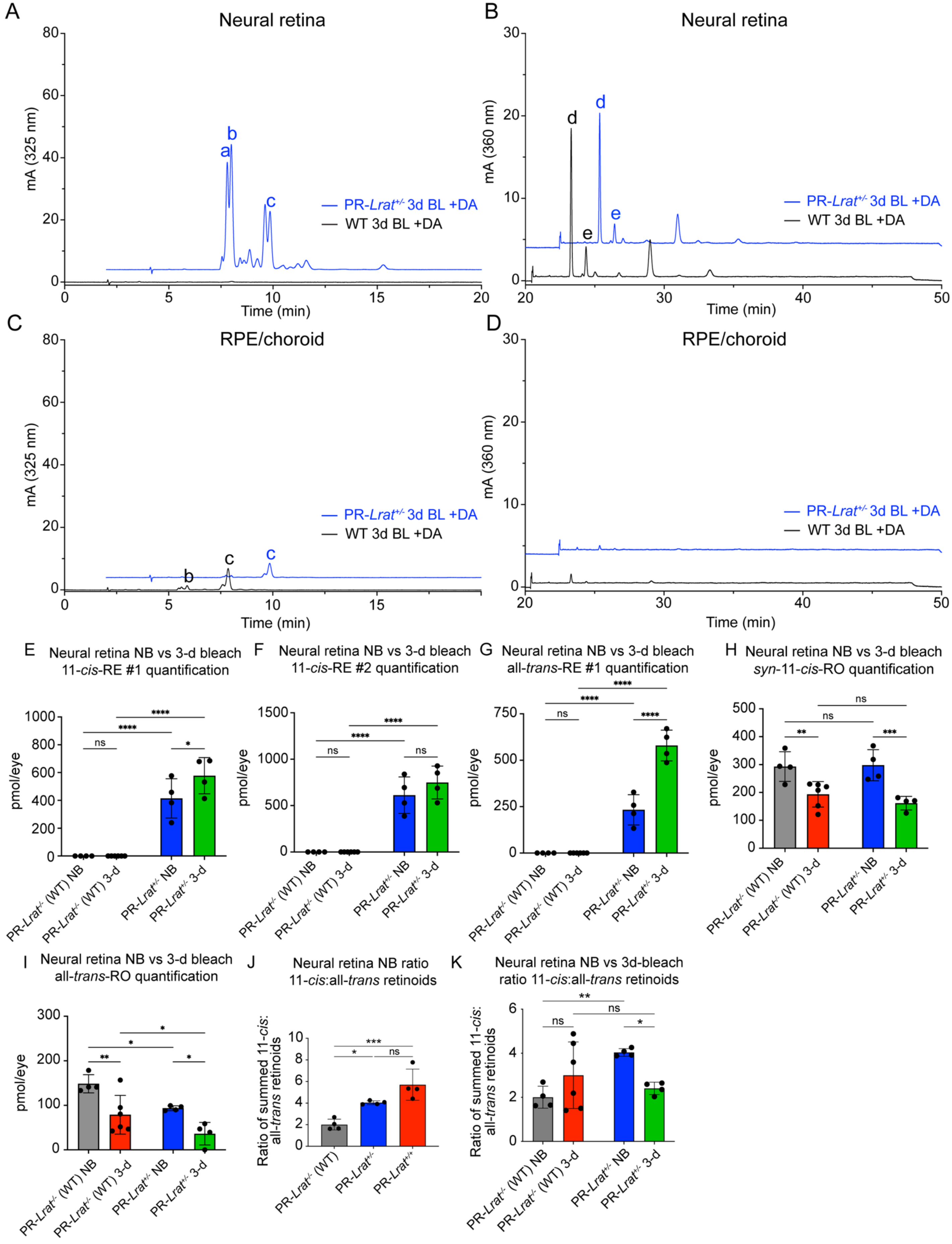
A
Neural retina
mA (325 nm)
Time (min)
a
b
c
PR-Lrat+/- 3d BL +DA
WT 3d BL +DA
B
Neural retina
mA (360 nm)
Time (min)
d
e
PR-Lrat+/- 3d BL +DA
WT 3d BL +DA
C
RPE/choroid
mA (325 nm)
Time (min)
b
c
PR-Lrat+/- 3d BL +DA
WT 3d BL +DA
D
RPE/choroid
mA (360 nm)
Time (min)
PR-Lrat+/- 3d BL +DA
WT 3d BL +DA
E
Neural retina NB vs 3-d bleach
11-cis-RE #1 quantification
pmol/eye
PR-Lrat-/- (WT) NB
PR-Lrat-/- (WT) 3-d
PR-Lrat+/- NB
PR-Lrat+/- 3-d
F
Neural retina NB vs 3-d bleach
11-cis-RE #2 quantification
pmol/eye
G
Neural retina NB vs 3-d bleach
all-trans-RE #1 quantification
pmol/eye
H
Neural retina NB vs 3-d bleach
syn-11-cis-RO quantification
pmol/eye
I
Neural retina NB vs 3-d bleach
all-trans-RO quantification
pmol/eye
J
Neural retina NB ratio
11-cis:all-trans retinoids
Ratio of summed 11-cis:
all-trans retinoids
PR-Lrat-/- (WT)
PR-Lrat+/-
PR-Lrat+/+
K
Neural retina NB vs 3d-bleach
ratio 11-cis:all-trans retinoids
Ratio of summed 11-cis:
all-trans retinoids

Supplemental Figure 8. Retinoid analyses of dissected neural retinas and RPE/choroid tissue, after intense bleaches for 3 consecutive days. A-B. Representative HPLC traces, with a focus on the RE elution timeframe (**A**) and RO elution timeframe (**B**), of retinoid extracts from dim-red-light-dissected neural retinas from 6-7-week WT mice (in black) and PR-*Lrat*$^{+/-}$ mice (in blue), bleached twice a day (5 min, 10,000 lux) for 3 consecutive days prior to 24 h of DA, harvest, and extraction. **C-D.** Representative HPLC traces, with a focus on the RE elution timeframe (**C**) and RO elution timeframe (**D**), of retinoid extracts from dissected RPE/choroid tissue from 6-7-week WT (in black) and PR-*Lrat*$^{+/-}$ (in blue) mice, bleached twice a day (5 min, 10,000 lux) for 3 consecutive days prior to 24 h of dark-adaptation, harvest, and extraction. For panels A-D, peaks corresponding to 11-*cis*-RE #1, 11-*cis*-RE #2, and all-*trans*-RE are labeled "a", "b", and "c," respectively; peaks corresponding to *syn*-11-*cis*-RO and all-*trans*-RO peaks are labeled "d" and "e," respectively. **E-I.** Quantification and comparison of retinoid species in dim-red-light-dissected neural retina extracts from DA unbleached (NB) and 3-day bleached (2x 5 min, 10,000 lux, 3 consecutive days) mice (n = 4, PR-*Lrat*$^{+/-}$ unbleached/ bleached + WT unbleached; n = 6, WT bleached): 11-*cis*-RE #1 (**E**), 11-*cis*-RE #2 (**F**), all-*trans*-RE #1 (**G**), *syn*-11-*cis*-RO (**H**), and all-*trans*-RO (**I**). Results from uncorrected Fisher's LSD tests conducted after two-way ANOVAs are provided above the plot (ns = not significant, * = $P \leq 0.05$; ** = $P \leq 0.01$; *** = $P \leq 0.001$; **** = $P \leq 0.0001$). **J.** Comparison of summed retinoid ratios (11-*cis* / all-*trans*) in neural retina extracts from unbleached DA mice (n = 4). Results from Tukey's multiple comparisons tests conducted after one-way ANOVA are provided above the plot (ns = not significant, * = $P \leq 0.05$; ** = $P \leq 0.01$; *** = $P \leq 0.001$; **** = $P \leq 0.0001$). **K.** Comparison of summed retinoid ratios (11-*cis* / all-*trans*) in neural retina extracts from unbleached and 3-day-bleached and DA mice (n ≥ 4). Results from uncorrected Fisher's LSD tests conducted after a two-way ANOVA are provided above the plot (ns = not significant, * = $P \leq 0.05$; ** = $P \leq 0.01$; *** = $P \leq 0.001$; **** = $P \leq 0.0001$). Data bars are plotted as mean ± SD.

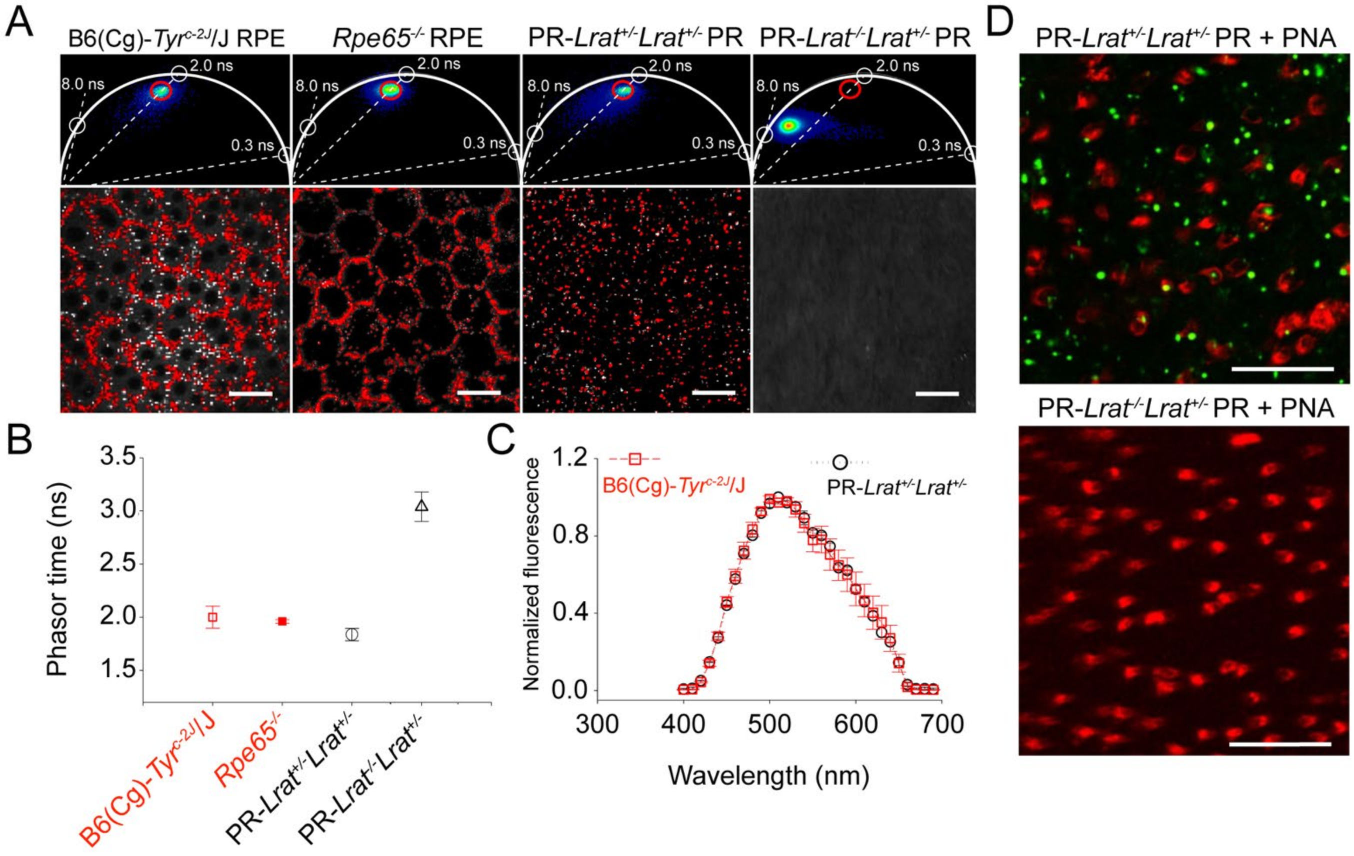

Supplemental Figure 9. Retinosomes in PR-*Lrat*⁺ mice are evenly distributed across rods and cones. A. Phasor plots of fluorescence lifetimes for the RPE layer in eyecups from albino WT (B6(Cg)*Tyr*$^{c\text{-}2J}$/J) and *Rpe65*$^{-/-}$ mice are positioned alongside those for the PR layer in eyecups from PR-*Lrat*$^{+/-}$*Lrat*$^{+/-}$ and PR-*Lrat*$^{-/-}$*Lrat*$^{+/-}$ mice. Arbitrary red color was assigned to image pixels corresponding to phasor points encircled in red. Corresponding fluorescence lifetime images (FLIM) are shown below each phasor plot; time scales are the same previously published (31). Scale = 25 µm. **B.** Phasor times for PR layers in eyecups from WT (B6(Cg)*Tyr*$^{c\text{-}2J}$/J) RPE, *Rpe65*$^{-/-}$ RPE, PR-*Lrat*$^{+/-}$*Lrat*$^{+/-}$, and PR-*Lrat*$^{-/-}$*Lrat*$^{+/-}$ mice. Error bars represent SD. **C.** Two-photon excitation spectra for the RPE layer of WT (B6(Cg)*Tyr*$^{c\text{-}2J}$/J) mice, and PR layer of PR-*Lrat*$^{+/-}$*Lrat*$^{+/-}$ mice. **D.** Representative images are shown of the PR-IS in intact mouse eyes of PR-*Lrat*$^{+/-}$*Lrat*$^{+/-}$ mice (upper panel), and PR-*Lrat*$^{-/-}$*Lrat*$^{+/-}$ mice (lower panel), 2 days after subretinal (SR) injection with PNA-rhodamine. An arbitrary color scheme was selected according to the emission spectral detector setting. Green color was assigned to fluorescence collected in the range 430-530 nm, corresponding to PR retinosome-like structures; and red color was assigned to fluorescence collected in the range 600-720 nm, corresponding to rhodamine. Scale = 25 µm; n = 3 per genotype.

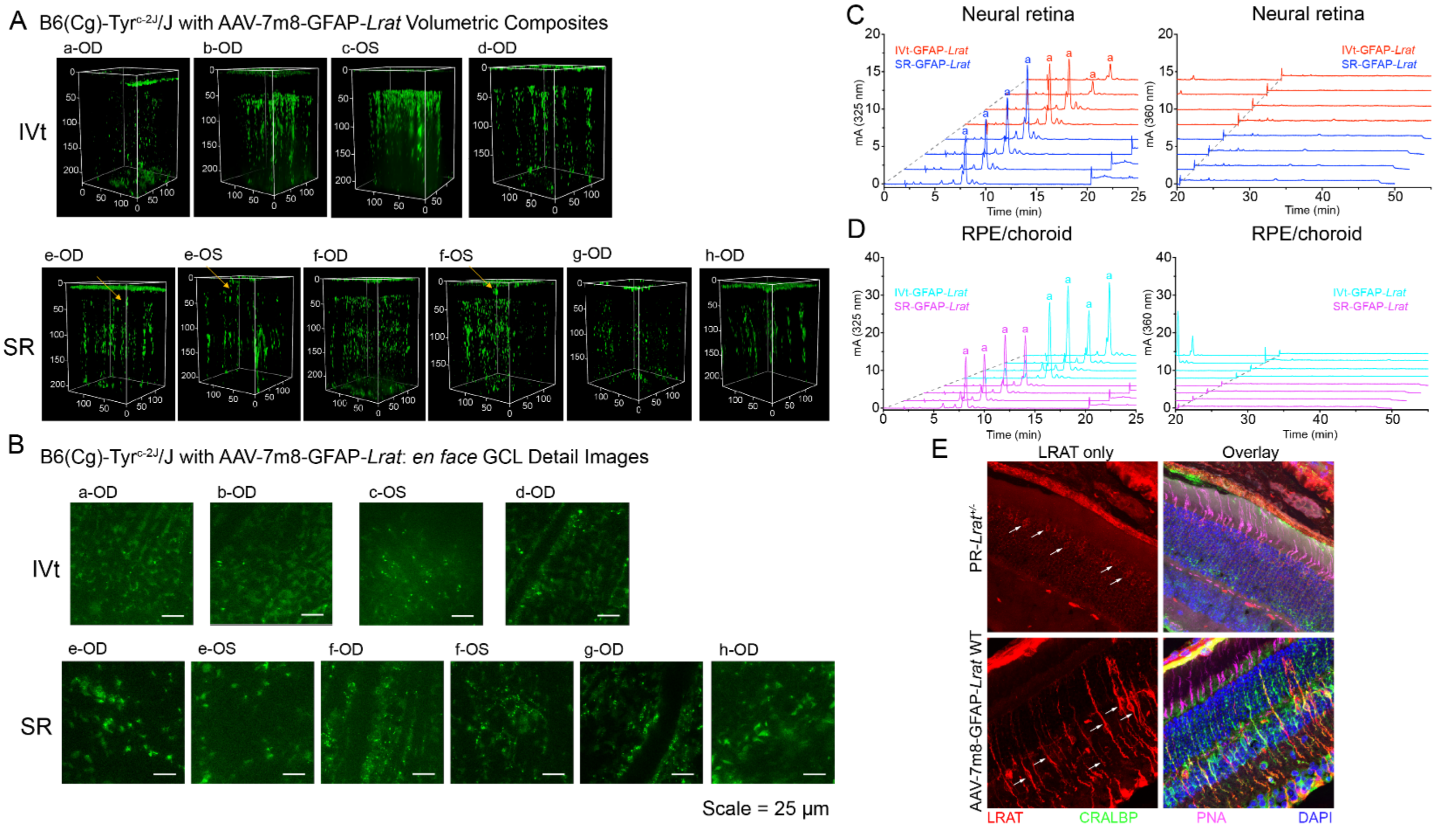

Supplemental Figure 10. Compiled two-photon retinoid analysis, and immunofluorescent phenotyping of neural retinas from WT mice, 3-weeks after subretinal (SR) or intravitreal (IVt) injection with AAV-7m8-GFAP-*Lrat*. A. 3-dimensional volumetric reconstructions of retinosome-like structures spanning MG cell bodies in the eyes from WT mice, 3-weeks post-injection (IVt, n = 4; or SR, n = 6) with AAV-7m8-GFAP-*Lrat*. Orange arrows indicate microglia/macrophages traversing the PR-IS and OS; scale bars are in µm. **B.** En face detail images of retinosome-like fluorescent signal in the GCL of eyes from WT mice, 3-weeks post-injection (IVt, n = 4; or SR, n = 6) with AAV-7m8-GFAP-*Lrat*. **C.** Compiled retinoid traces of neural-retina extracts from SR- and IVt-injected AAV-7m8 GFAP-*Lrat*-treated WT mice, post-TP-imaging (light-adapted). Individual panels correspond to the RE elution (left) and RO elution (right) timeframes. Peaks corresponding to all-*trans*-RE #1 are labeled “a.” Individual traces from SR-injected animals (blue traces) and IVt-injected animals (red traces) are staggered for ease of comparison (n = 4 per treatment group). Dashed gray lines indicate the angle of offset. **D.** Compiled retinoid traces for RPE/choroid extracts from SR- and IVt-injected AAV-7m8-GFAP-*Lrat*-treated WT mice, post-TP-imaging (light-adapted). Panels are formatted to match traces featured in panel **C**. **E.** Confocal imaging of cryosections from PR-*Lrat*$^{+/-}$ and WT mice, 3-weeks post-IVt-injection with AAV-7m8-GFAP-*Lrat* stained with antibodies against LRAT and CRALBP along with PNA and DAPI. The left-hand panels show the LRAT signal only, and the right panels show an overlay of the signals in all channels. Regions of transgenic LRAT signal are highlighted with white arrows for the PR-*Lrat*$^{+/-}$ mice (indicating PR-localized LRAT expression) and WT mice, treated with AAV-7m8-GFAP-*Lrat* (indicating MG-localized LRAT expression). For the panel focusing on the WT mouse treated with AAV-7m8-GFAP-*Lrat*, the image was taken at a region of Müller glial

transduction; regions of MG transduction were scattered in the cryosections sampled with confocal microscopy (n = 4).

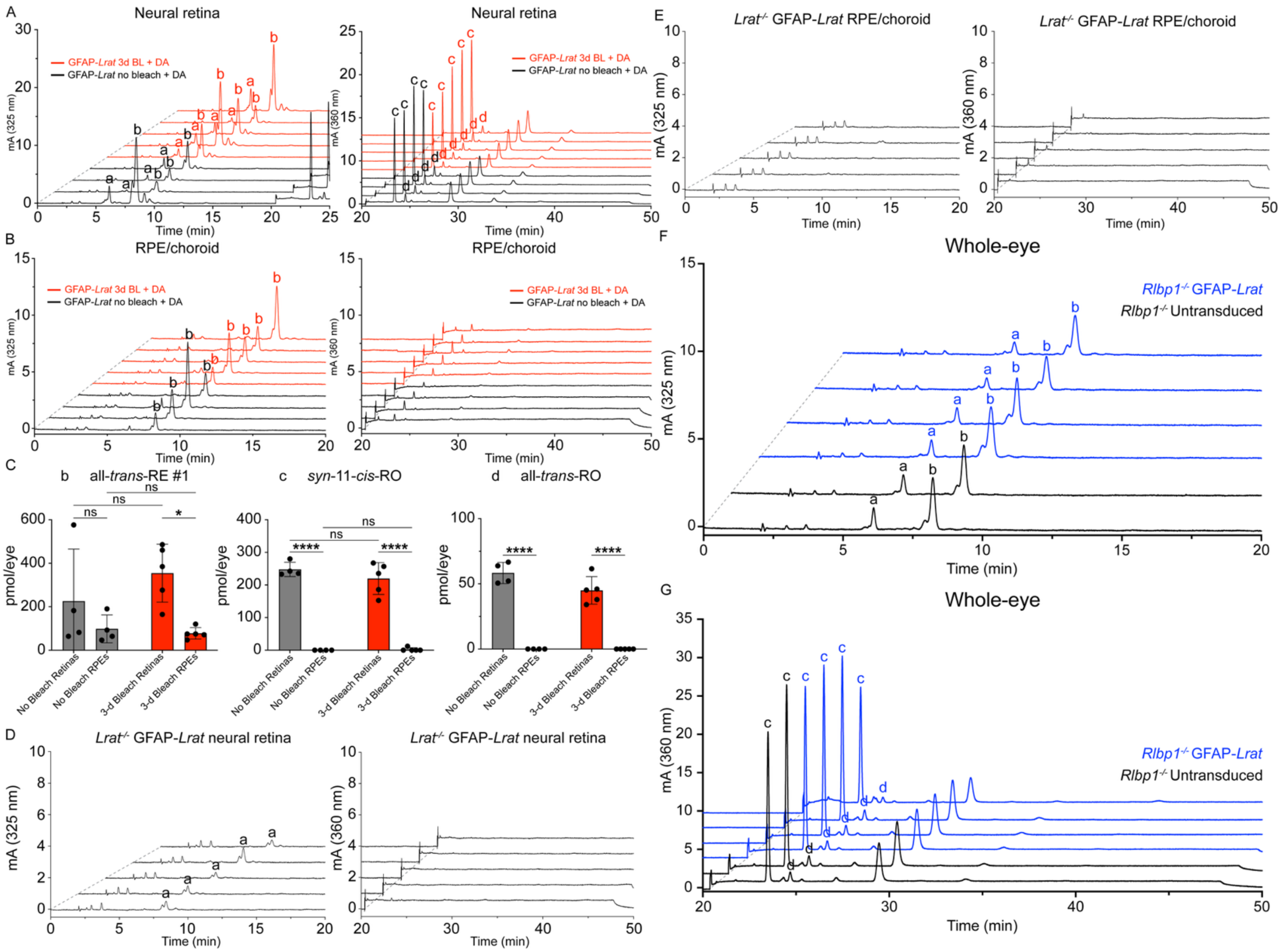

Supplemental Figure 11. Effects of successive bleaching, genetic ablation of *Lrat*, and genetic ablation of *Rlbp1* on retinyl ester accumulation in Müller glia transduced with *Lrat*. A. Staggered traces for samples of neural retina extracts from unbleached (black traces, n = 4) and post-3d-bleaching (red traces, n = 5) DA WT mice, 4-weeks-post-IVt injection with AAV7m8-GFAP-*Lrat*. Peaks corresponding to 11-*cis*-RE (a) and all-*trans*-RE (b) are labeled in the left panel, focused on the RE elution timeframe; *syn*-11-*cis*-RO (c) and all-*trans*-RO (d) are labeled in the right panel, focused on the RO elution timeframe. **B.** Staggered traces from corresponding RPE/choroid extracts from the same animals analyzed in panel **A**. Peaks corresponding to all-*trans*-REs are labeled in the same manner as in panel **A**. **C.** Quantification and comparison of all-*trans*-RE #1, *syn*-11-*cis*-RO, and all-*trans*-RO levels in retinas and RPE/choroids from unbleached and post-3d-bleach AAV7m8-GFAP-*Lrat*-treated WT dark-adapted mice. Results from uncorrected Fisher's LSD tests conducted after two-way ANOVAs are provided above each plot (n ≥ 4; ns = not significant; * = $P \leq 0.05$; ** = $P \leq 0.01$; *** = $P \leq 0.001$; **** = $P \leq 0.0001$). Bars are plotted as mean ± SD. **D.** Staggered HPLC traces of neural retina retinoid extracts from DA *Lrat*$^{-/-}$ mice 3-weeks-post-transduction with GFAP-*Lrat,* focused on the RE (left) and RO (right) elution timeframes (n = 4). The predominant all-*trans*-RE species is labeled "a." **E.** Corresponding HPLC traces of RPE/choroid retinoid extracts from DA *Lrat*$^{-/-}$ animals, 3-weeks-post-transduction with GFAP-*Lrat*, focused on the RE and RO

elution timeframes (n = 4). **F.** Staggered HPLC traces of whole-eye retinoid extracts from untransduced *Rlbp1*$^{-/-}$ control (black traces) and *Rlbp1*$^{-/-}$ animals, 3-weeks post-transduction with GFAP-*Lrat* (blue traces), focused on the RE elution timeframe. Peaks corresponding to 11-*cis*- and all-*trans*-RE are labeled "a" and "b," respectively. **G.** Staggered HPLC traces of whole-eye retinoid extracts from untransduced *Rlbp1*$^{-/-}$ control (black traces) and *Rlbp1*$^{-/-}$ animals 3-weeks post-transduction with GFAP-*Lrat* (blue traces), focusing on the RO elution timeframe. Peaks corresponding to *syn*-11-*cis*-RO and all-*trans*-RO are labeled "c" and "d," respectively. For panels **A-B**, **D-E**, and **F-G**, dashed gray lines are provided to indicate the angle of offset.

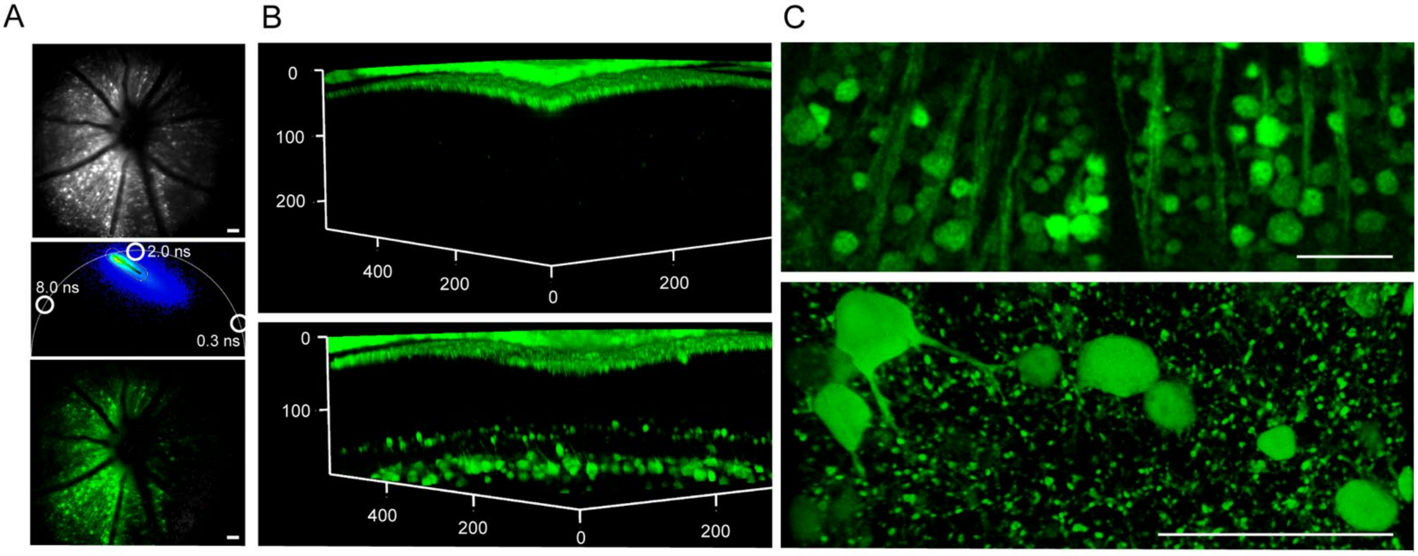

Supplemental Figure 12. Intravitreal transduction of retinal ganglion cells with AAV2-hSyn-eGFP. A. In vivo imaging through the pupil of the mouse eye. A fluorescence intensity-based image is shown in the upper panel. The middle panel shows a phasor plot. A green signal of varying intensity (arbitrary color) was assigned to the pixels in the bottom panel image displaying the fluorescence lifetime image (FLIM), according to the graded bar drawn through the phasor points (middle panel). The left end of the green bar is at 2.4 ns, corresponding to pixels displaying only eGFP fluorescence. Scale bars = 50 μm. **B.** Examples of 3-D-volume visualizations assembled from frames acquired every 1.5 μm along the retinal thickness in the intact mouse eye. In each 3-D visualization, the RPE is at z = 0 μm, and axes are labeled in μm. The top panel displays data from a mouse eye injected with 1/3 of the dose that was injected into the eye shown in the bottom panel. **C.** *Ex vivo* imaging of the intact mouse eye visualizing RGC bodies and axons, as shown in the 2-D image (upper panel) and 3-D projection (lower panel) showing cell bodies and dendritic details in the IPL. Scale bars = 50 μm.

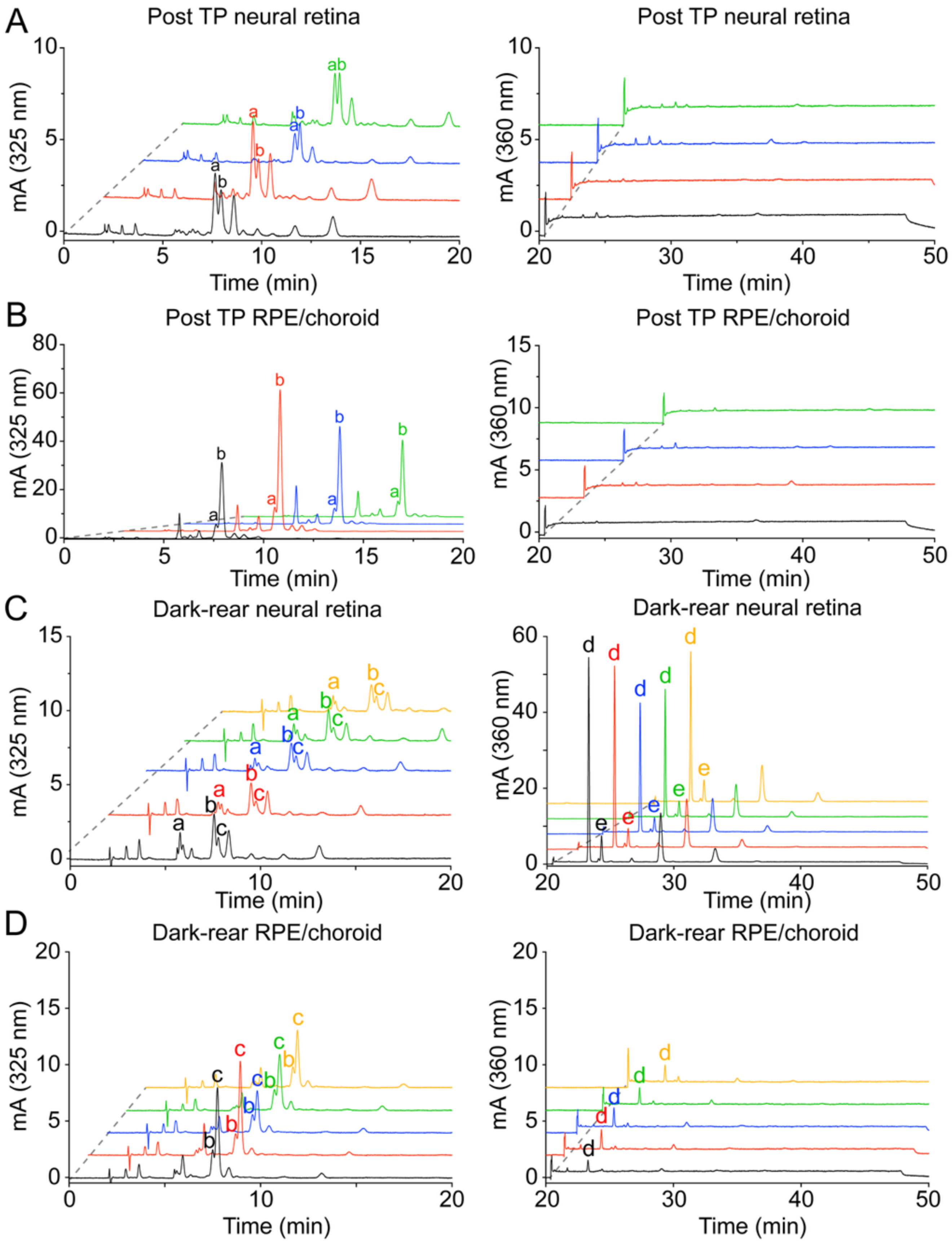

Supplemental Figure 13. Compiled retinoid analyses of light-adapted and dark-reared WT neural retina and RPE/choroid extracts, 6-weeks post-IV-transduction with AAV2-hSyn-*Lrat*. A. HPLC traces of retinoid

extracts from the neural retinas of B6(Cg)-*Tyr*$^{c\text{-}2J}$/J (WT) mice transduced with AAV2-hSyn-*Lrat* (6-weeks post-IV injection and TP imaging; light-adapted, n = 4). The peaks corresponding to all-*trans*-RE are labeled on each trace ("a" and "b"). **B.** HPLC traces of retinoid extracts from the RPE/choroid of B6(Cg)-*Tyr*$^{c\text{-}2J}$/J (WT) mice transduced with AAV2-hSyn-*Lrat* (6-weeks post-IV injection and TP imaging; light-adapted, n = 4). The all-*trans*-RE peaks are labeled on each trace ("a" and "b"). **C.** HPLC traces of retinoid extracts from the neural retinas of B6(Cg)-*Tyr*$^{c\text{-}2J}$/J (WT) mice transduced with AAV2-hSyn-*Lrat* (6-weeks post-IV injection and placement in darkroom; "dark-reared", n = 5). Peaks corresponding to 11-*cis*-RE ("a") and all-*trans*-RE ("b" and "c") are labeled on the left-hand panel, focused on the RE elution timeframe. The peaks corresponding to *syn*-11-*cis*-RO ("d") and all-*trans*-RO ("e") are labeled on the right-hand panel, focused on the RO elution timeframe. **D.** HPLC traces of retinoid extracts from the corresponding RPE/choroid of B6(Cg)-*Tyr*$^{c\text{-}2J}$/J (WT) mice transduced with AAV2-hSyn-*Lrat* (6-weeks post-IV injection and placement in darkroom; "dark-reared", n = 5). The peaks corresponding to all-*trans*-RE ("b" and "c") are labeled on the left-hand panel, focused on the RE elution timeframe. Peaks corresponding to *syn*-11-*cis*-RO ("d") are labeled on the right-hand panel, focused on the RO elution timeframe. For all panels, traces are staggered for ease of comparison; dashed gray lines indicate the angle of offset.

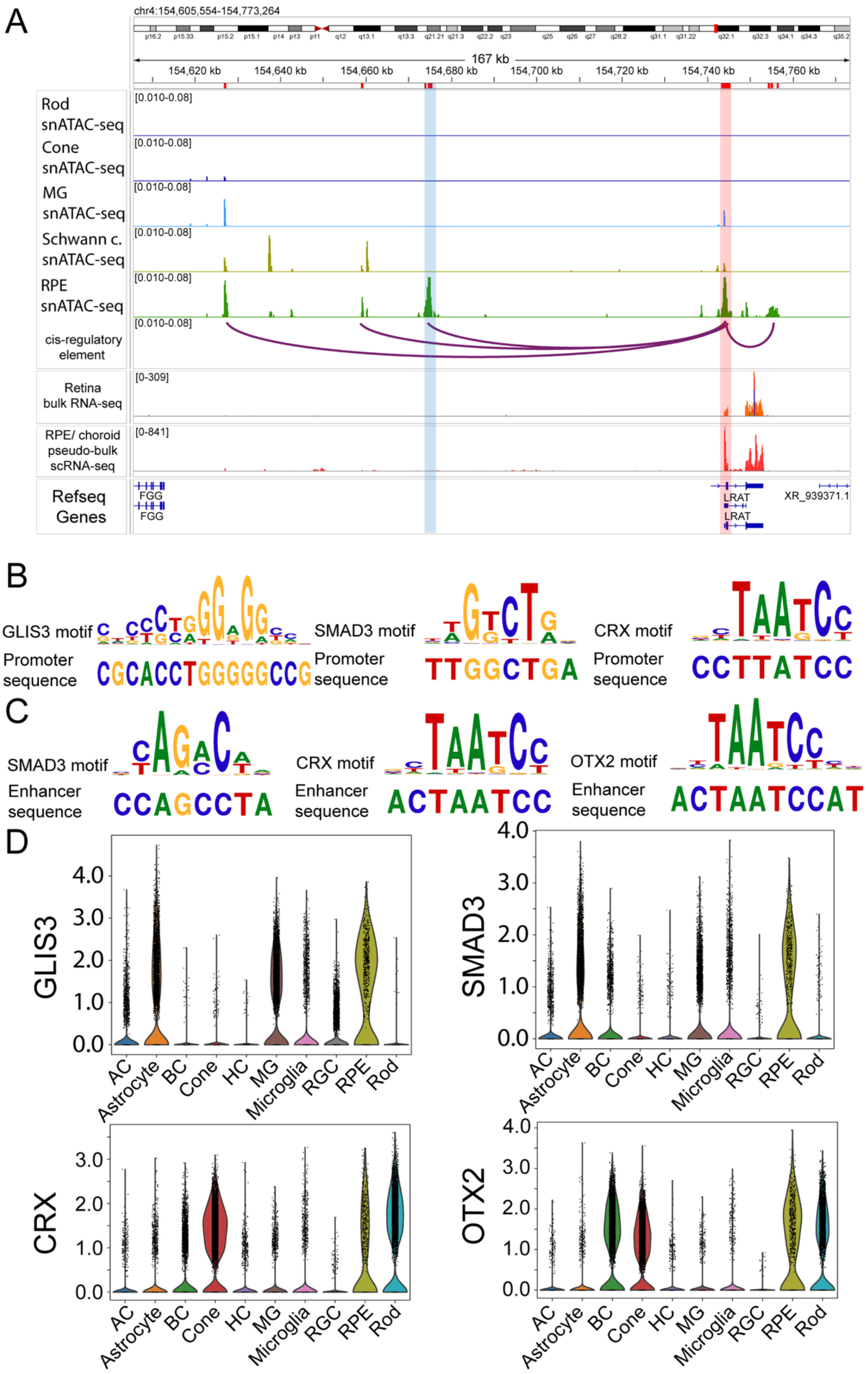
A
chr4:154,605,554-154,773,264
167 kb
154,620 kb
154,640 kb
154,660 kb
154,680 kb
154,700 kb
154,720 kb
154,740 kb
154,760 kb
Rod snATAC-seq
Cone snATAC-seq
MG snATAC-seq
Schwann c. snATAC-seq
RPE snATAC-seq
cis-regulatory element
[0.010-0.08]
Retina bulk RNA-seq
[0-309]
RPE/ choroid pseudo-bulk scRNA-seq
[0-841]
Refseq Genes
FGG
LRAT
XR_939371.1
B
GLIS3 motif
Promoter sequence
CGCACCTGGGGGCCG
SMAD3 motif
Promoter sequence
TTGGCTGA
CRX motif
Promoter sequence
CCTTATCC
C
SMAD3 motif
Enhancer sequence
CCAGCCTA
CRX motif
Enhancer sequence
ACTAATCC
OTX2 motif
Enhancer sequence
ACTAATCCAT
D
GLIS3
SMAD3
CRX
OTX2
AC
Astrocyte
BC
Cone
HC
MG
Microglia
RGC
RPE
Rod

Supplemental Figure 14. Potential cis-regulatory elements and transcription factors regulating LRAT expression. A. Genome tracks showing LRAT expression in RPE/choroid and retina, based on bulk RNA-seq data from adult human retina and pseudo-bulk scRNA-seq data from adult human RPE/choroid; and open chromatin regions in rod cells (Rod), cone cells (Cone), MG, Schwann cells, and RPE, based on snATAC-seq data. Putative cis-regulatory elements regulating LRAT expression are depicted. The promoter region is highlighted in red, and a strong enhancer is highlighted in blue. **B.** Putative promoter sequence motifs identified in the LRAT promoter region (highlighted in red in **A**). **C.** Putative enhancer sequence motifs identified in the strong enhancer region (highlighted in blue in panel **A**). **D.** Violin plots showing expression levels of transcription factors associated with motifs identified in the LRAT promoter and enhancer regions from panels **B** and **C** in the cell types of adult human retina.

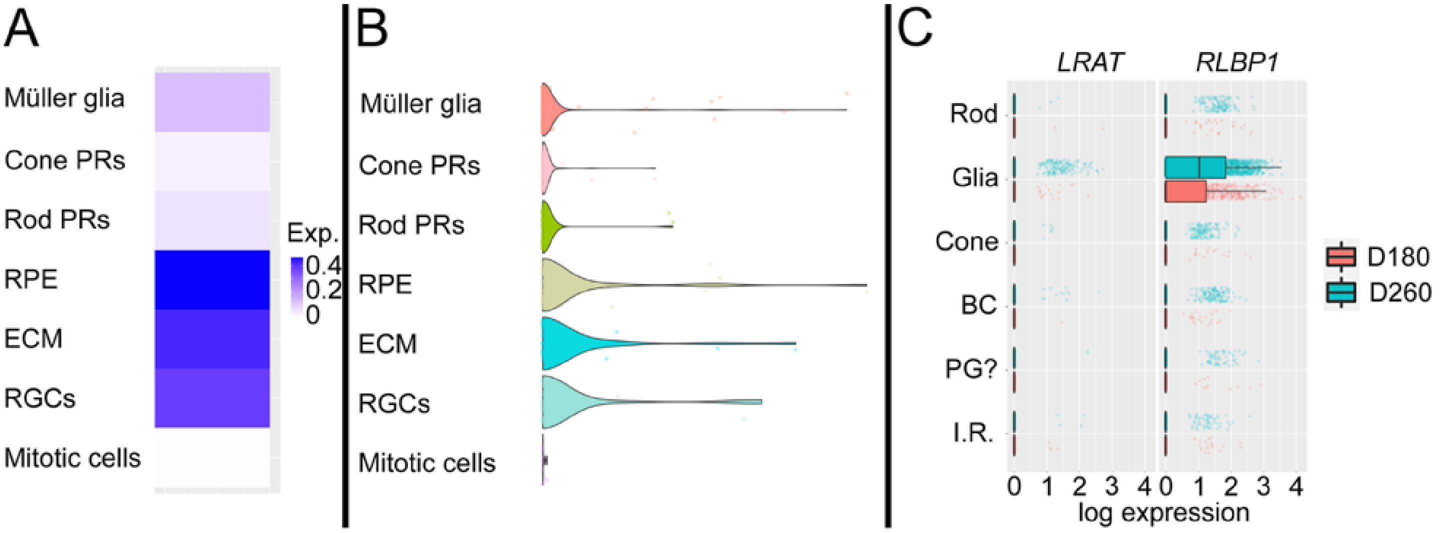

Supplemental Figure 15. Expression of LRAT in human retinal organoids. Heatmap (**A**) and violin plots (**B**), showing gene expression levels and cellular distribution of *LRAT* in human embryonic stem-cell-derived retinal organoids at D200 (32). **C.** Comparison of LRAT expression in D180 *vs* D260 retinal organoids derived from human induced pluripotent stem cells (data from Ref. (33); assayed using the Spectacle Website (12). RGCs = retinal ganglion cells; BC = bipolar cells; PG? = suspected retinal progenitors; I.R. = inner retina cells.

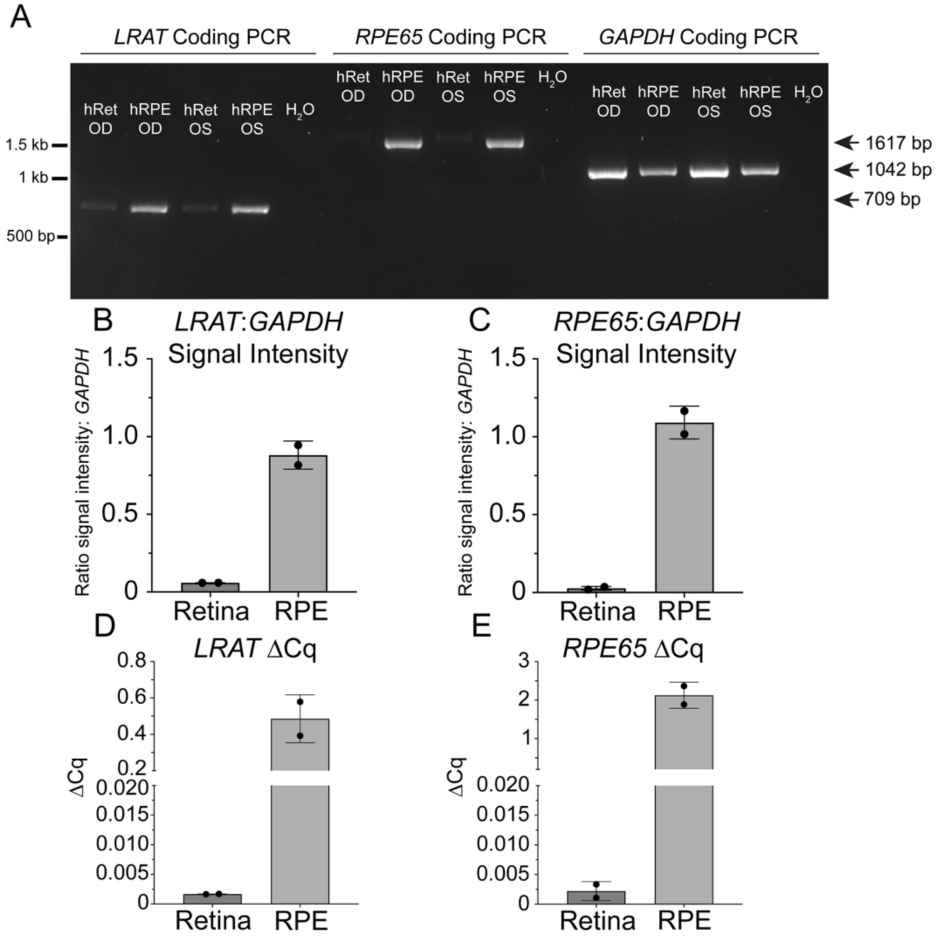

Supplemental Figure 16. PCRs and qPCRs of *LRAT*, *RPE65*, and *GAPDH* cDNAs synthesized from human neural retina and RPE/choroid mRNA extracts. A. SYBR Safe-stained gel image of PCR amplicons of the *LRAT*, *RPE65*, and *GAPDH* coding regions, using custom primers run on a 1.75% agarose gel. Expected amplicon sizes are listed below each reaction annotation. PCRs of the retina (hRet) and RPE/choroid (hRPE) from the right (OD) and left (OS) eyes of a single human donor are arranged in an alternating fashion for each reaction, along with a water-only negative control lane (H_2O). A molecular mass reference is provided on the left. **B.** Quantification of the signal intensity of the *LRAT* PCR amplicon normalized to the *GAPDH* PCR-amplicon signal intensity for human neural retina and RPE/choroid samples (n = 2; bars represent mean ± SD). **C.** Quantification of *RPE65* PCR-amplicon signal intensity normalized to *GAPDH* PCR-amplicon signal intensity for human neural retina and RPE/choroid samples (n = 2; bars indicate mean ± SD). **D.** *LRAT* ΔCq values normalized to the *GAPDH* signal for retina and RPE/choroid cDNA qPCRs (n = 2, individual values are averaged from two technical replicates; bars represent mean ± SD). **E.** *RPE65* ΔCq values normalized to the *GAPDH* signal for retina and RPE/choroid cDNA qPCRs (n = 2, individual values are averaged from two technical replicates; bars represent mean ± SD).

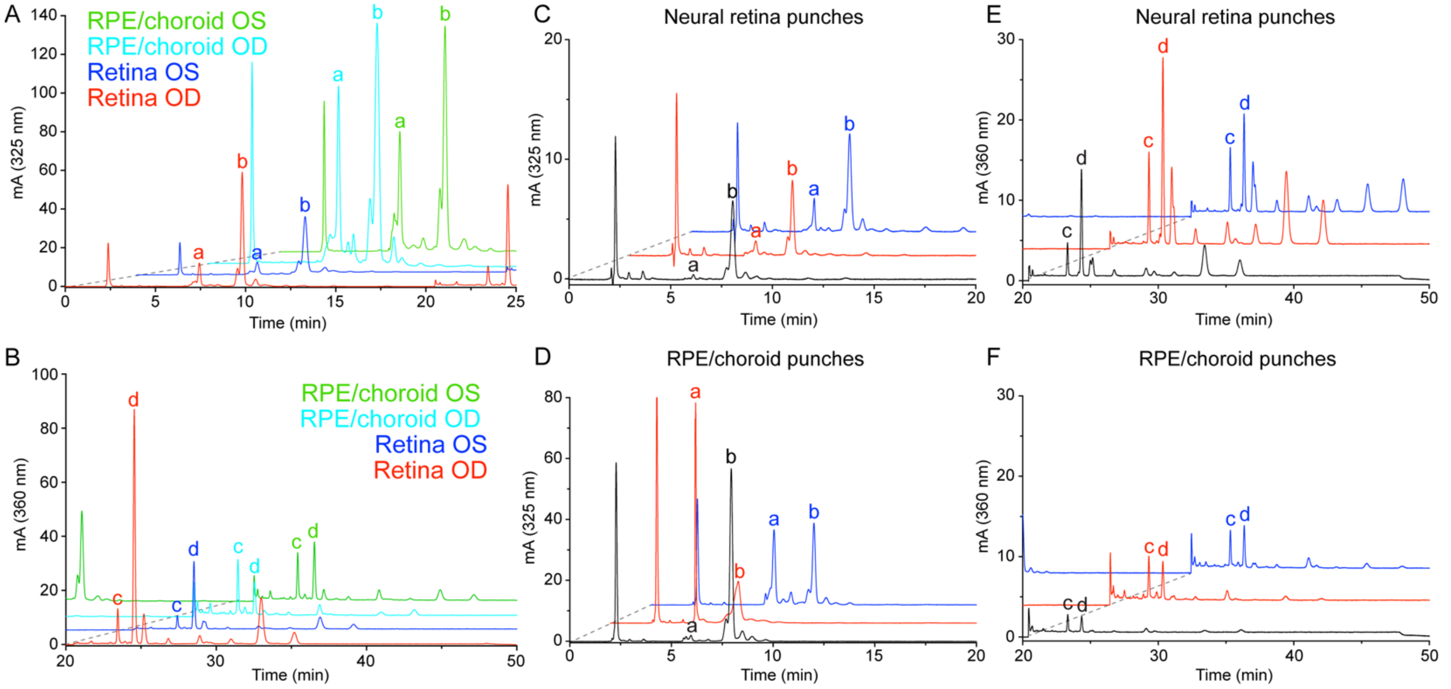

Supplemental Figure 17. Compiled retinoid analyses from human retina and RPE/choroid material. A. Compiled analyses of retinoid extracts from whole light-adapted human neural retina and corresponding RPE/choroid, with a focus on the RE elution timeframe. Peaks corresponding to 11-*cis*- and all-*trans*-RE are labeled "a" and "b," respectively (n = 2 per tissue type). **B.** Compiled analyses of retinoid extracts from light-adapted whole human retina and corresponding RPE/choroid tissue, with a focus on the RO elution timeframe. Peaks corresponding to 11-*cis*- and all-*trans*-RO are labeled "c" and "d," respectively (n = 2 per tissue type). **C.** Compiled analyses of retinoid extracts from consolidated, light-adapted human perimacular neural retina punches, with a focus on the RE elution timeframe. Peaks corresponding to 11-*cis*- and all-*trans*-RE are labeled "a" and "b," respectively (n = 3). **D.** Corresponding analyses of retinoid extracts from consolidated, light-adapted human perimacular RPE/choroid punches, with a focus on the RE elution timeframe. Peaks corresponding to 11-*cis*- and all-*trans*-RE are labeled "a" and "b," respectively (n = 3). **E.** Compiled analyses of retinoid extracts from consolidated light-adapted human perimacular neural retina punches, with a focus on the RO elution timeframe. Peaks corresponding to 11-*cis*- and all-*trans*-RO are labeled "c" and "d", respectively (n = 3). **F.** Corresponding analyses of retinoid extracts from consolidated, light-adapted human perimacular RPE/choroid punches, with a focus on the RO elution timeframe. Peaks corresponding to 11-*cis*- and all-*trans*-RO are labeled "c" and "d," respectively (n = 3). Dashed gray lines indicate the angle of offset in each panel.

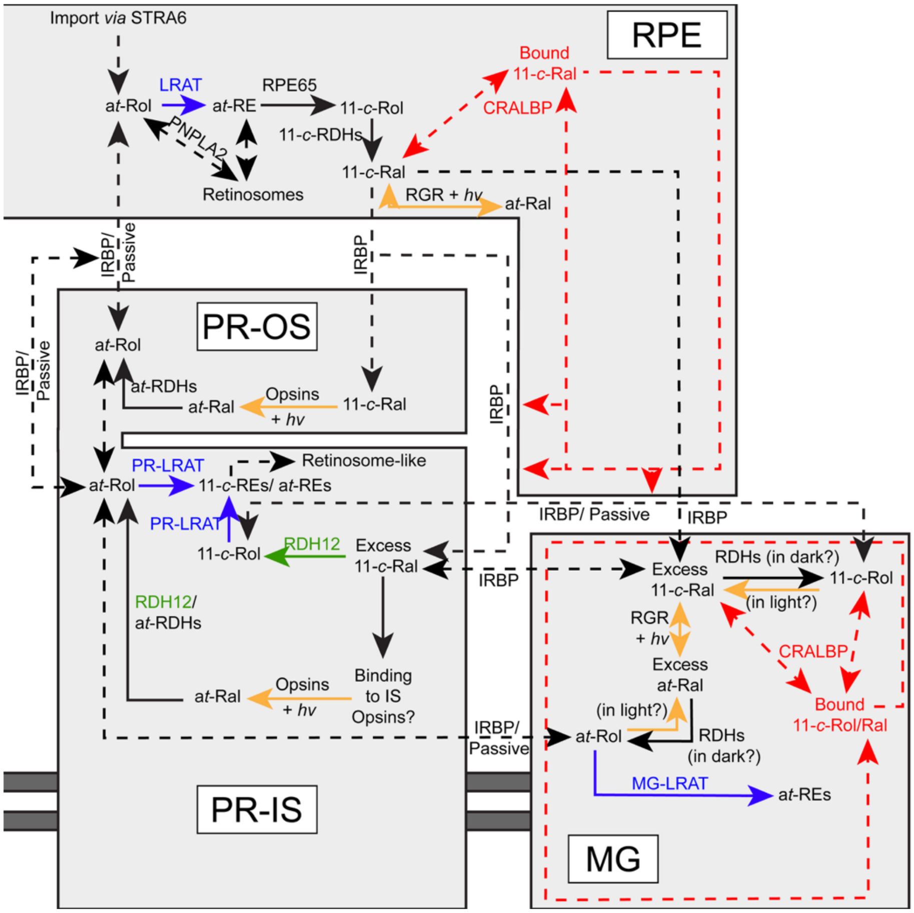

Supplemental Figure 18. A map of murine RPE-PR-MG retinoid flux. at-Rol enters the retina from circulation via STRA6 in basal RPE membranes (34). The at-Rol then enters the visual cycle and is converted to at-REs by LRAT in the RPE (1, 35). These REs self-assemble into retinosomes (36) and are mobilized by PNPLA2 to re-entry into the visual cycle (37). RPE65 then generates 11-cis-retinol (11-c-Rol) (38-40). 11-c-RDH (e.g., RDH5) oxidizes the 11-c-Rol to 11-cis-retinal (11-c-Ral), preventing scavenging of free 11-c-Rol by CRALBP (3, 41). Excess 11-c-Ral, however, is likely bound and shuttled intracellularly within the RPE by CRALBP, serving as a reservoir for 11-c-Ral and accelerating 11-c-Ral accessibility to the PRs. RGR-opsin in the RPE interacts with CRALBP and participates in photic regeneration of visual chromophore from at-Ral (42). 11-c-Ral is then freed from RPE membranes and shuttled to PRs through the interphotoreceptor space, bound to IRBP (43). There is also evidence to support the shuttling of excess IRBP-11-c-Ral complex to MG endfeet (44). Once 11-c-Ral reaches PR outer segments (PR-OS), it forms a covalent bond within the binding pockets of opsins (45). Following photoisomerization of 11-c-Ral adducted to opsins, it is hydrolytically released, and the freed all-trans-retinal (at-Ral) is reduced to all-trans-retinol (at-Rol) (46), where it binds to IRBP or passively diffuses back to the RPE for re-entry into the visual cycle

(35). Based on our observations, 11-c-Ral could make its way from the RPE, PR-OS, or MG to the PR-IS, where it either binds to newly-expressed opsins in the PR-IS, or is rapidly reduced to 11-c-Rol by RDH12 (47, 48). Reduction of any excess at-Ral to at-Rol is likely carried out by multiple PR-localized RDHs (49). PR-IS-localized 11-c-Rol and at-Rol can then passively diffuse or be shuttled by IRBP to other cellular compartments, such as the PR-OS, RPE, or MG endfeet. RGR-opsin in MG is implicated in a cone-specific visual cycle, but it remains unclear whether the redox state in MG could support selective oxidation of 11-c-Rol to 11-c-Ral while selectively reducing at-Ral to at-Rol. Our observations support the reduction of free Ral to Rol in MG and PR-IS under DA conditions, rather than oxidation of Rol to Ral for visual chromophore regeneration.

SUPPLEMENTARY VIDEO CAPTIONS

Video 1 shows a series of *en face* images obtained every 2 µm along the thickness of the retina extending from the RPE to outer plexiform layer (OPL) in the intact eye of a 6-week-old PR-*Lrat*$^{+/+}$*Lrat*$^{+/-}$ mouse. First frame is at the RPE layer.

Video 2 shows a series of *en face* images obtained every 2 µm along the thickness of the retina extending from the RPE to the OPL in the intact eye of a 6-week-old PR-*Lrat*$^{+/-}$*Lrat*$^{+/-}$ mouse. First frame is at the RPE layer.

Video 3 shows a series of *en face* images obtained every 2 µm along the thickness of the retina extending from the RPE to the OPL in the intact eye of a 6-week-old PR-*Lrat*$^{-/-}$*Lrat*$^{+/-}$ mouse. First frame is at the RPE layer.

Video 4 shows a series of *en face* images obtained every 2 µm along the thickness of the retina extending from the RPE to the OPL in the intact eye of a 6-week-old PR-*Lrat*$^{+/+}$*Lrat*$^{-/-}$ mouse. First frame is at the RPE layer.

REFERENCES

1. Batten ML, Imanishi Y, Maeda T, et al. Lecithin-retinol acyltransferase is essential for accumulation of all-trans-retinyl esters in the eye and in the liver. *J Biol Chem.* 2004;279(11):10422-32.

2. Calvert PD, Krasnoperova NV, Lyubarsky AL, et al. Phototransduction in transgenic mice after targeted deletion of the rod transducin alpha -subunit. *Proc Natl Acad Sci U S A.* 2000;97(25):13913-8.

3. Saari JC, Nawrot M, Kennedy BN, et al. Visual cycle impairment in cellular retinaldehyde binding protein (CRALBP) knockout mice results in delayed dark adaptation. *Neuron.* 2001;29(3):739-48.

4. Maeda A, Maeda T, Imanishi Y, et al. Role of photoreceptor-specific retinol dehydrogenase in the retinoid cycle in vivo. *J Biol Chem.* 2005;280(19):18822-32.

5. Palczewska G, Wojtkowski M, and Palczewski K. From mouse to human: Accessing the biochemistry of vision in vivo by two-photon excitation. *Prog Retin Eye Res.* 2023;93:101170.

6. Palczewska G, Boguslawski J, Stremplewski P, et al. Noninvasive two-photon optical biopsy of retinal fluorophores. *Proc Natl Acad Sci U S A.* 2020;117(36):22532-43.

7. Stremplewski P, Komar K, Palczewski K, et al. Periscope for noninvasive two-photon imaging of murine retina in vivo. *Biomed Opt Express.* 2015;6(9):3352-61.

8. Samimi K, Pasachhe O, Guzman EC, et al. Autofluorescence lifetime flow cytometry with time-correlated single photon counting. *Cytometry A.* 2024;105(8):607-20.

9. Schindelin J, Arganda-Carreras I, Frise E, et al. Fiji: an open-source platform for biological-image analysis. *Nat Methods.* 2012;9(7):676-82.

10. Adamus G, Zam ZS, Arendt A, et al. Anti-rhodopsin monoclonal antibodies of defined specificity: characterization and application. *Vision Res.* 1991;31(1):17-31.

11. Golczak M, Kiser PD, Lodowski DT, et al. Importance of membrane structural integrity for RPE65 retinoid isomerization activity. *J Biol Chem.* 2010;285(13):9667-82.

12. Voigt AP, Whitmore SS, Lessing ND, et al. Spectacle: An interactive resource for ocular single-cell RNA sequencing data analysis. *Exp Eye Res.* 2020;200:108204.

13. Voigt AP, Whitmore SS, Mulfaul K, et al. Bulk and single-cell gene expression analyses reveal aging human choriocapillaris has pro-inflammatory phenotype. *Microvasc Res.* 2020;131:104031.

14. Gautam P, Hamashima K, Chen Y, et al. Multi-species single-cell transcriptomic analysis of ocular compartment regulons. *Nat Commun.* 2021;12(1):5675.

15. Voigt AP, Mullin NK, Mulfaul K, et al. Choroidal endothelial and macrophage gene expression in atrophic and neovascular macular degeneration. *Hum Mol Genet.* 2022;31(14):2406-23.

16. Collin J, Hasoon MSR, Zerti D, et al. Single-cell RNA sequencing reveals transcriptional changes of human choroidal and retinal pigment epithelium cells during fetal development, in healthy adult and intermediate age-related macular degeneration. *Hum Mol Genet.* 2023;32(10):1698-710.

17. Mullin NK, Voigt AP, Flamme-Wiese MJ, et al. Multimodal single-cell analysis of nonrandom heteroplasmy distribution in human retinal mitochondrial disease. *JCI Insight.* 2023;8(14).

18. Li R, Liu J, Yi P, et al. Integrative Single-Cell Transcriptomics and Epigenomics Mapping of the Fetal Retina Developmental Dynamics. *Adv Sci (Weinh).* 2023;10(16):e2206623.

19. Liang Q, Cheng X, Wang J, et al. A multi-omics atlas of the human retina at single-cell resolution. *Cell Genom.* 2023;3(6):100298.

20. Wang J, Cheng X, Liang Q, et al. Single-cell multiomics of the human retina reveals hierarchical transcription factor collaboration in mediating cell type-specific effects of genetic variants on gene regulation. *Genome Biol.* 2023;24(1):269.

21. Hahn J, Monavarfeshani A, Qiao M, et al. Evolution of neuronal cell classes and types in the vertebrate retina. *Nature.* 2023;624(7991):415-24.

22. Li J, Wang J, Ibarra IL, et al. Integrated multi-omics single cell atlas of the human retina. *Res Sq.* 2023.

23. Lopez R, Regier J, Cole MB, et al. Deep generative modeling for single-cell transcriptomics. *Nat Methods.* 2018;15(12):1053-8.

24. Li M, Jia C, Kazmierkiewicz KL, et al. Comprehensive analysis of gene expression in human retina and supporting tissues. *Hum Mol Genet.* 2014;23(15):4001-14.

25. Li J, Choi J, Cheng X, et al. Comprehensive single-cell atlas of the mouse retina. *iScience.* 2024;27(6):109916.

26. Oguchi A, Suzuki A, Komatsu S, et al. An atlas of transcribed enhancers across helper T cell diversity for decoding human diseases. *Science.* 2024;385(6704):eadd8394.

27. Heinz S, Benner C, Spann N, et al. Simple combinations of lineage-determining transcription factors prime cis-regulatory elements required for macrophage and B cell identities. *Mol Cell.* 2010;38(4):576-89.

28. Leinonen H, Choi EH, Gardella A, et al. A Mixture of U.S. Food and Drug Administration-Approved Monoaminergic Drugs Protects the Retina From Light Damage in Diverse Models of Night Blindness. *Invest Ophthalmol Vis Sci.* 2019;60(5):1442-53.

29. Leinonen H, Zhang J, Occelli LM, et al. A combination treatment based on drug repurposing demonstrates mutation-agnostic efficacy in pre-clinical retinopathy models. *Nat Commun.* 2024;15(1):5943.

30. Ye J, Coulouris G, Zaretskaya I, et al. Primer-BLAST: a tool to design target-specific primers for polymerase chain reaction. *BMC Bioinformatics.* 2012;13:134.

31. Samimi K, Pattnaik BR, Capowski EE, et al. In situ autofluorescence lifetime assay of a photoreceptor stimulus response in mouse retina and human retinal organoids. *Biomed Opt Express.* 2022;13(6):3476-92.

32. Collin J, Queen R, Zerti D, et al. Deconstructing Retinal Organoids: Single Cell RNA-Seq Reveals the Cellular Components of Human Pluripotent Stem Cell-Derived Retina. *Stem Cells.* 2019;37(5):593-8.

33. Mullin NK, Bohrer LR, Voigt AP, et al. NR2E3 loss disrupts photoreceptor cell maturation and fate in human organoid models of retinal development. *J Clin Invest.* 2024;134(11).
