# Supplementary material for: Retinol tracing within murine neural retina reveals cell type–specific retinol transport and distribution
Source: J Clin Invest. 2025 Nov 18;136(3):e198648. doi: 10.1172/JCI198648 (PMC12867154; doi:10.1172/JCI198648)
Supplement: Unedited blot and gel images [file jci-136-198648-s166.pdf]

Uncropped/ Unedited Gel and Blot Images

Co-opting a visual cycle protein to track retinoid transport within the neural retina

Zachary J. Engfer, Grazyna Palczewska, Samuel W. Du, Jianye Zhang, Zhiqian Dong, Carolline Rodrigues Menezes, Jun Wang, Jianming Shao, Budd A Tucker, Robert F. Mullins, Rui Chen, Philip D. Kiser, Krzysztof Palczewski

Figure 2 Panel C

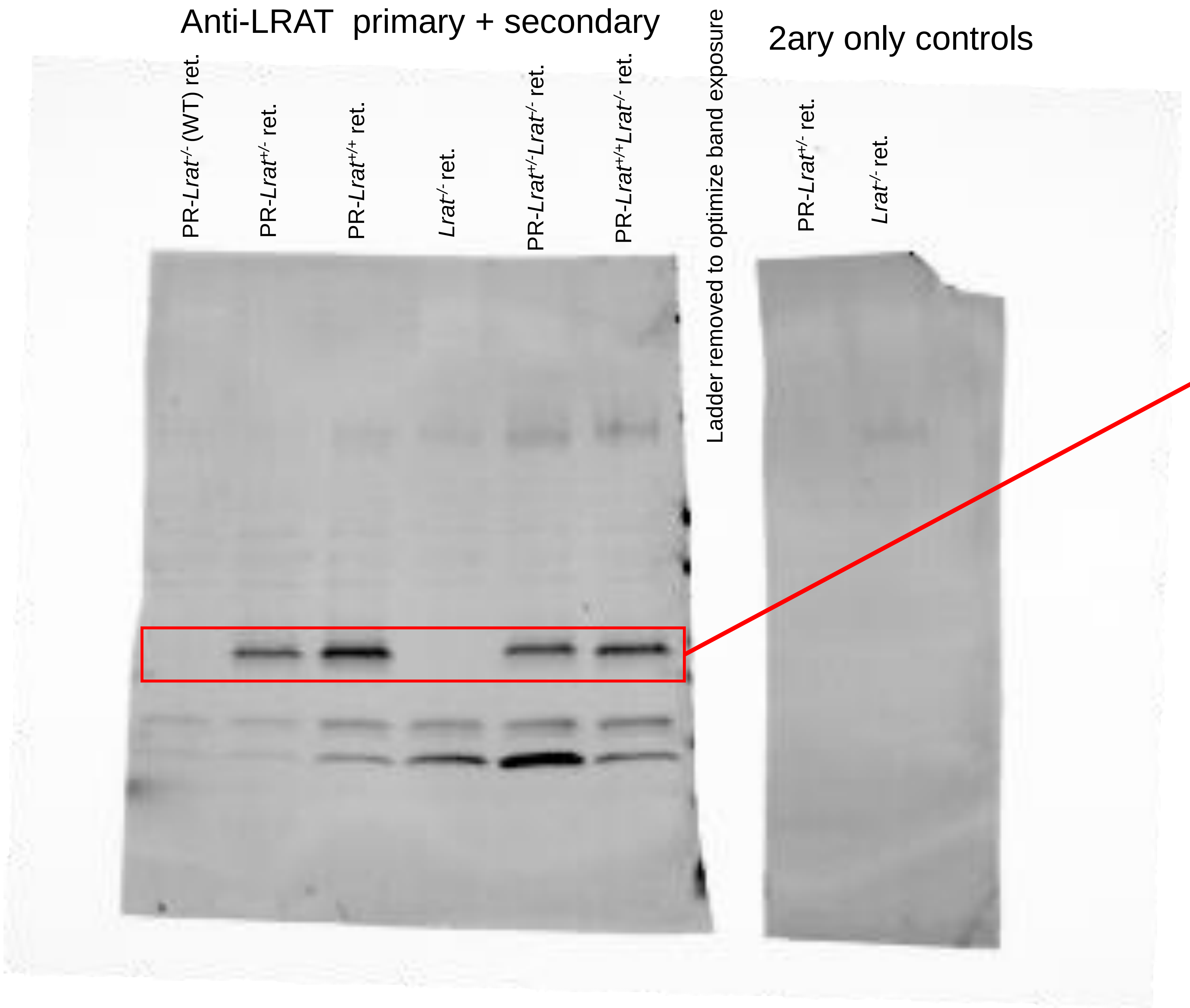

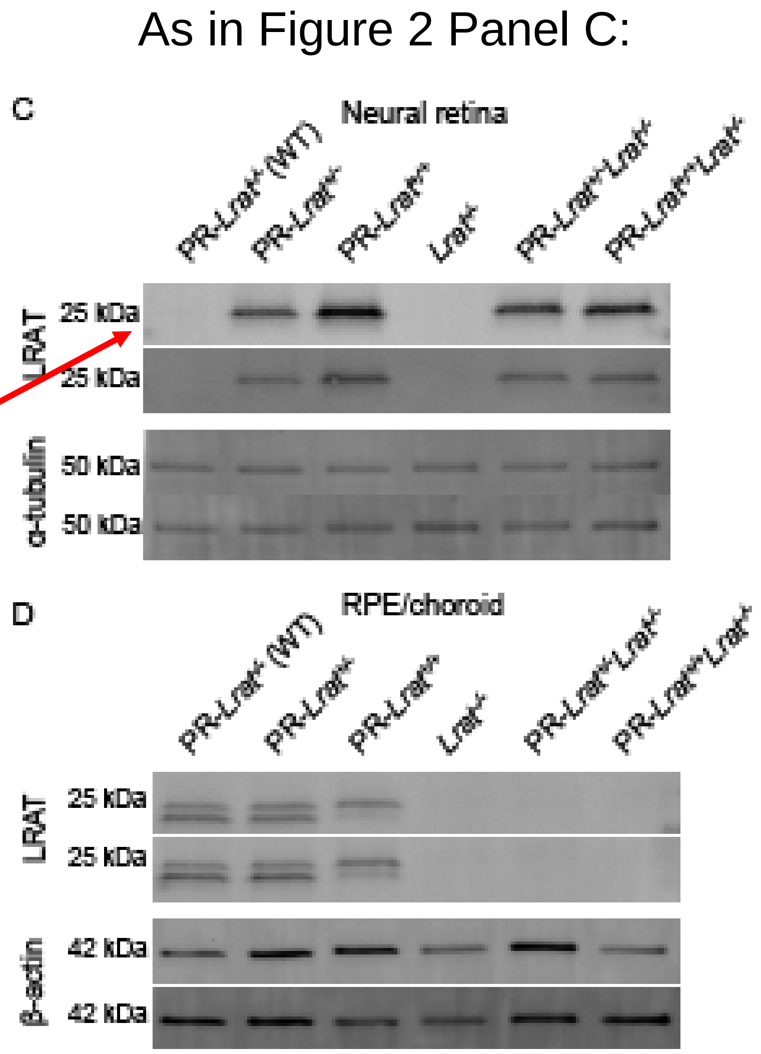

*See same blot w/ ladder on n[illegible]

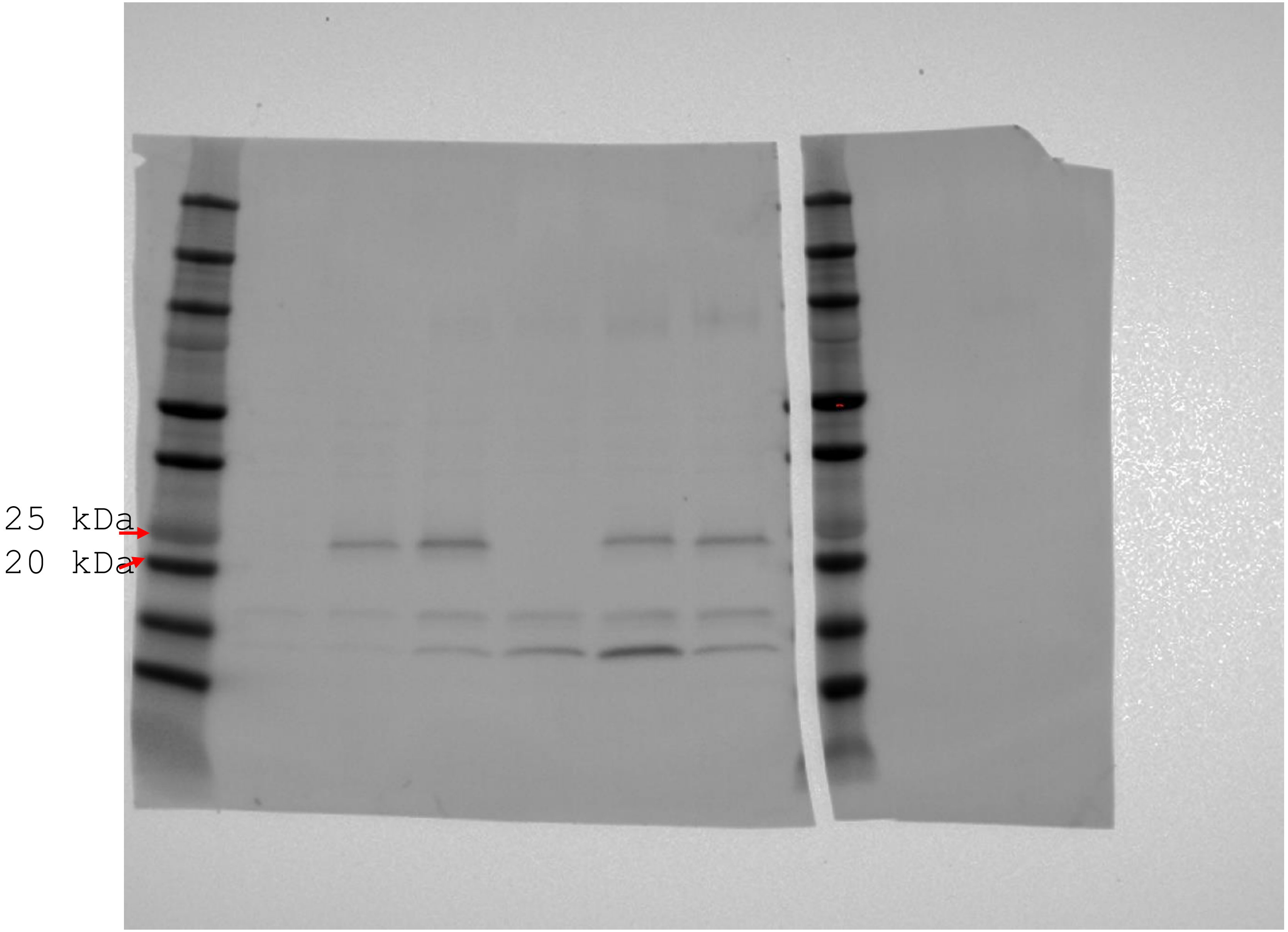

Same blot as previous slide but with molecular weight ladders (Precision Plus Protein Dual Color Standard (BioRad Cat #: 1610374)

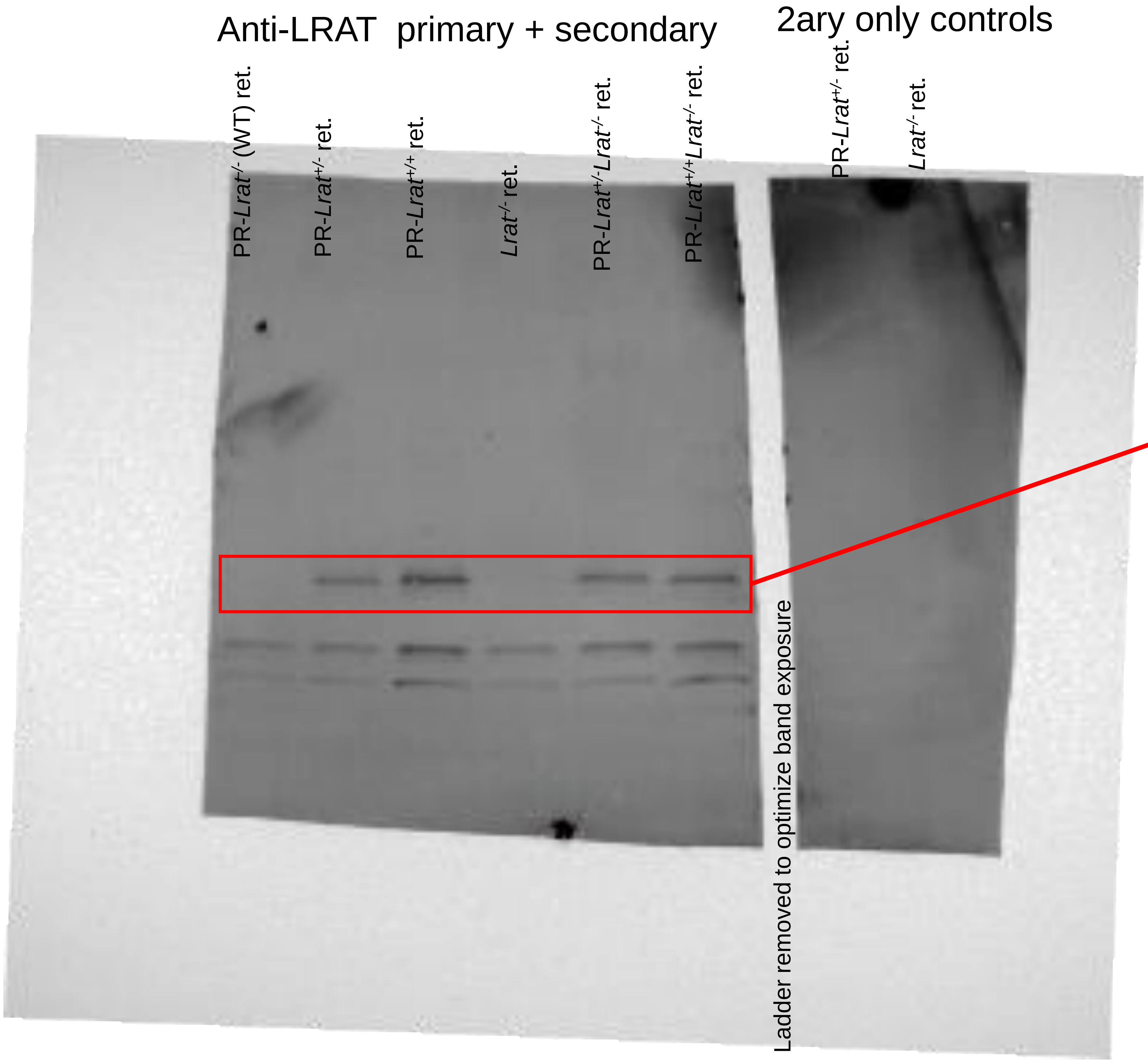

As in Figure 2 Panel C:

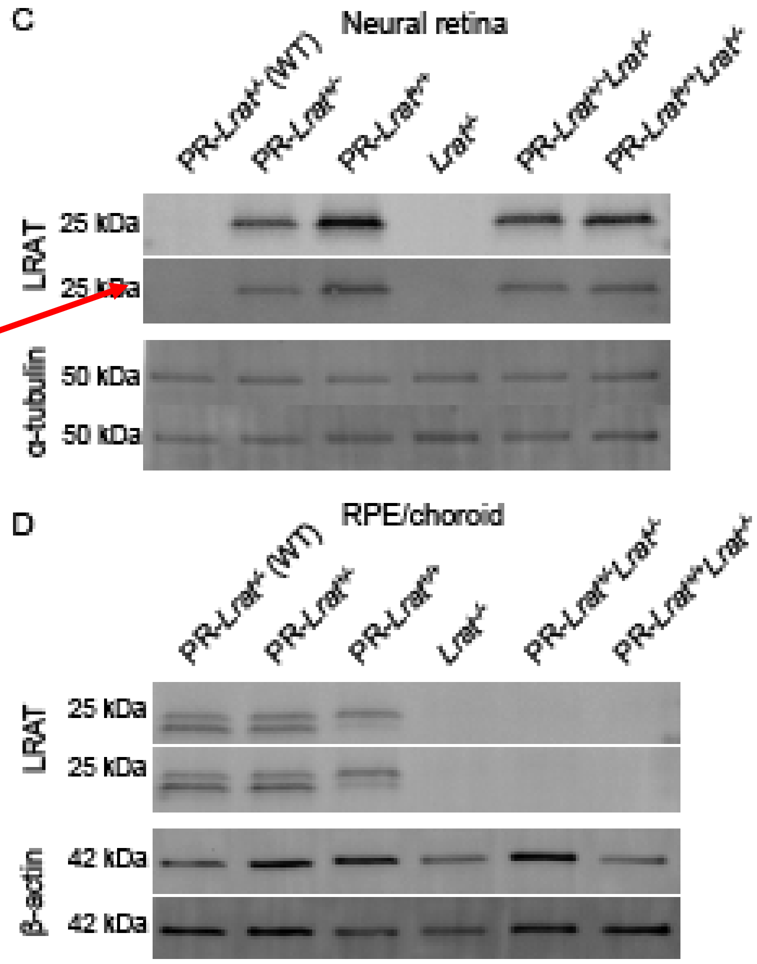

*See same blot w/ ladder on n

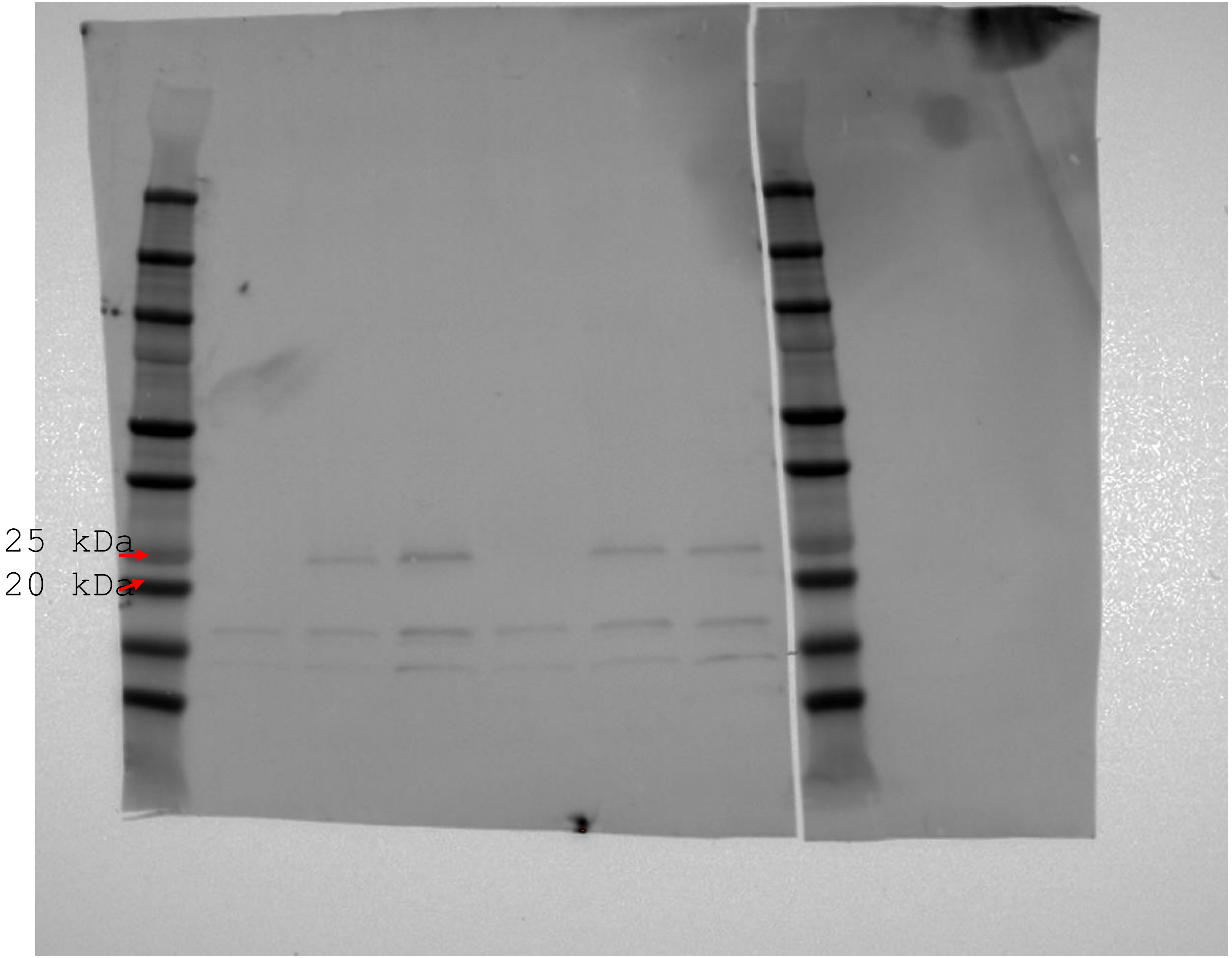

Same blot as previous slide but with molecular weight ladders (Precision Plus Protein Dual Color Standard (BioRad Cat #: 1610374)

Anti-alpha tubulin primary + secondary 2ary only controls

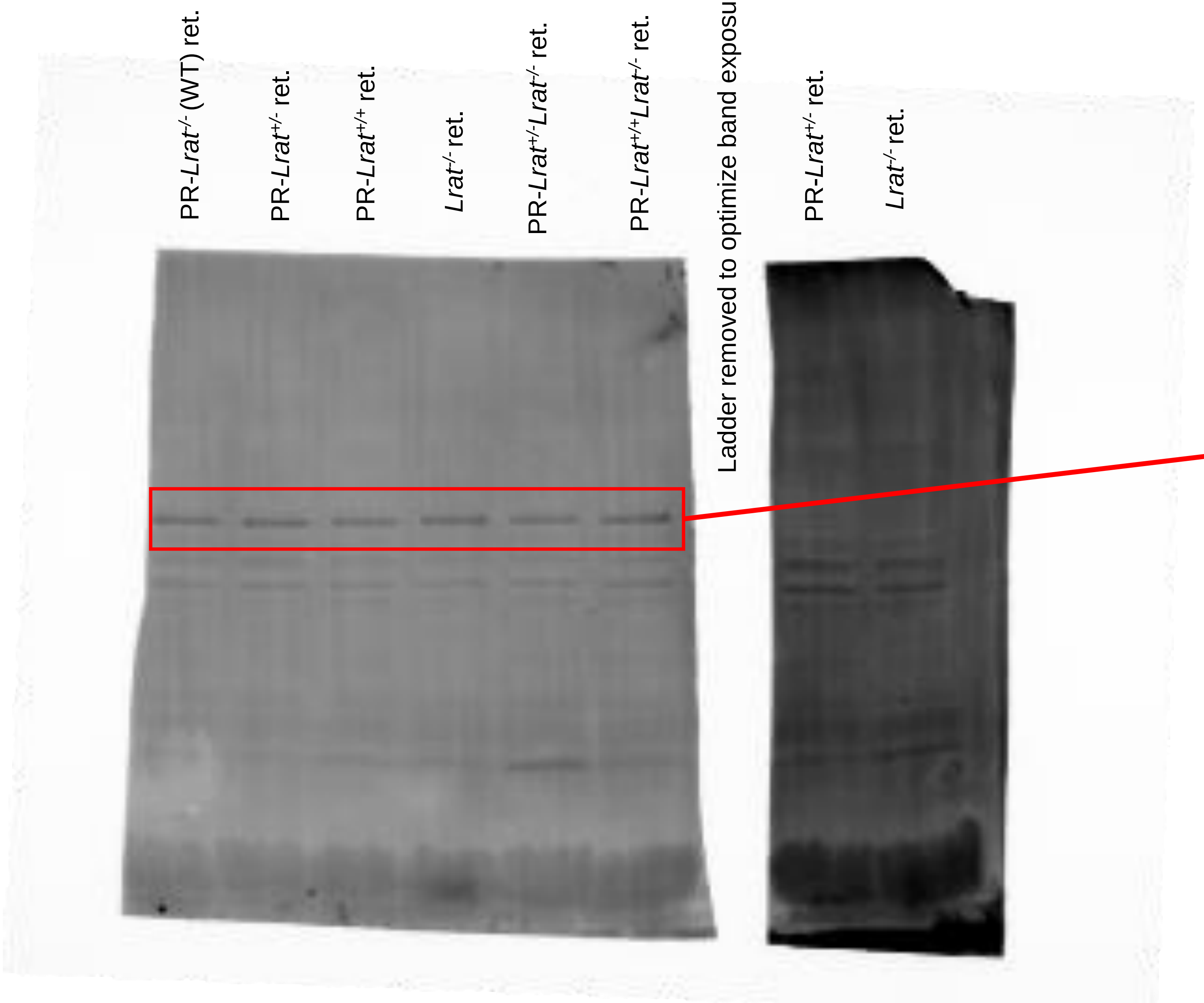

As in Figure 2 Panel C:

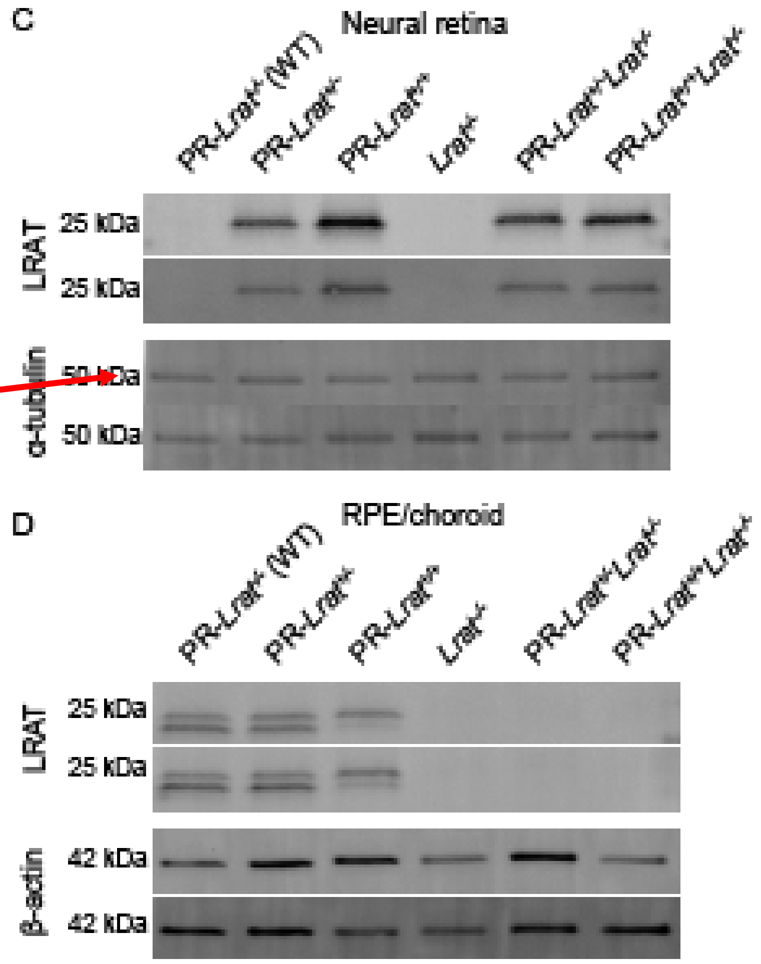

*Different antibody host species, secondary, channel used for alpha tubulin ; same blot as featured in slides 3 + 4 staining for anti LRAT.

*See same blot w/ ladder on n[illegible]

50 kDa

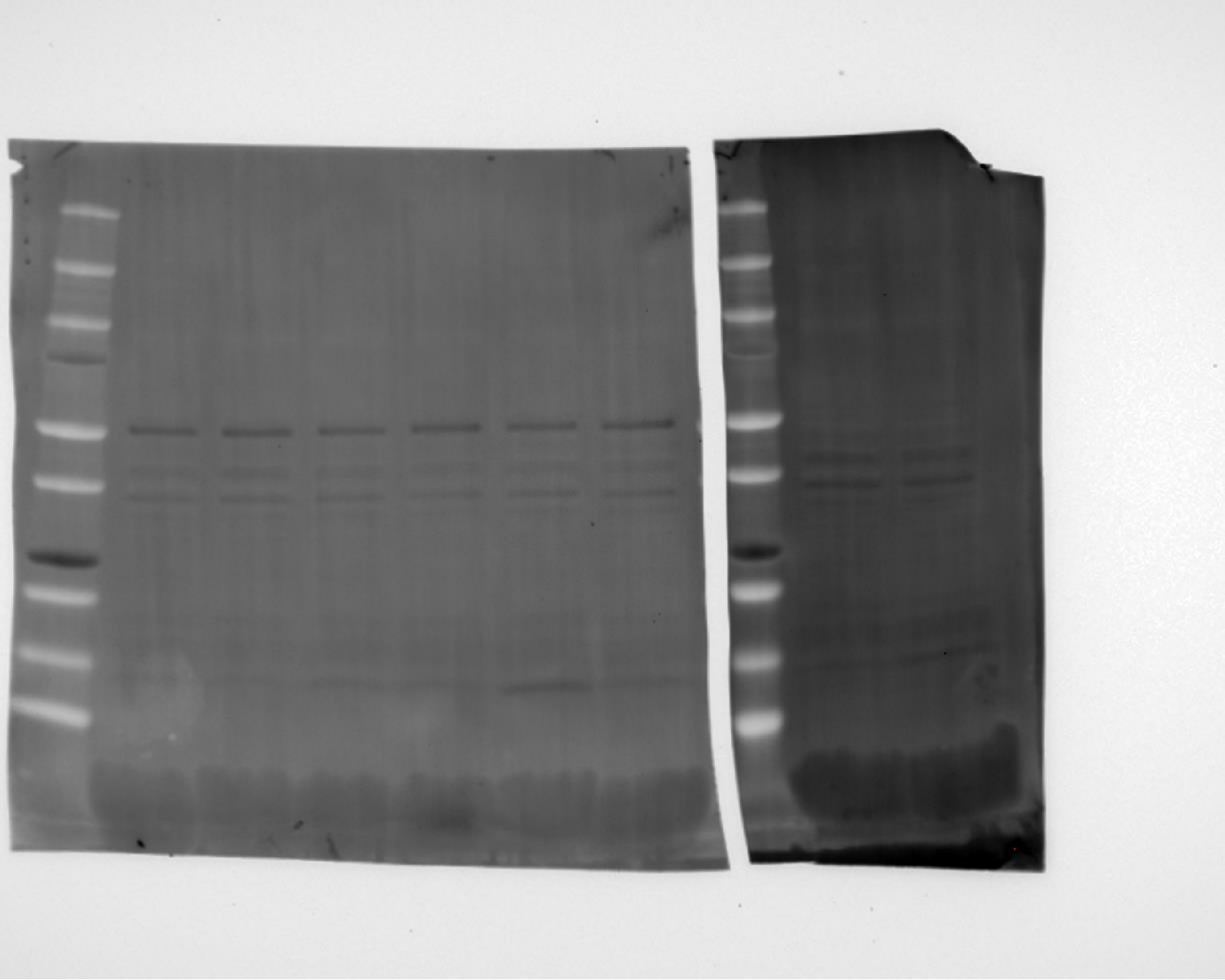

Same blot as previous slide but with molecular weight ladders (Precision Plus Protein Dual Color Standard (BioRad Cat #: 1610374)

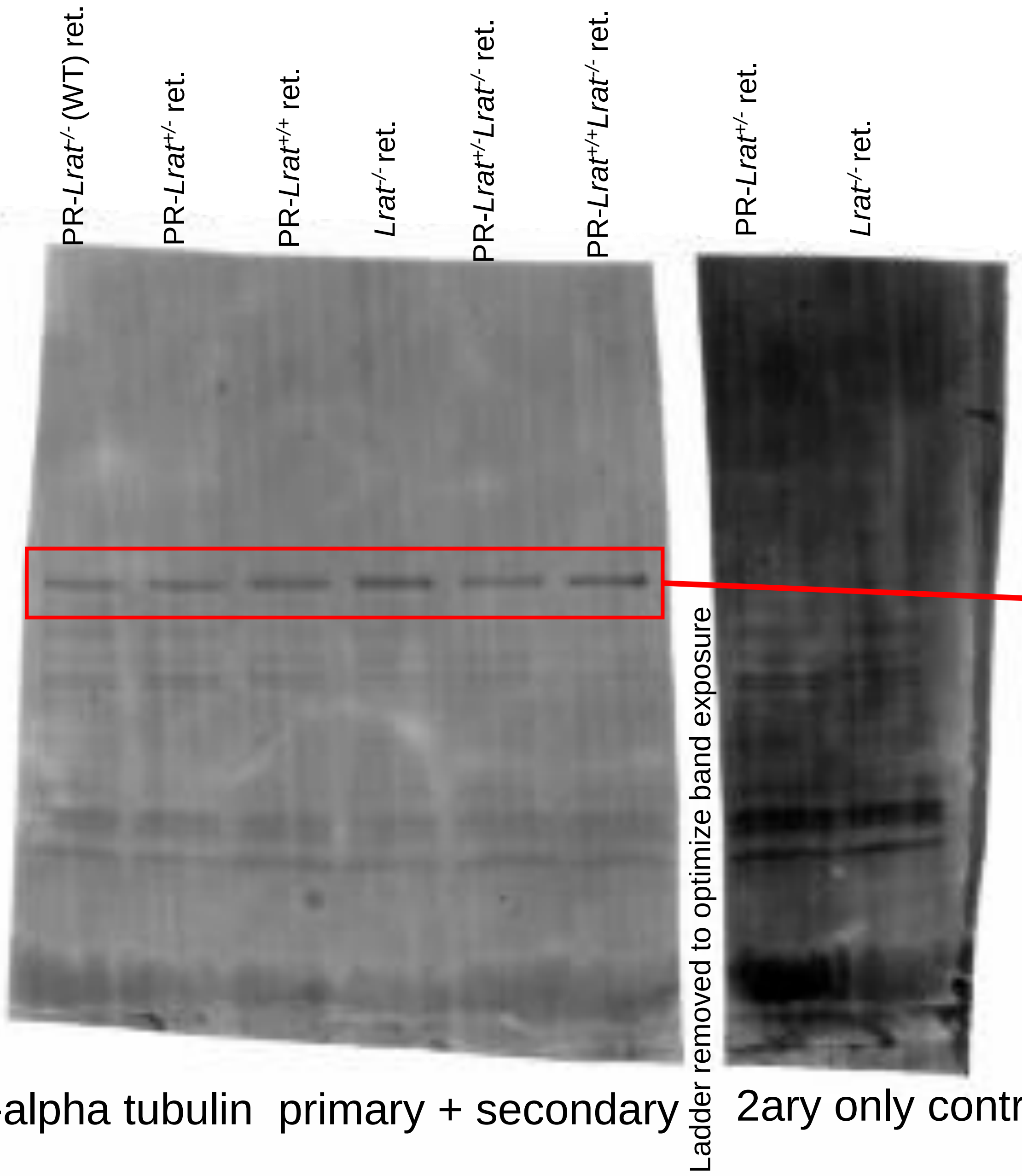

Anti-alpha tubulin primary + secondary

2ary only controls

As in Figure 2 Panel C:

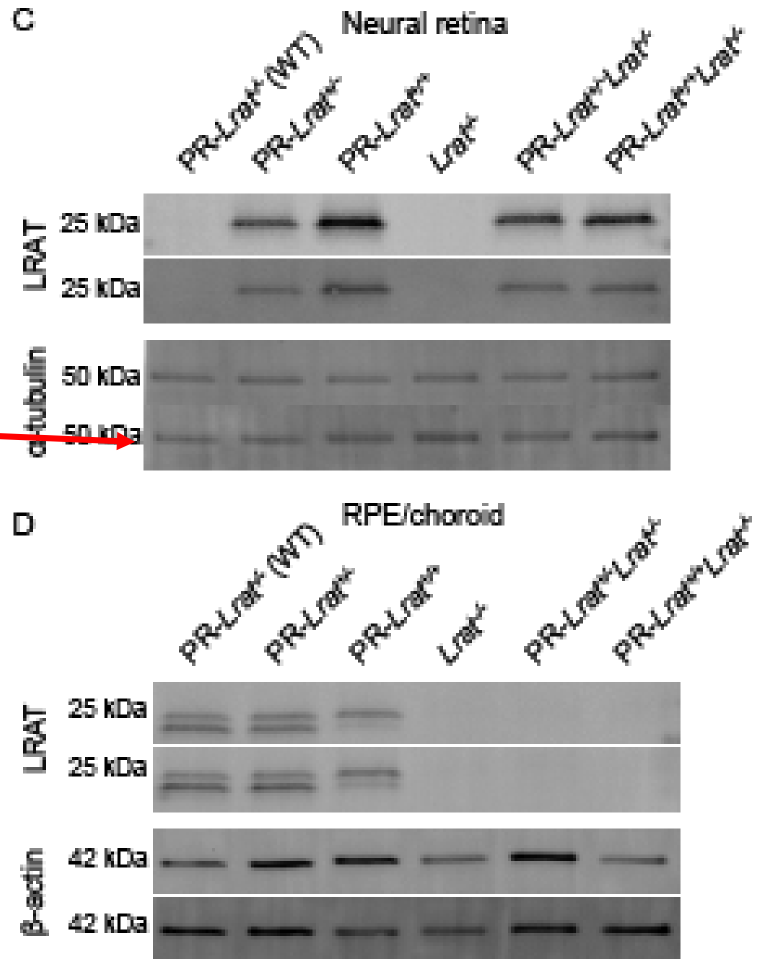

*Different antibody host species, secondary, channel used for alpha tubulin; same blot as featured in slides 5 + 6 staining for anti LRAT.

*See same blot w/ ladder on ne

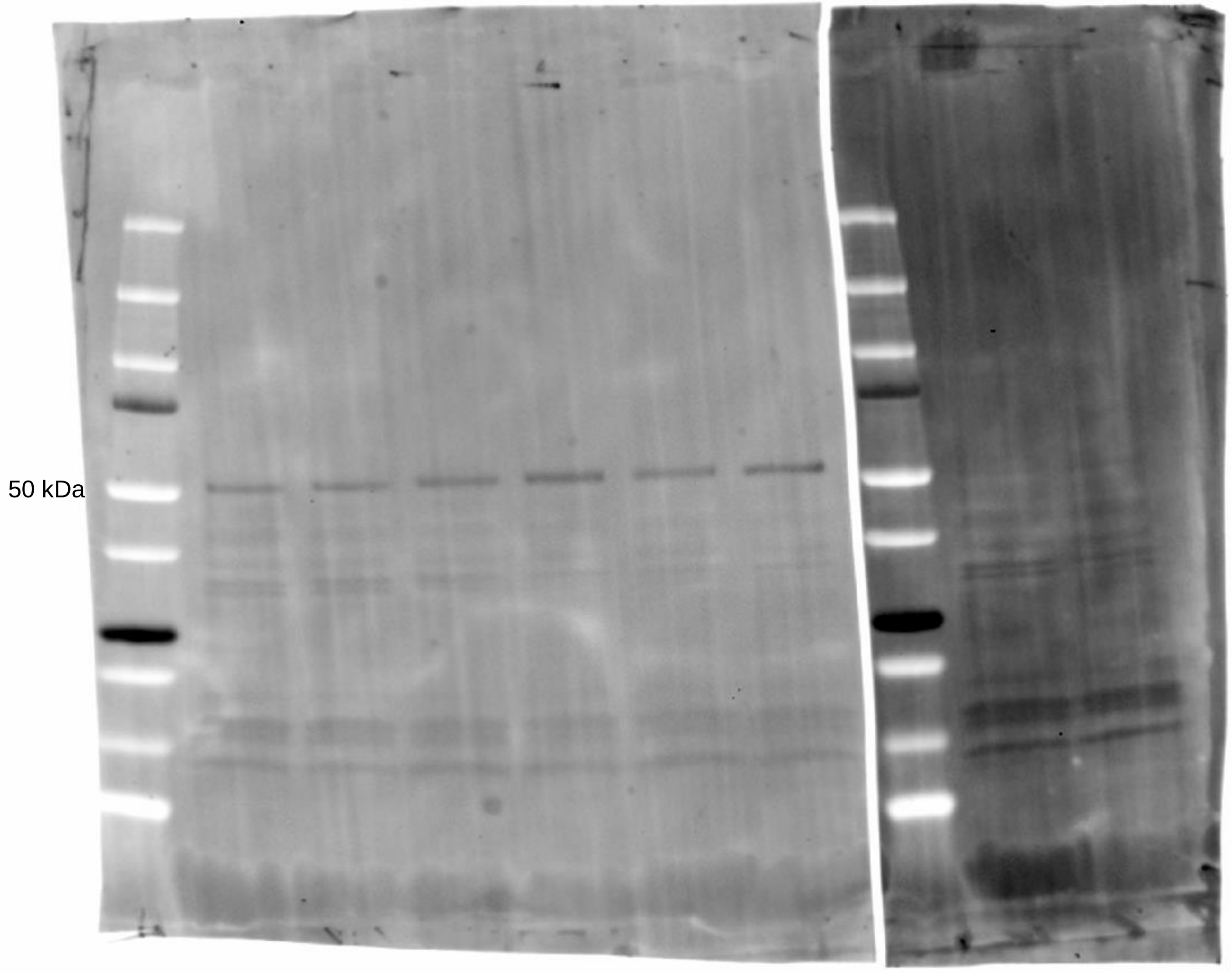

Same blot as previous slide but with molecular weight ladders (Precision Plus Protein Dual Color Standard (BioRad Cat #: 1610374)

Figure 2 Panel D

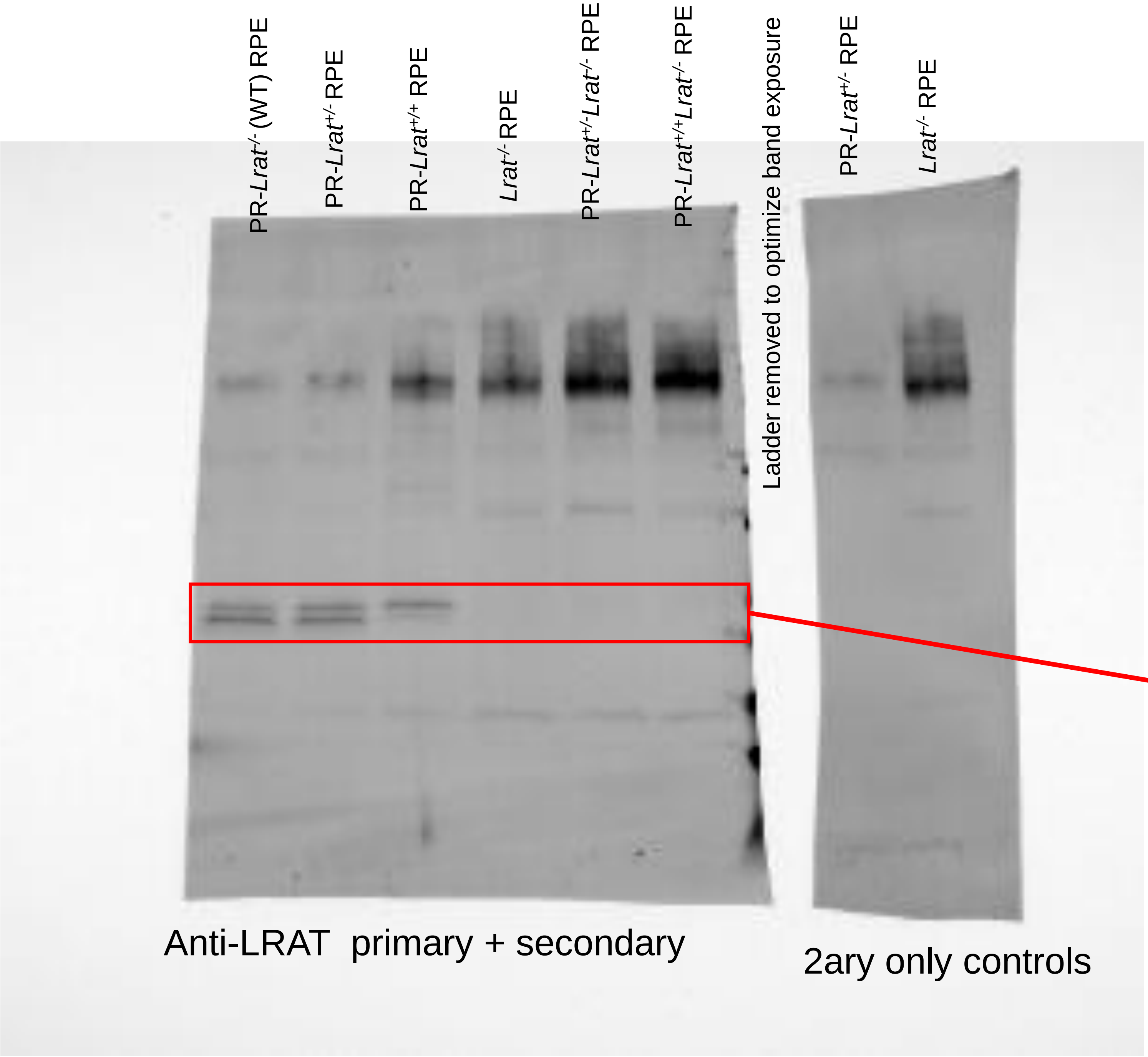

As in Figure 2 Panel D:

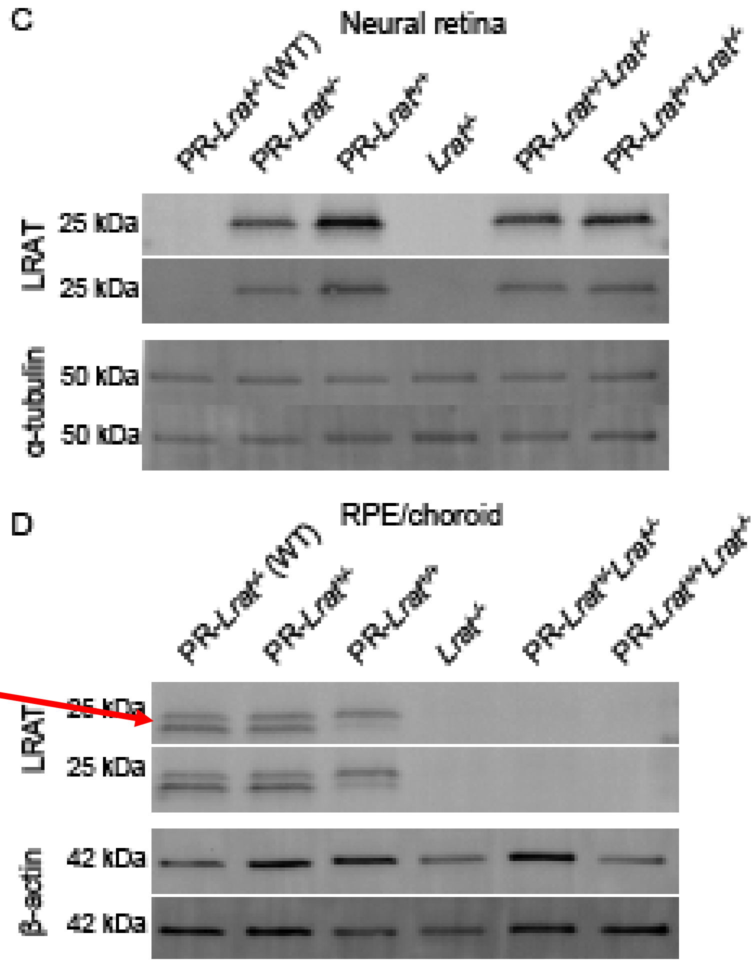

*See same blot w/ ladder on n[illegible]

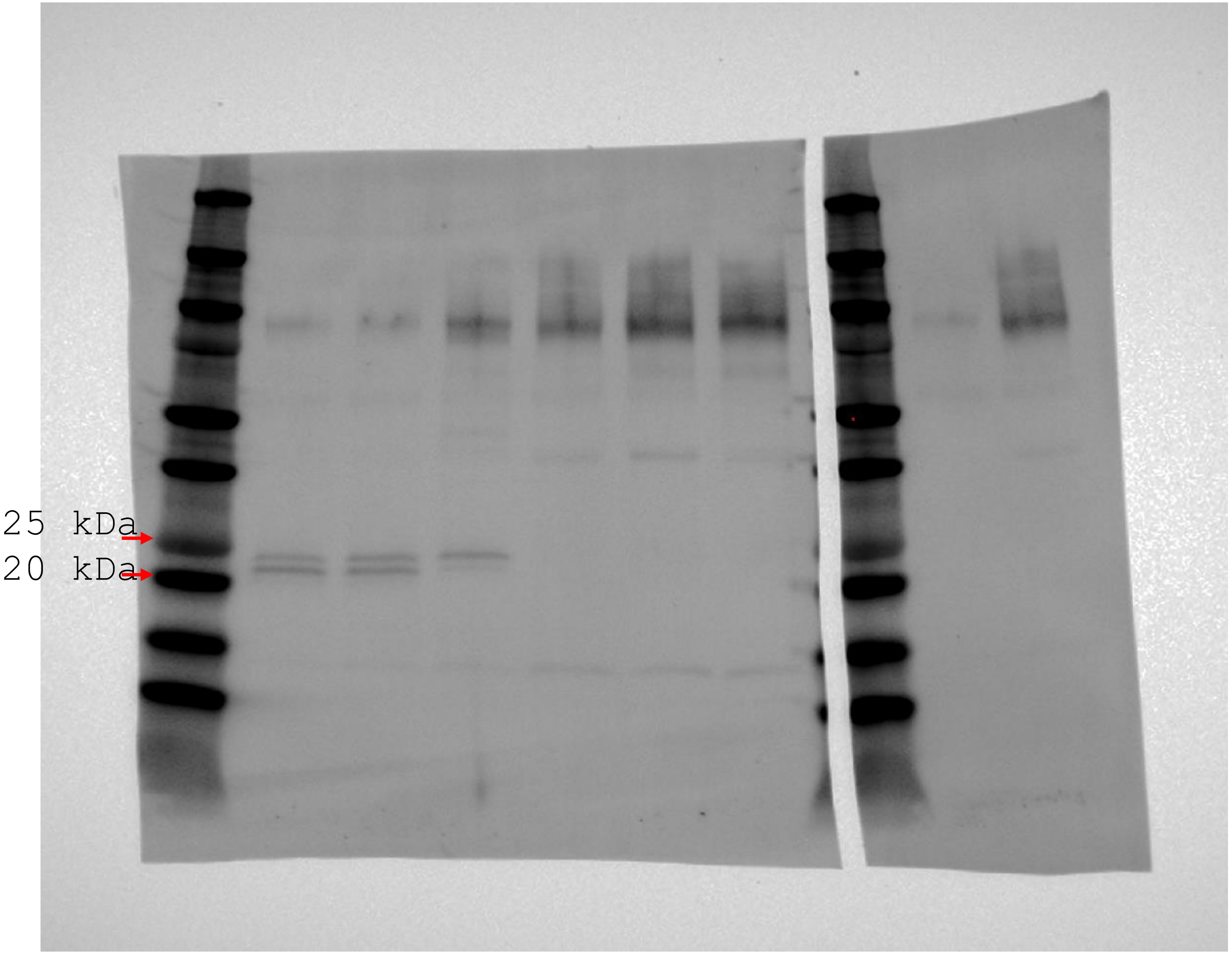

Same blot as previous slide but with molecular weight ladders (Precision Plus Protein Dual Color Standard (BioRad Cat #: 1610374)

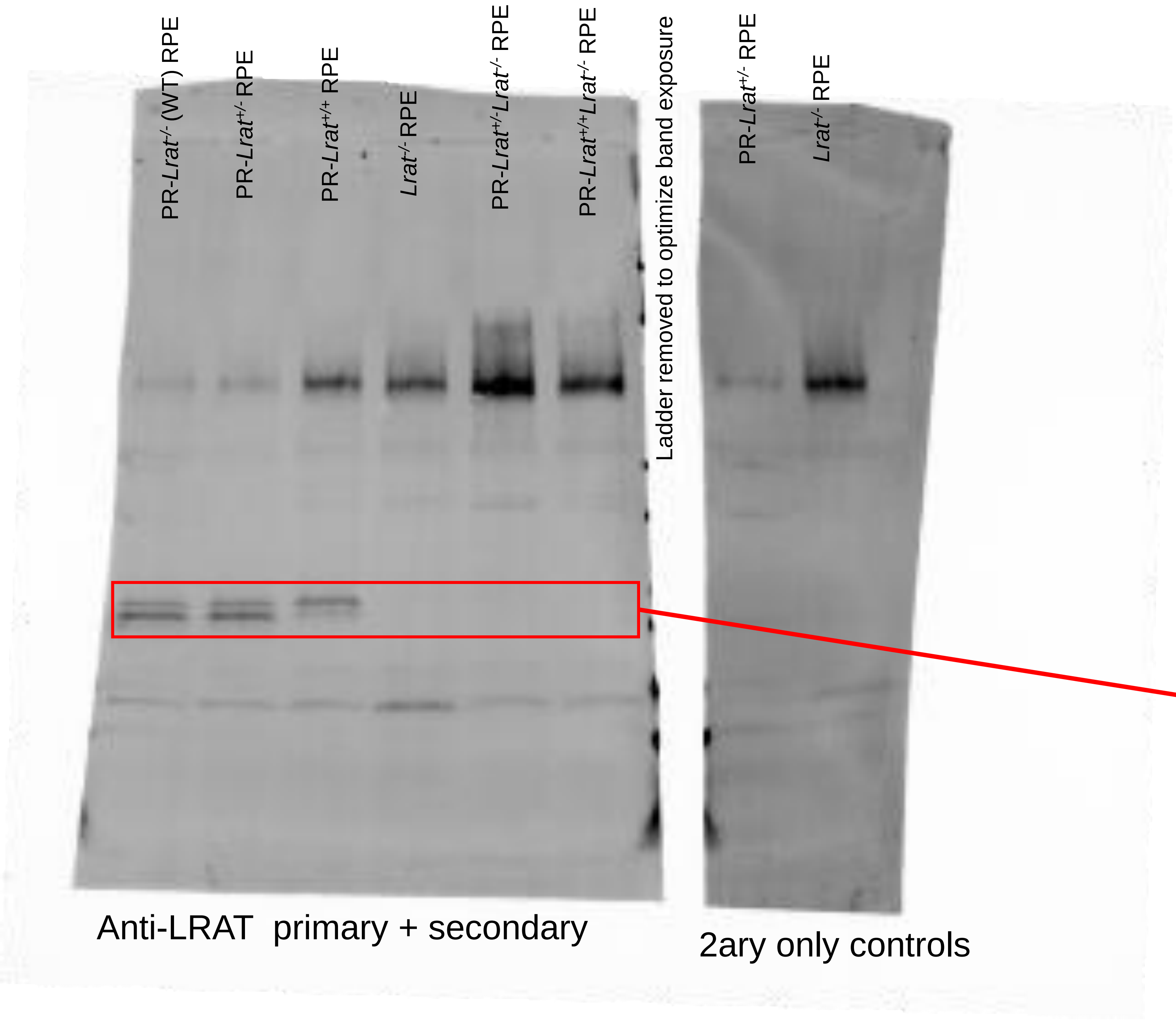

As in Figure 2 Panel D:

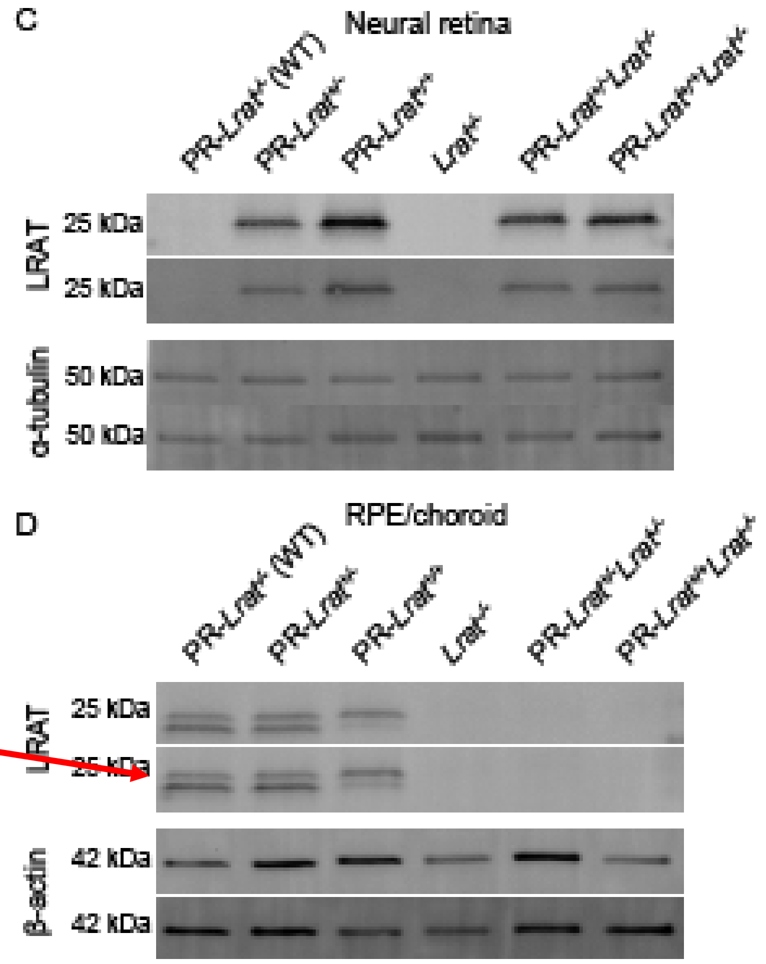

*See same blot w/ ladder on n[illegible]

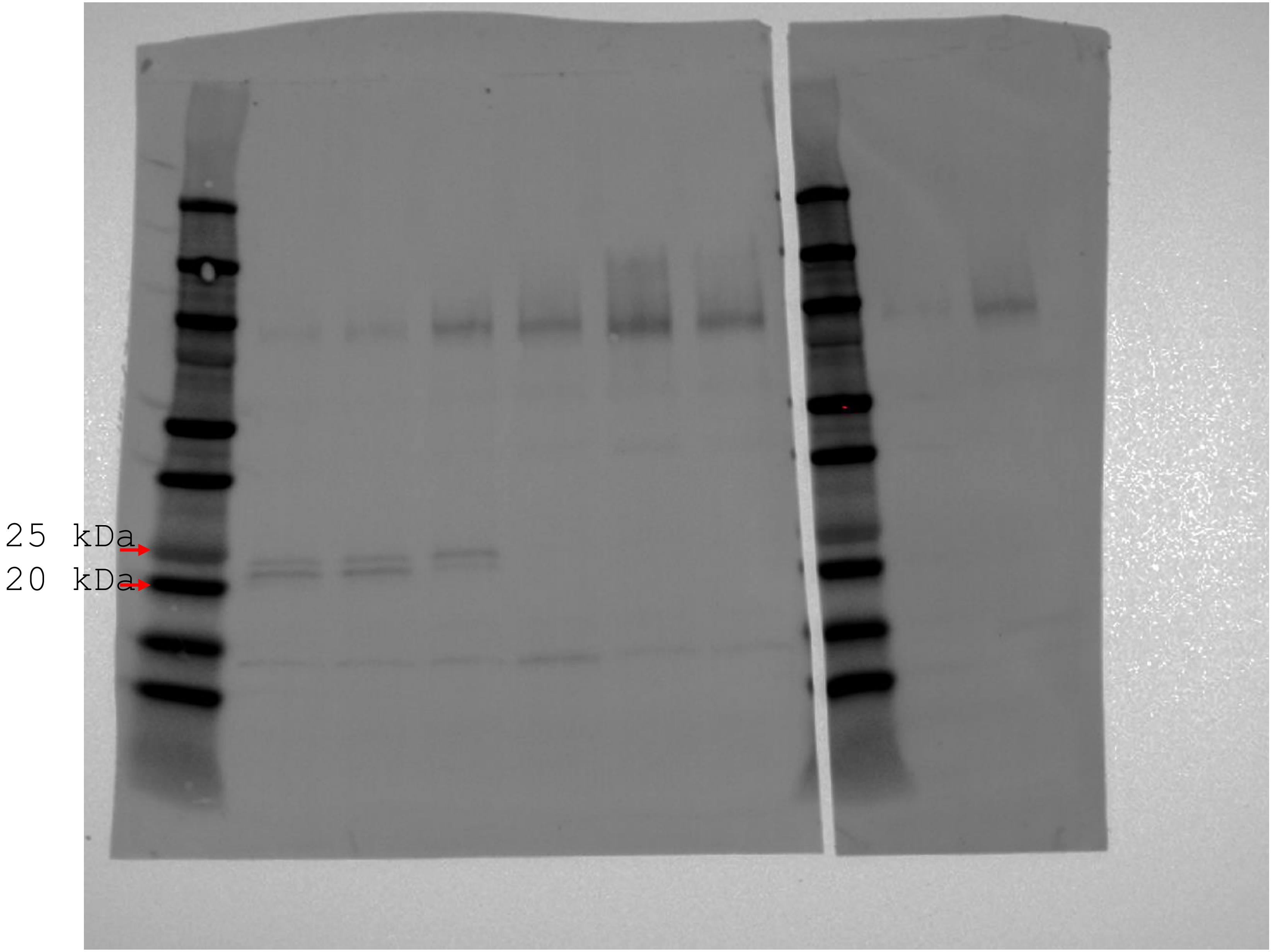

Same blot as previous slide but with molecular weight ladders (Precision Plus Protein Dual Color Standard (BioRad Cat #: 1610374)

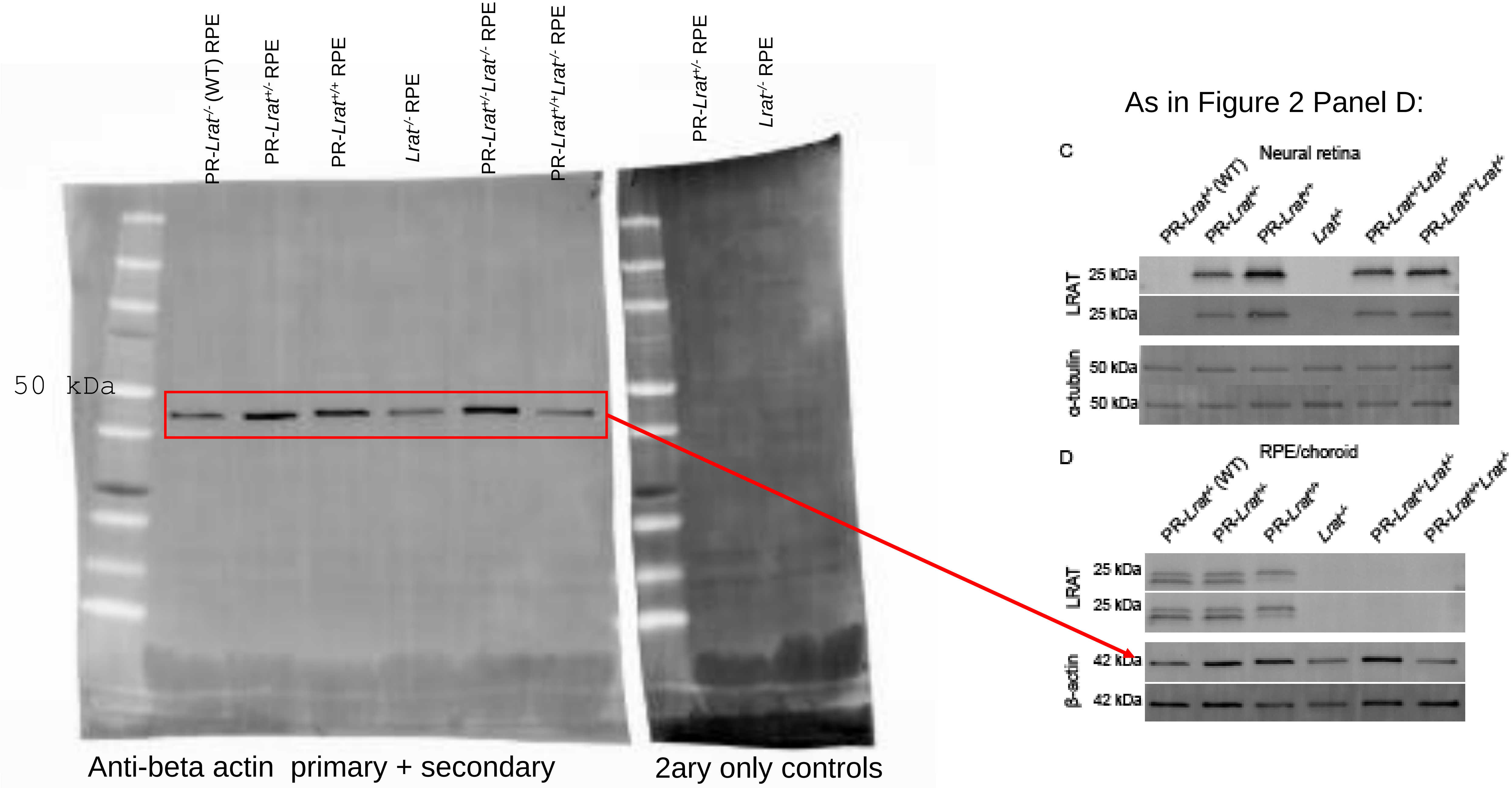

*Different antibody host species, secondary, channel used for beta actin ; same blot as featured in slides 12 + 13 staining for anti LRAT.

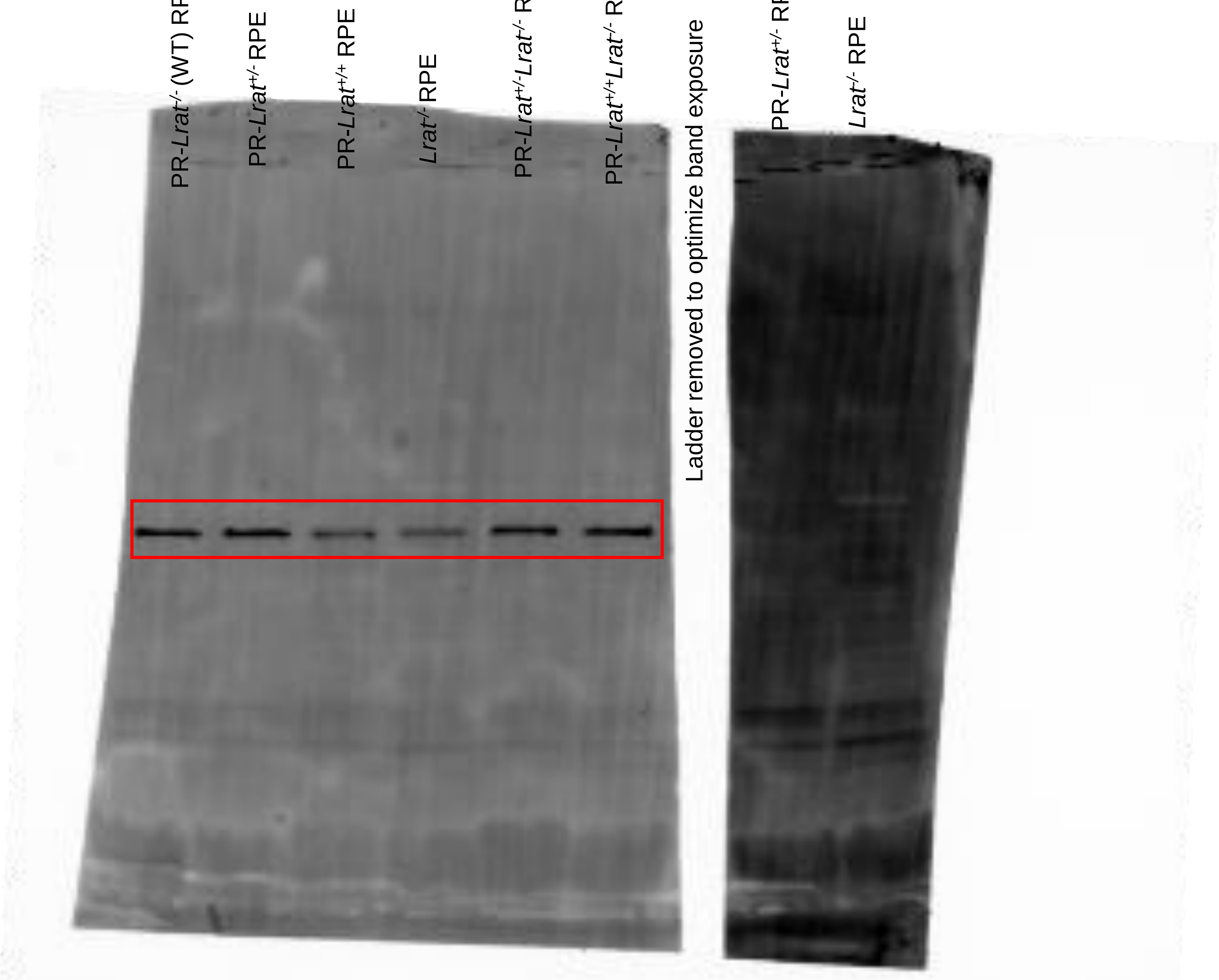

As in Figure 2 Panel D:

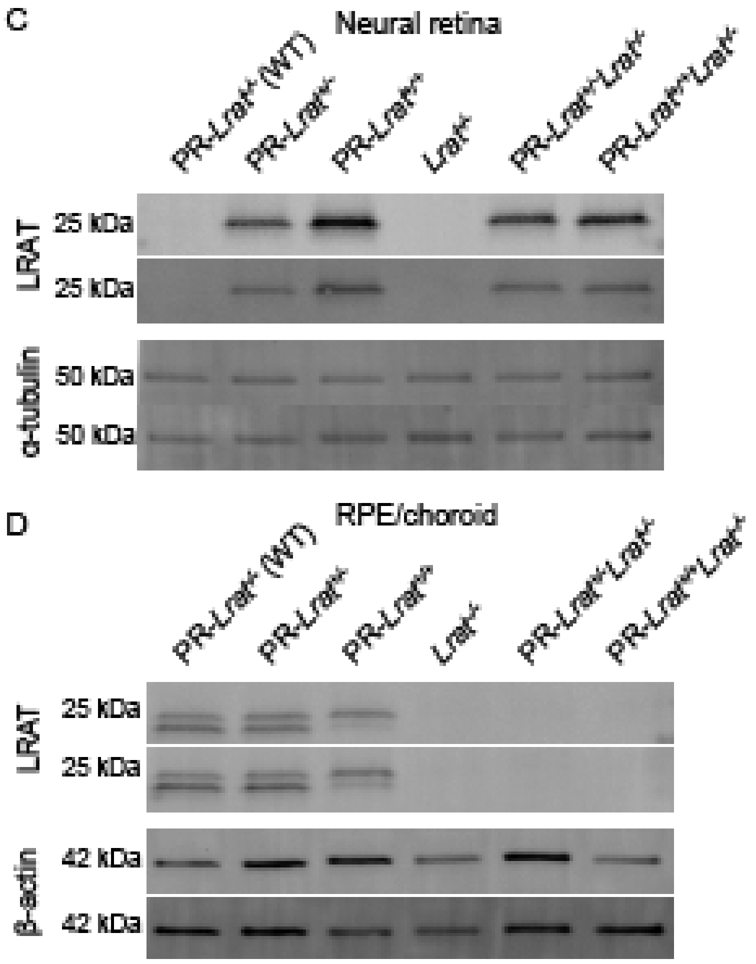

*Different antibody host species, secondary, channel used for beta actin ; same blot as featured in slides 14 + 15 staining for anti LRAT.

*See same blot w/ ladder on ne

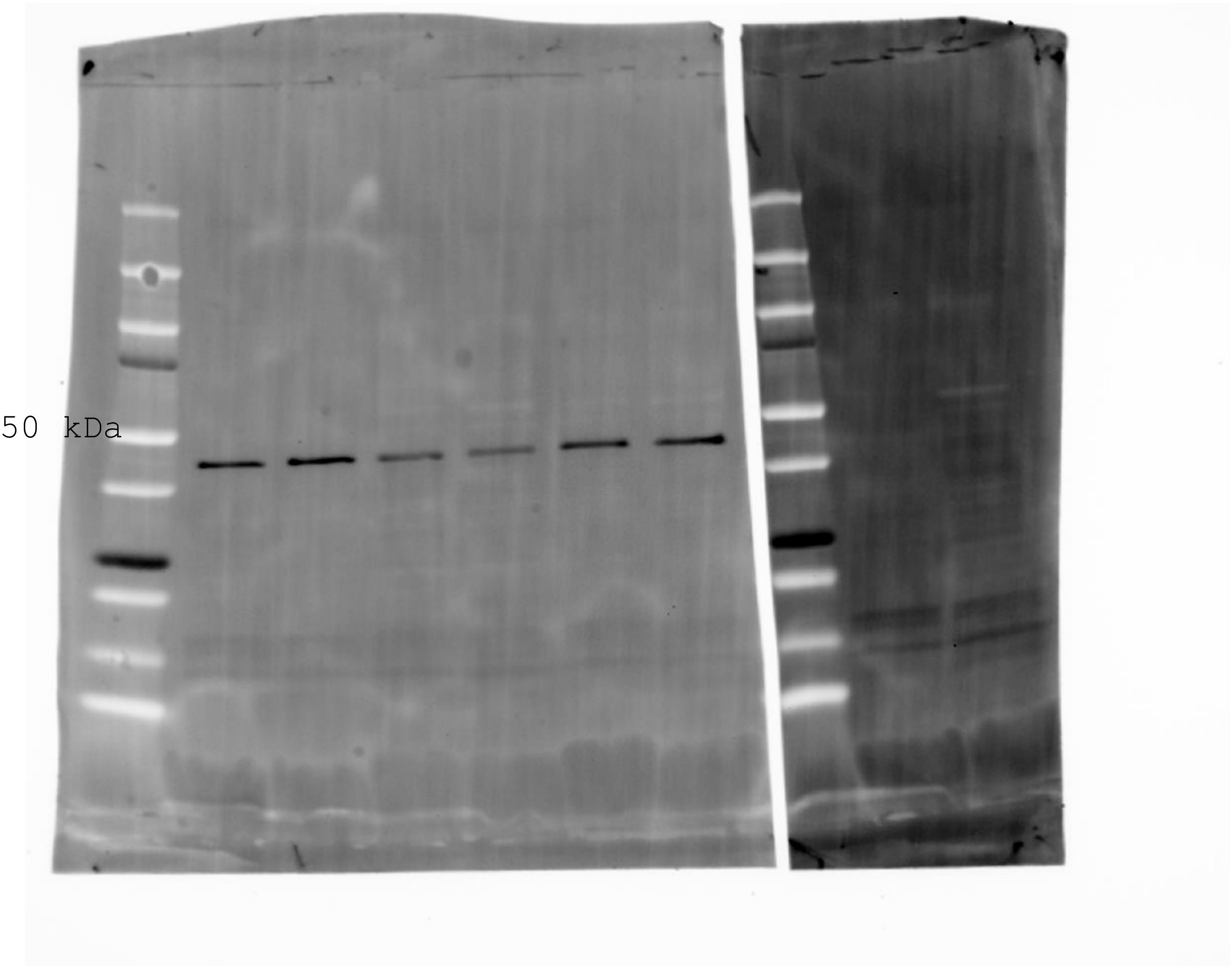

Same blot as previous slide but with molecular weight ladders (Precision Plus Protein Dual Color Standard (BioRad Cat #: 1610374)

Figure 6 Panel I

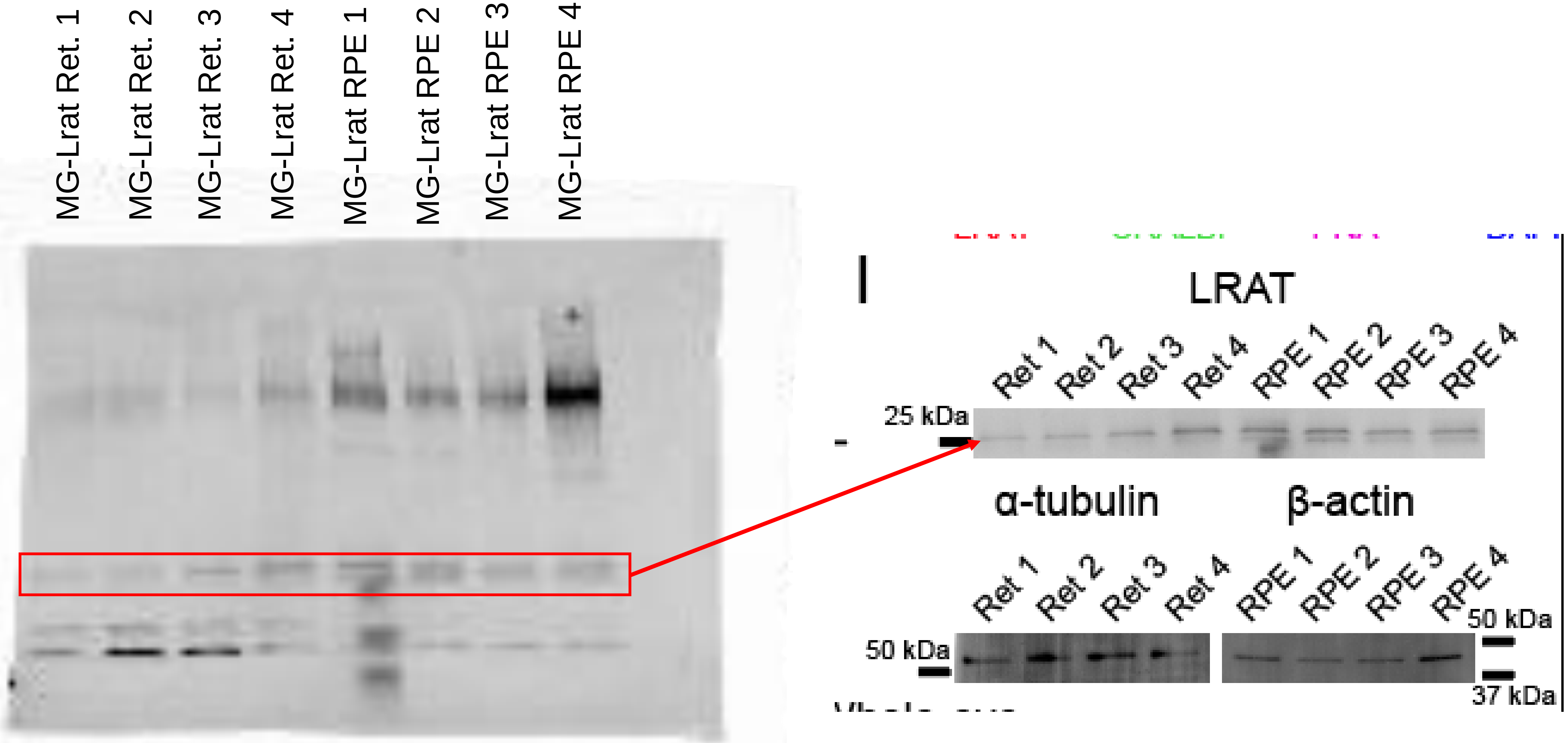

Anti-LRAT primary + secondary

Ladder removed to optimize band exposure; see next slide for ladder inclusion

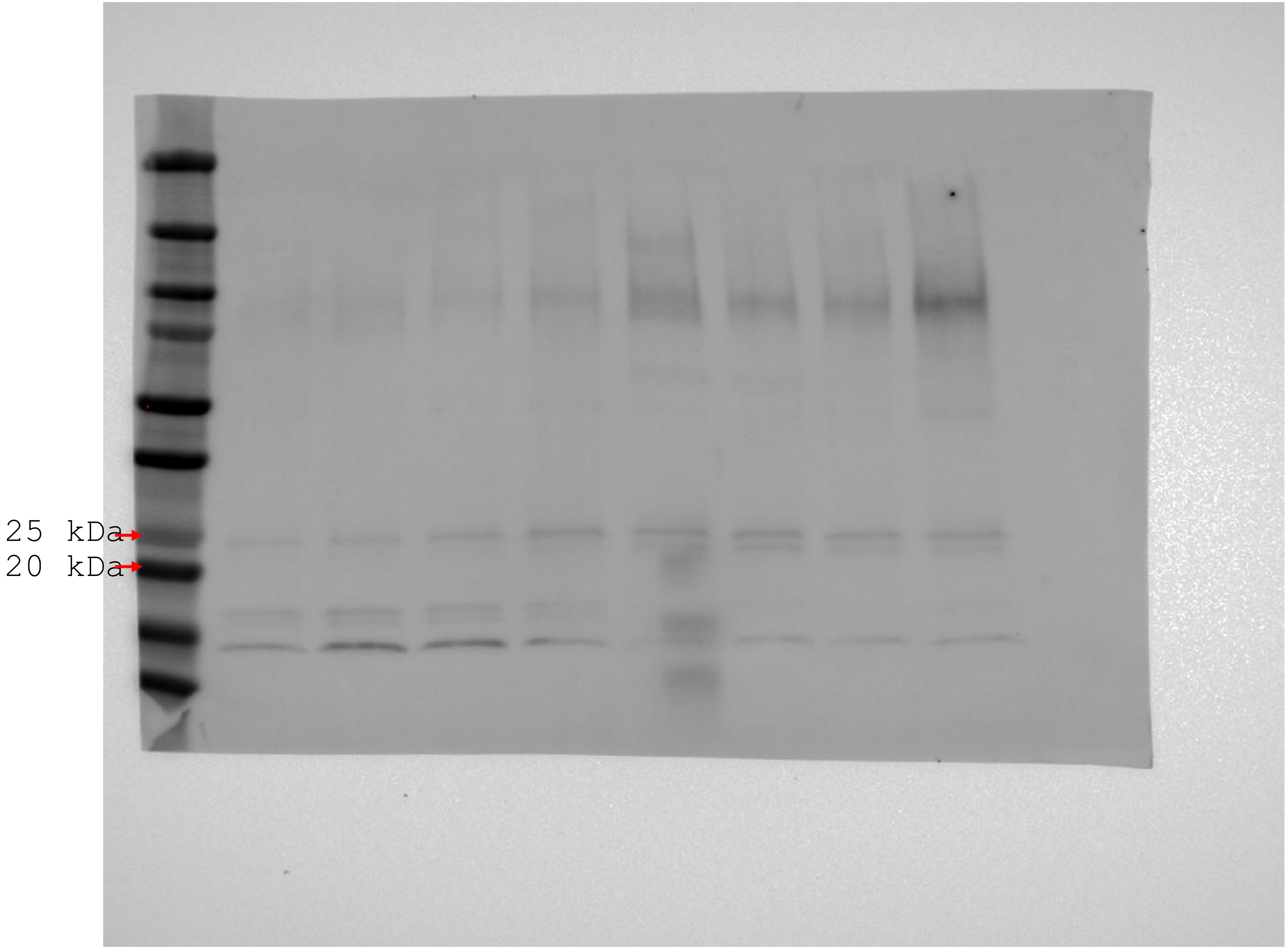

Same blot as previous slide but with molecular weight ladders (Precision Plus Protein Dual Color Standard (BioRad Cat #: 1610374)

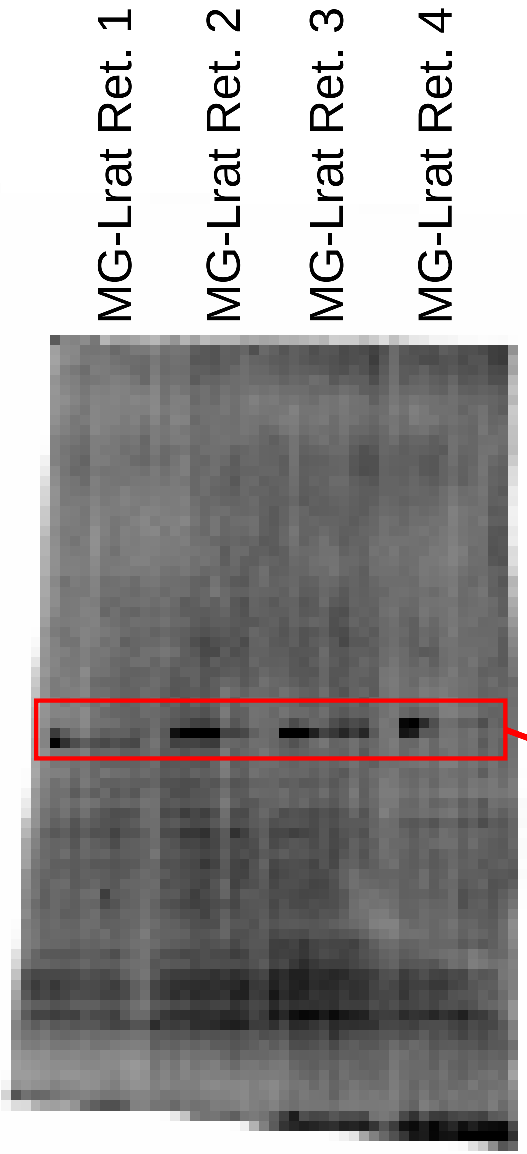

Anti-alpha tubulin primary + secondary

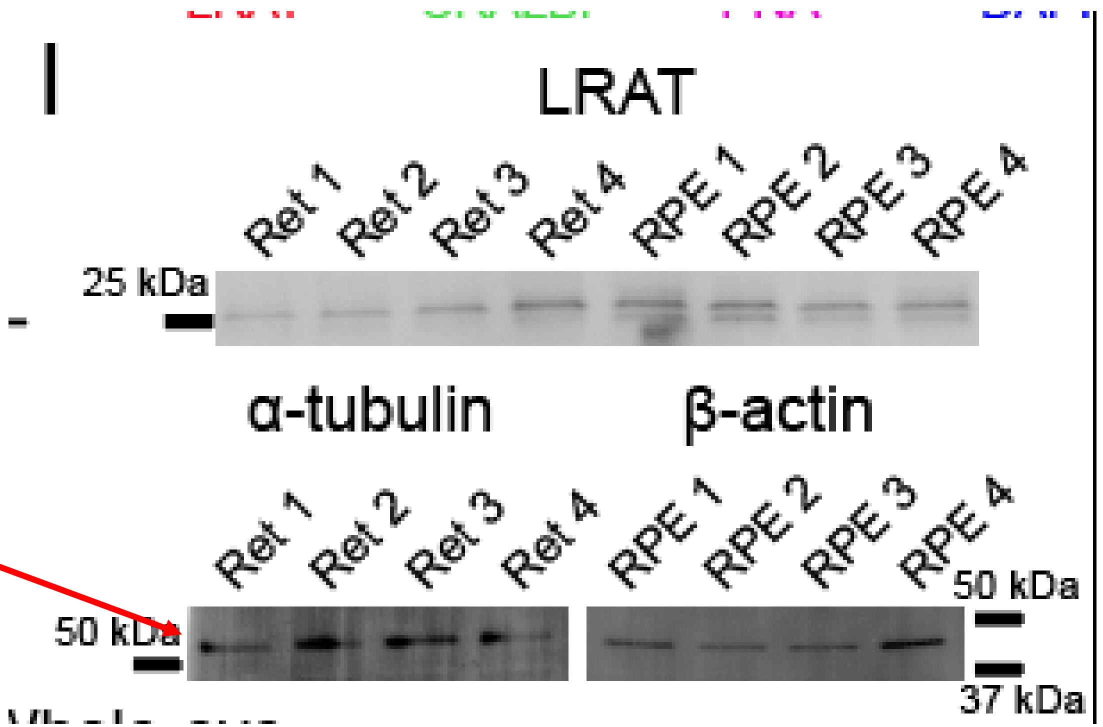

Ladder removed to optimize band exposure; see next slide for ladder inclusion

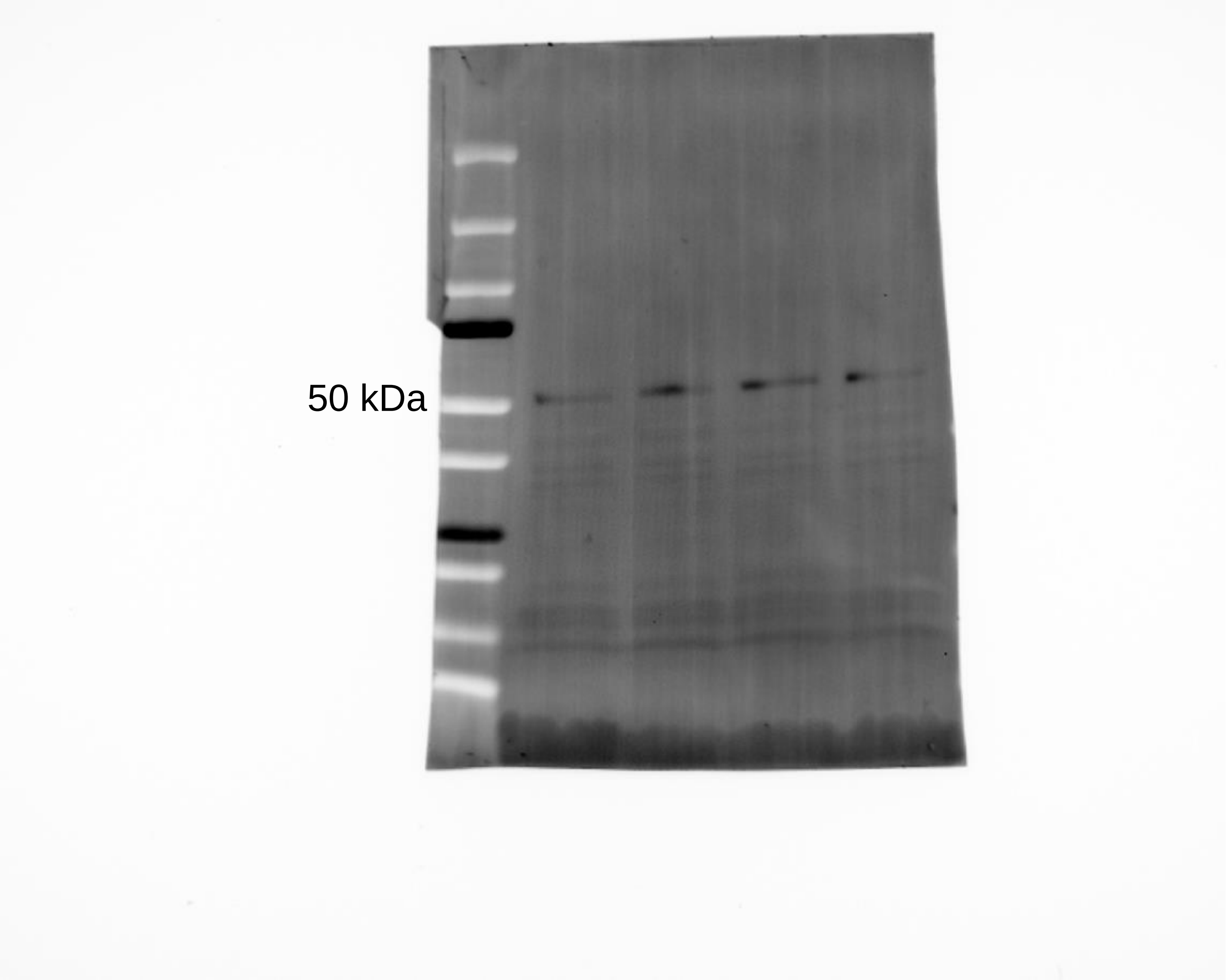

Same blot as previous slide but with molecular weight ladders (Precision Plus Protein Dual Color Standard (BioRad Cat #: 1610374)

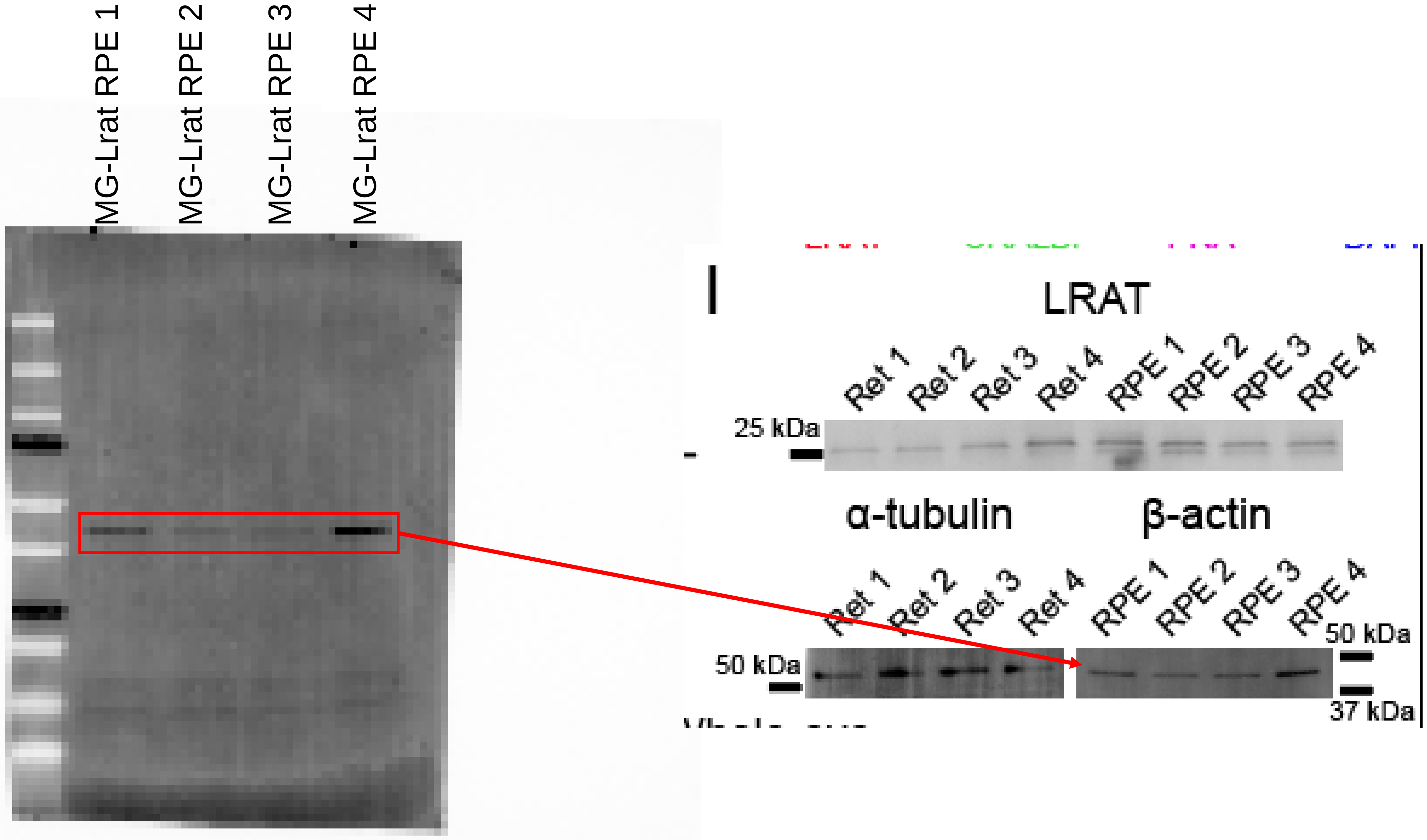

Anti-beta actin primary + secondary

Figure 9 Panel F

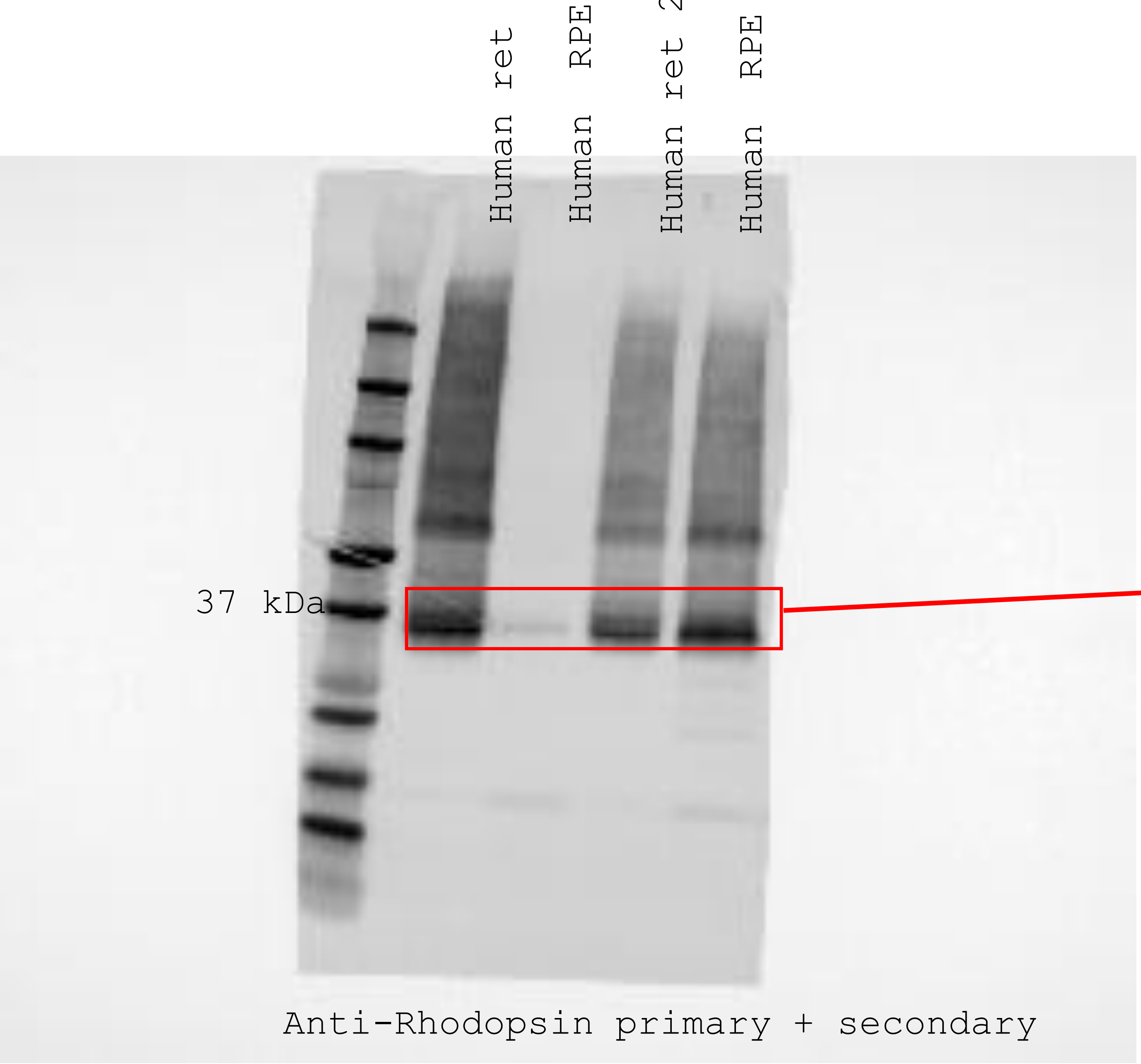

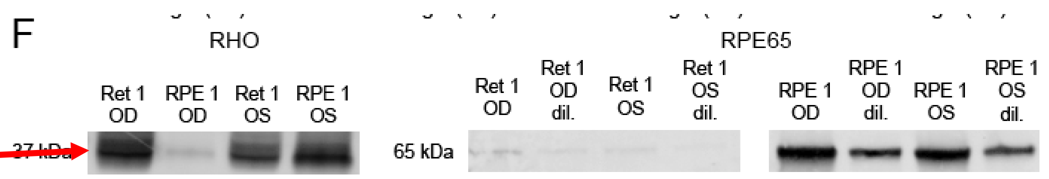

With molecular weight ladder (Precision Plus Protein Dual Color Standard (BioRad Cat #: 1610374)

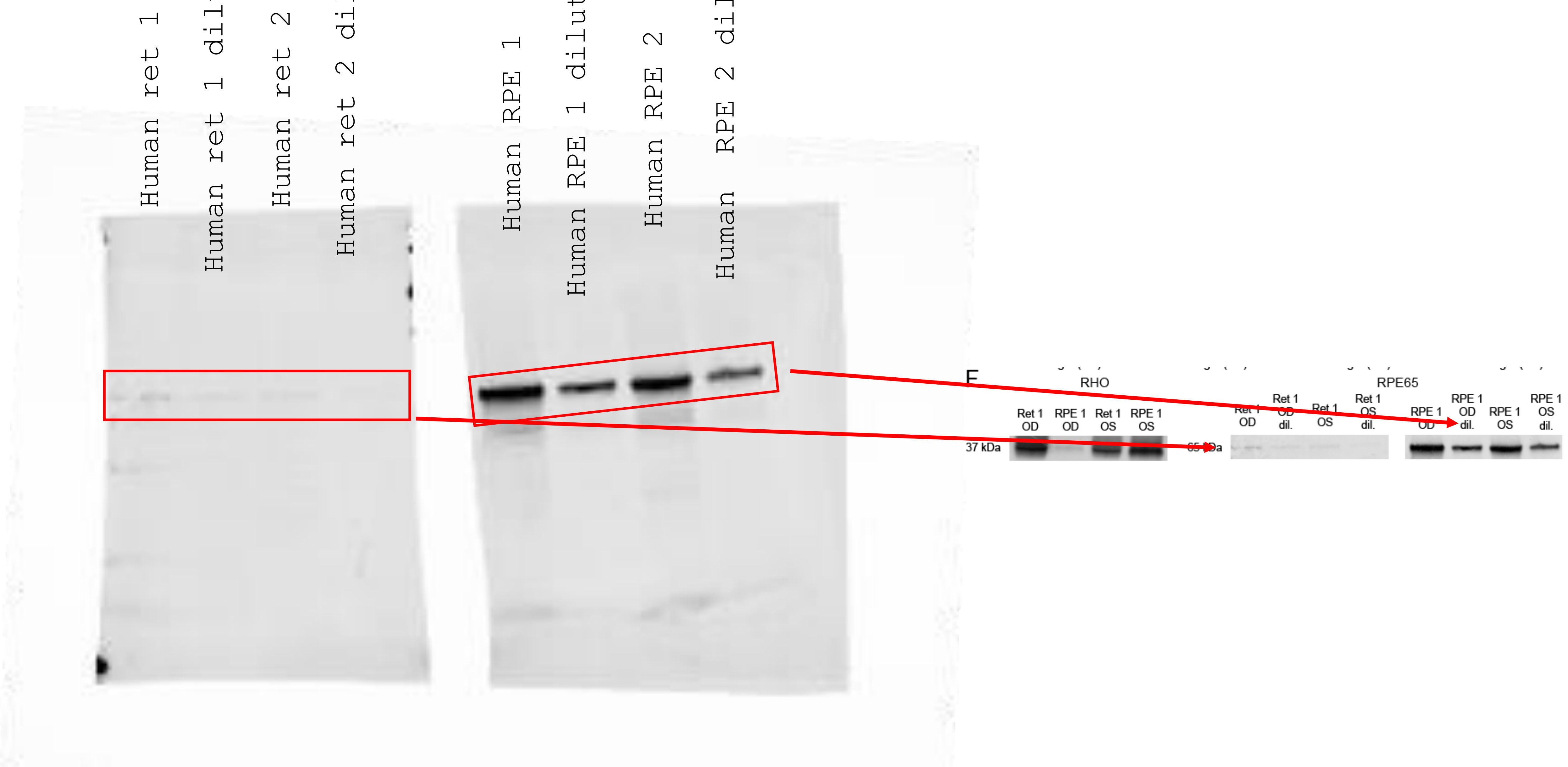

Anti-RPE65 primary + secondary

Ladder removed to optimize band exposure; see next slide for ladder inclusion

With molecular weight ladders (Precision Plus Protein Dual Color Standard (BioRad Cat #: 1610374)

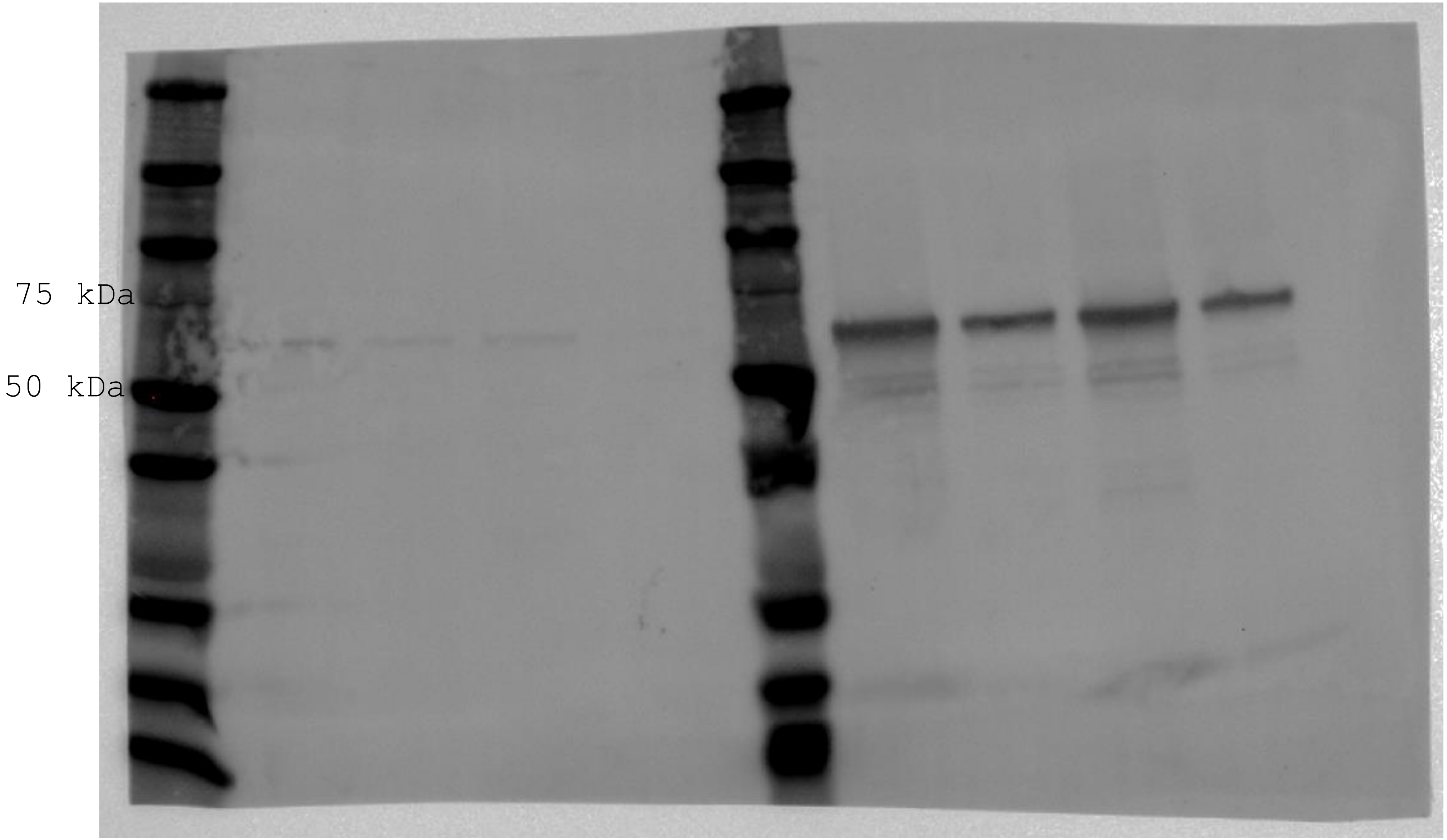

Supplementary Figure 3 Panel B

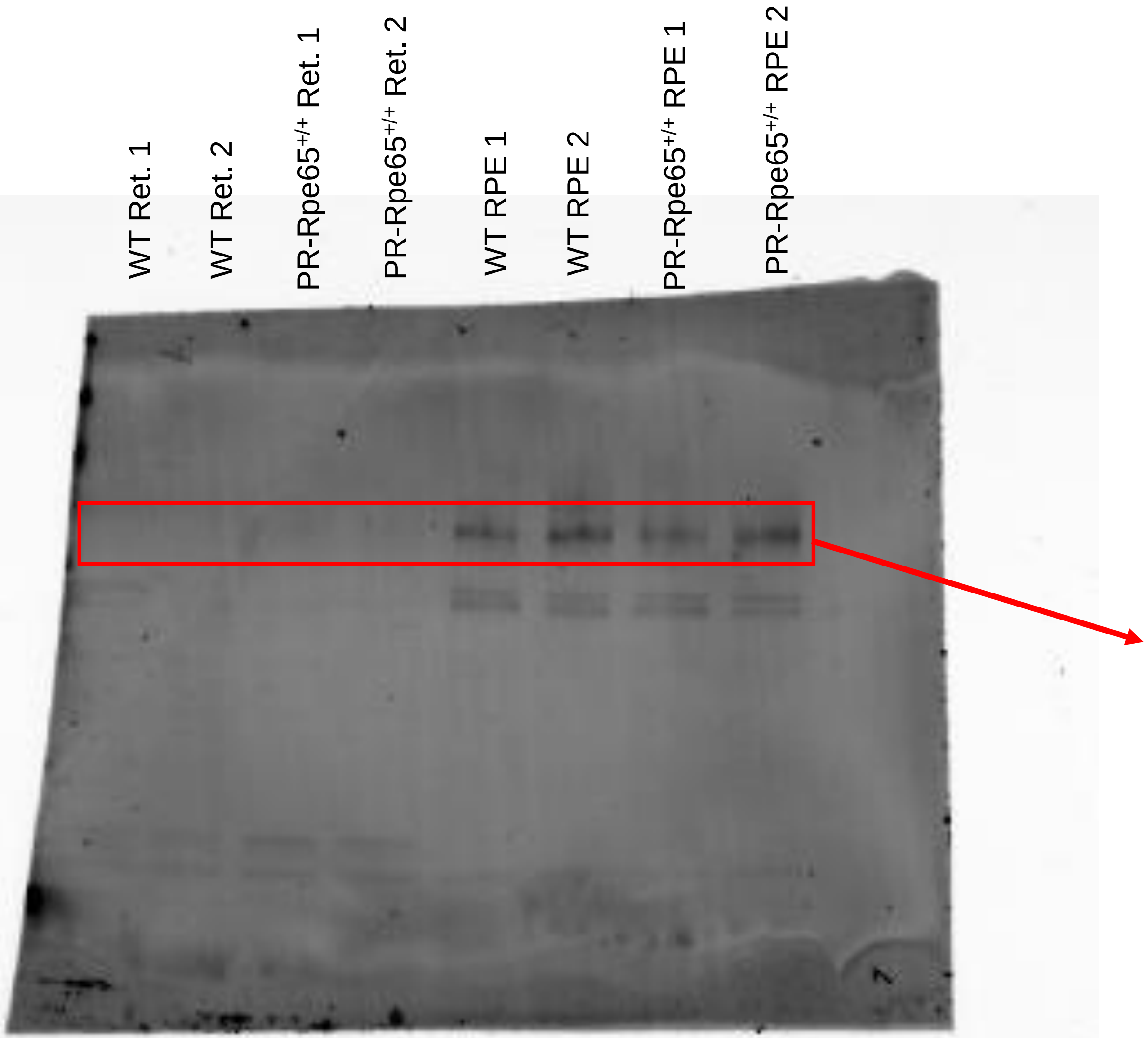

Anti-RPE65 primary + secondary

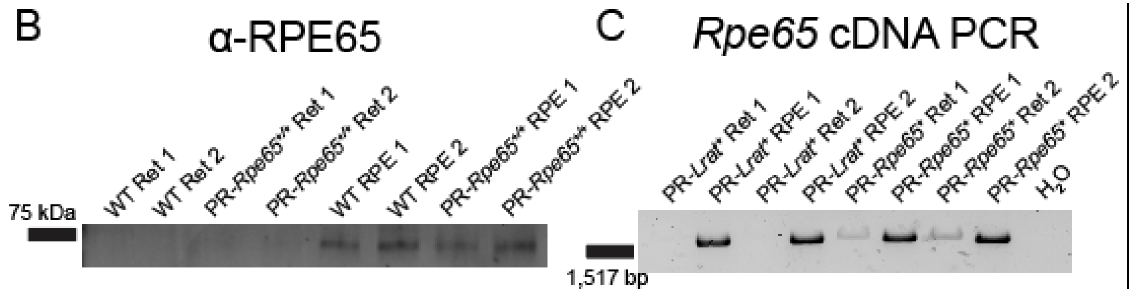

Ladder removed to optimize band exposure; see next slide for ladder inclusion

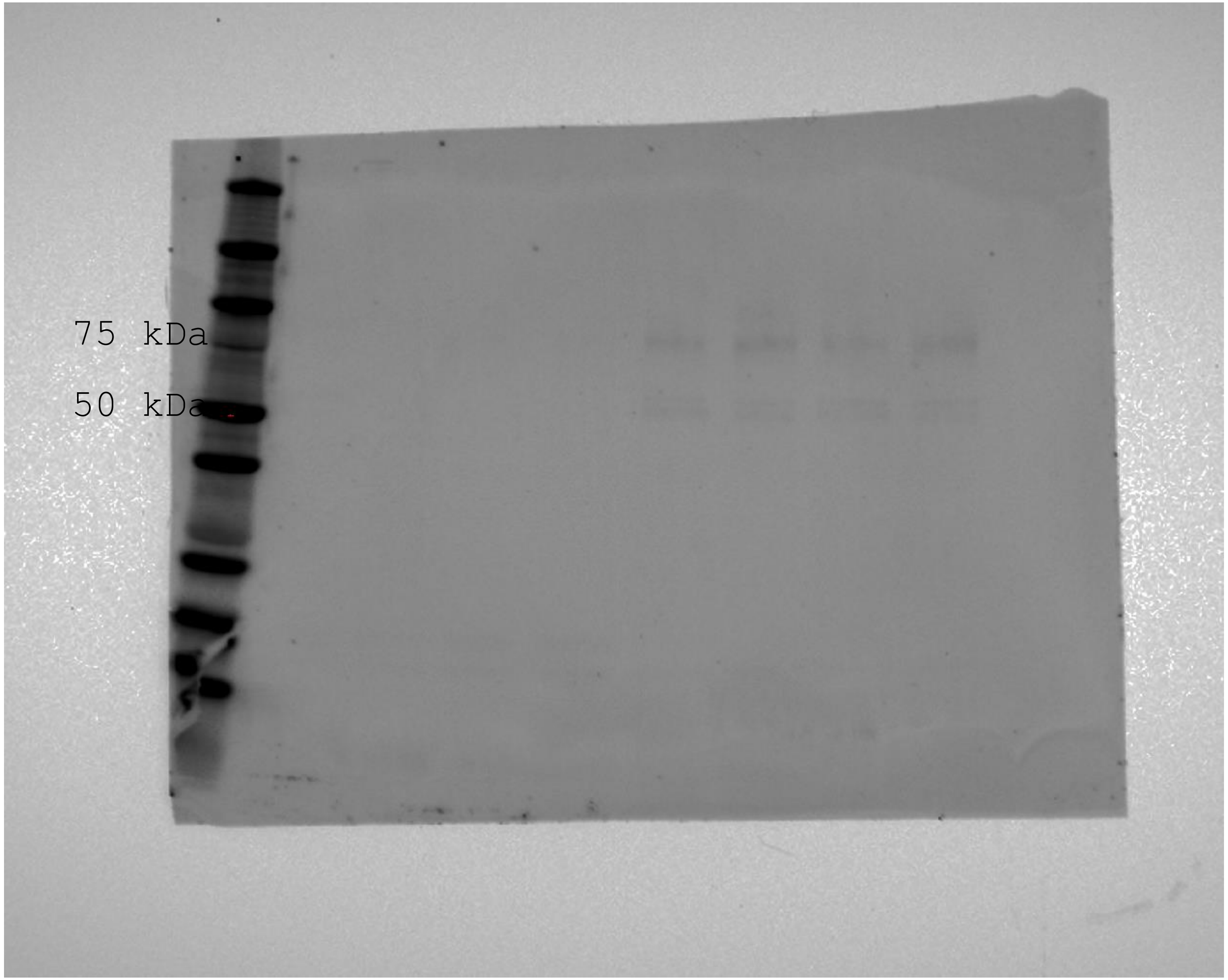

Same blot as previous slide but with molecular weight ladders (Precision Plus Protein Dual Color Standard (BioRad Cat #: 1610374)

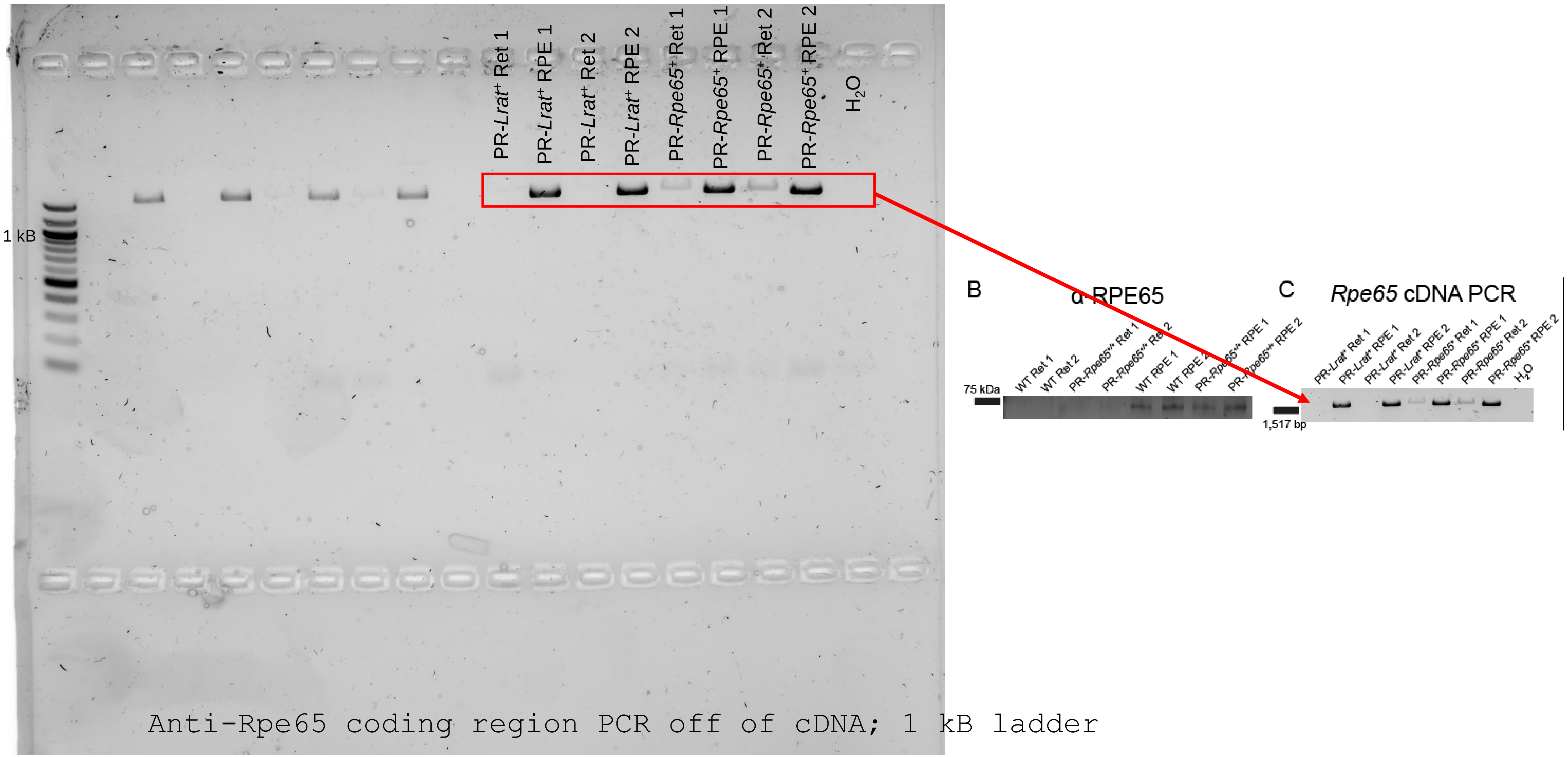

Anti-Rpe65 coding region PCR off of cDNA; 1 kB ladder

Supplementary Figure 16 Panel A

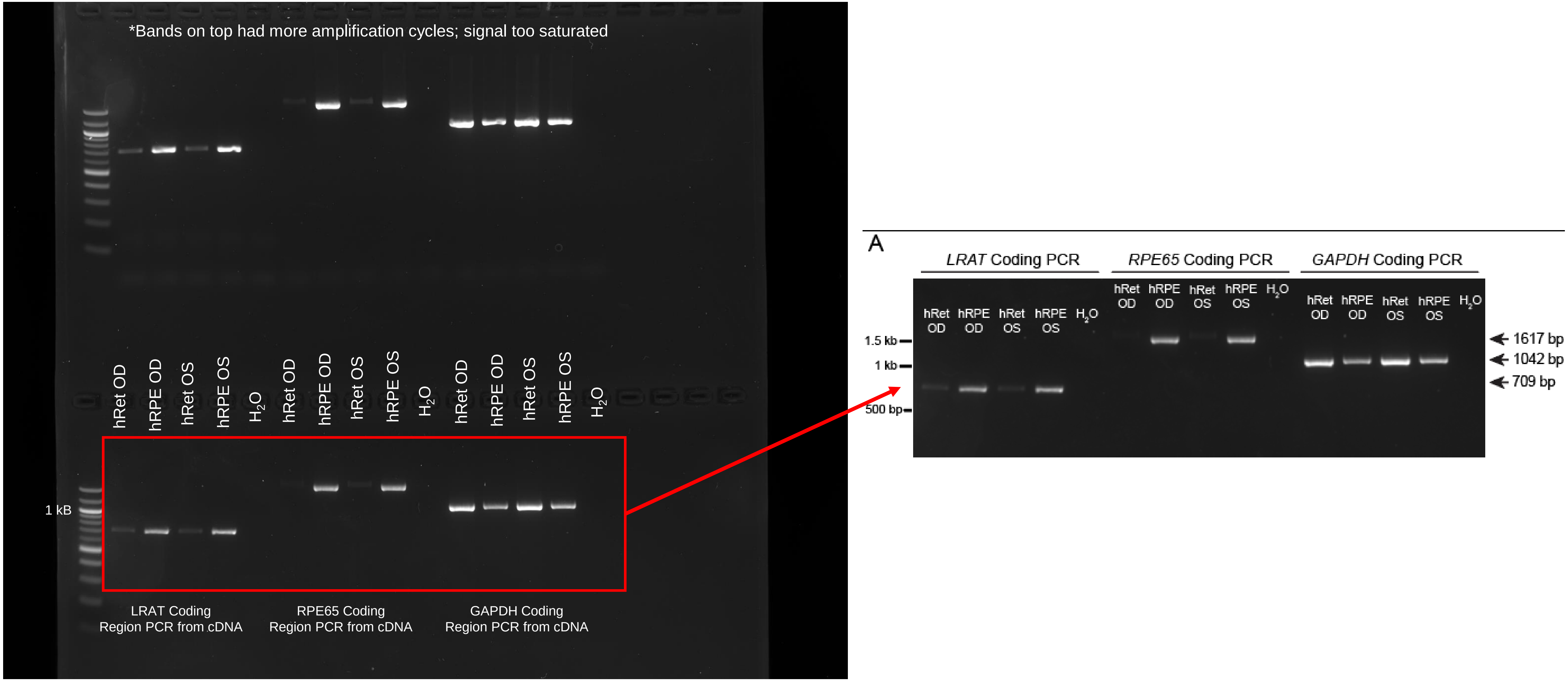

*Bands on top had more amplification cycles; signal too saturated
hRet OD
hRPE OD
hRet OS
hRPE OS
H_2O
hRet OD
hRPE OD
hRet OS
hRPE OS
H_2O
hRet OD
hRPE OD
hRet OS
hRPE OS
H_2O
1 kB
LRAT Coding Region PCR from cDNA
RPE65 Coding Region PCR from cDNA
GAPDH Coding Region PCR from cDNA
A
LRAT Coding PCR
RPE65 Coding PCR
GAPDH Coding PCR
hRet OD
hRPE OD
hRet OS
hRPE OS
H_2O
hRet OD
hRPE OD
hRet OS
hRPE OS
H_2O
hRet OD
hRPE OD
hRet OS
hRPE OS
H_2O
1.5 kb
1 kb
500 bp
1617 bp
1042 bp
709 bp

Figure 2 Panels A-B
Immunohistochemistry

Figure 2 Panel A: Uncropped images + secondary only controls

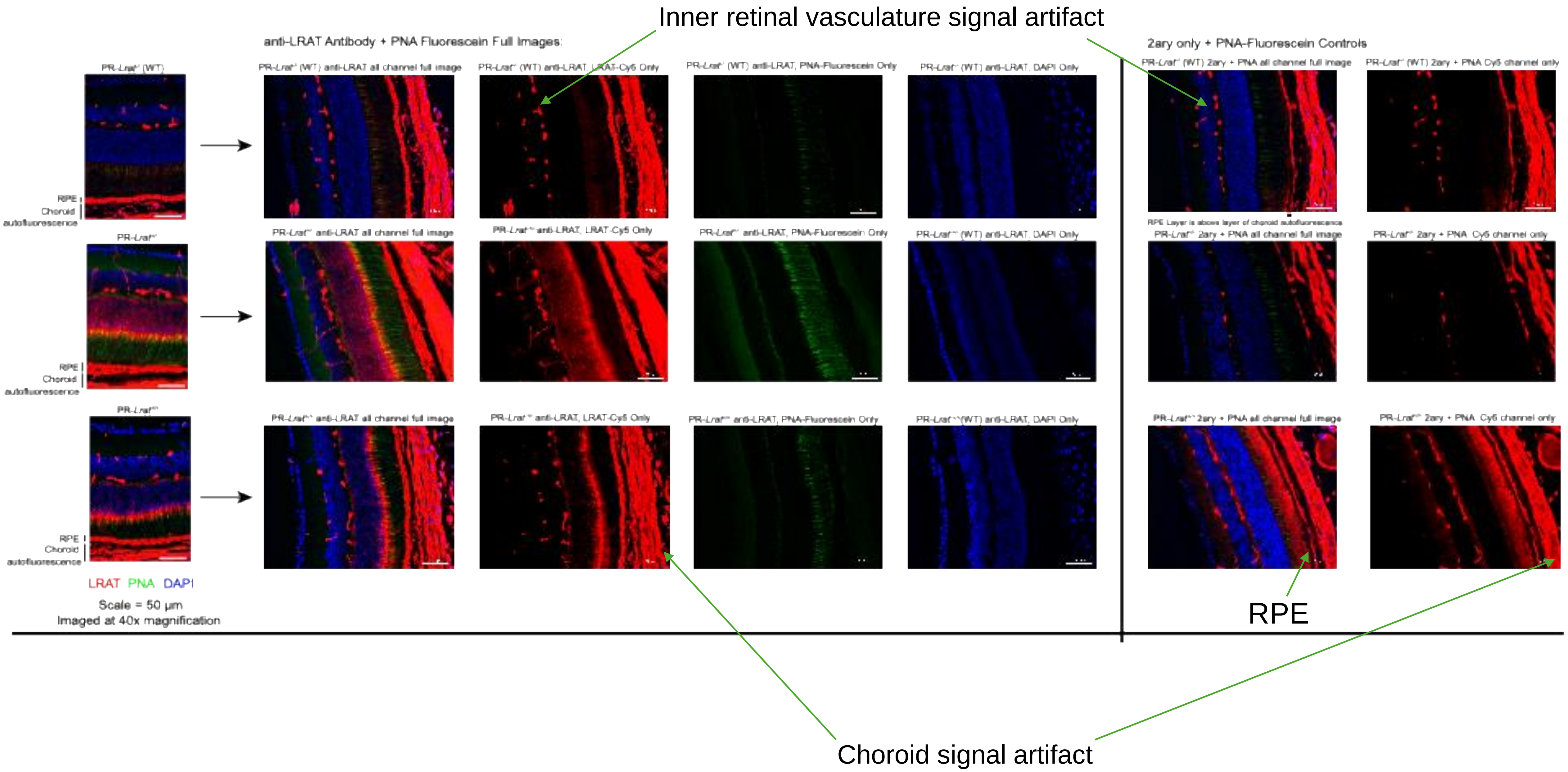

Figure 2 Panel B: Uncropped images

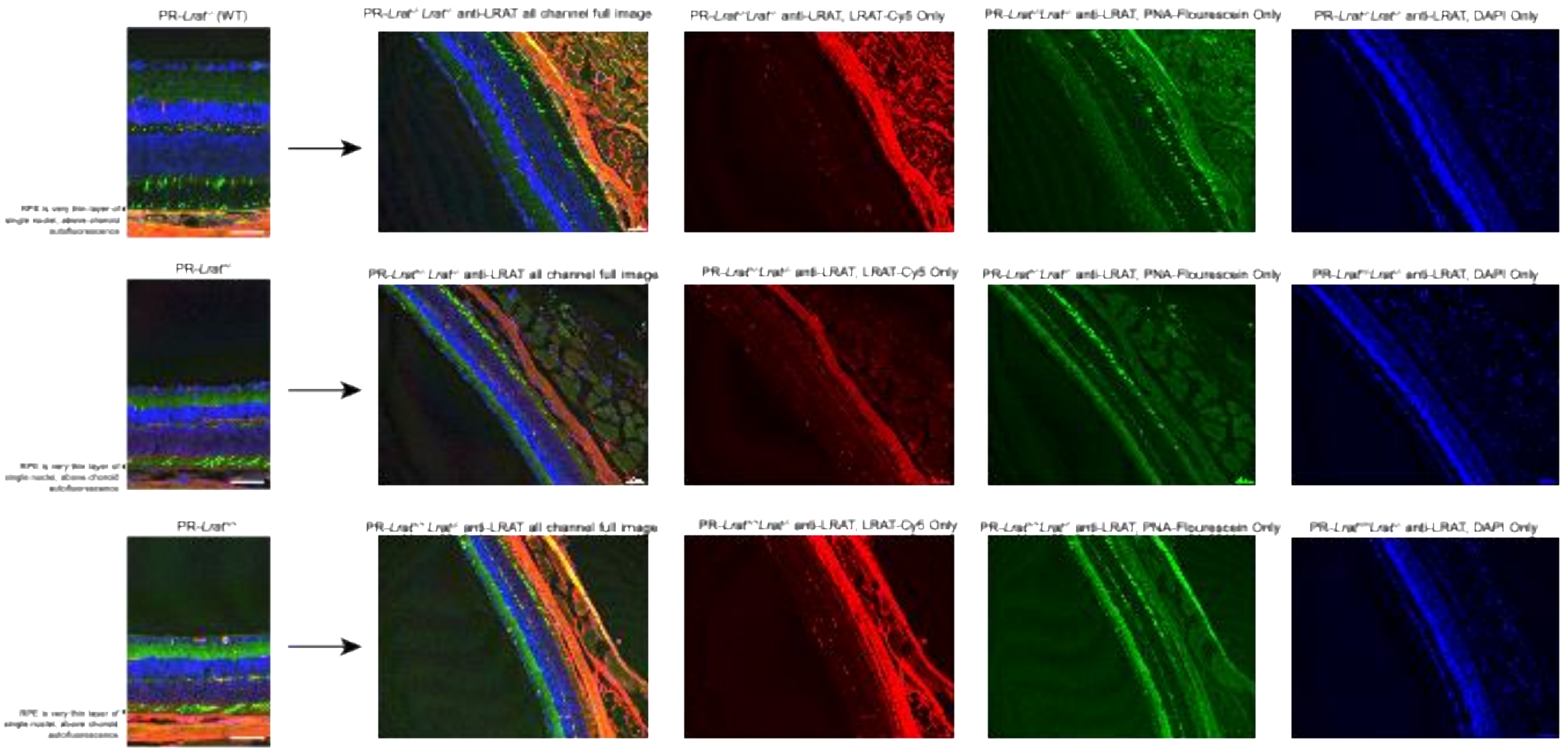

LRAT PNA DAPI

Scale = 50 µm

*Images taken at 20x magnification

No secondary only controls performed for these samples
